# Concurrent targeting of glycolysis in bacteria and host cell inflammation in septic arthritis

**DOI:** 10.15252/emmm.202115284

**Published:** 2022-11-10

**Authors:** Hyuk‐Kwon Kwon, Kristin E Yu, Sean V Cahill, Kareme D Alder, Christopher M Dussik, Sang‐Hun Kim, Lokesh Sharma, Jungho Back, Irvin Oh, Francis Y Lee

**Affiliations:** ^1^ Department of Orthopaedics and Rehabilitation, School of Medicine Yale University New Haven CT USA; ^2^ Department of Orthopedic Surgery Mayo Clinic Rochester MN USA; ^3^ Department of Orthopedic Surgery Washington University School of Medicine St. Louis MO USA; ^4^ Department of Orthopaedics and Rehabilitation University of Rochester Rochester NY USA; ^5^ Section of Pulmonary, Critical Care and Sleep Medicine, Department of Internal Medicine Yale School of Medicine New Haven CT USA

**Keywords:** dimethyl fumarate, glycolysis, inflammation, MRSA, septic arthritis, Microbiology, Virology & Host Pathogen Interaction, Musculoskeletal System

## Abstract

Intracellular infiltration of bacteria into host cells complicates medical and surgical treatment of bacterial joint infections. Unlike soft tissue infections, septic arthritis and infection‐associated inflammation destroy cartilage that does not regenerate once damaged. Herein, we show that glycolytic pathways are shared by methicillin‐resistant *Staphylococcus aureus* (MRSA) proliferation and host inflammatory machinery in septic arthritis. MRSA readily penetrates host cells and induces proinflammatory cascades that persist after conventional antibiotic treatment. The glycolysis‐targeting drug dimethyl fumarate (DMF) showed both bacteriostatic and anti‐inflammatory effects by hindering the proliferation of intracellular MRSA and dampening excessive intraarticular inflammation. Combinatorial treatment with DMF and vancomycin further reduced the proliferation and re‐emergence of intracellular MRSA. Combinatorial adjuvant administration of DMF with antibiotics alleviated clinical symptoms of septic arthritis by suppressing bacterial burden and curbing inflammation to protect cartilage and bone. Our results provide mechanistic insight into the regulation of glycolysis in the context of infection and host inflammation toward development of a novel therapeutic paradigm to ameliorate joint bioburden and destruction in septic arthritis.

## Graphical abstract

Septic arthritis has limitations such as intracellular bacteria and inflammatory tissue disruption even after conventional treatment. This study shows evidence of the potential of repurposing of glycolysis targeting DMF in conjunction with a conventional antibiotic to ameliorate septic arthritis.

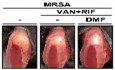

## Introduction

Septic arthritis is a bacterial infection of the joint space that spreads via the bloodstream or through penetrating trauma and constitutes a medical and orthopedic emergency (Carpenter *et al*, 2011). The incidence of septic arthritis continues to increase due to a growing number of risk factors such as the aging of the population, the performance of a greater number of orthopedic and/or other invasive procedures, and the increased frequency of immunosuppressive therapy (Kaandorp *et al*, 1997; Mathews *et al*, 2010). *Staphylococcus aureus* (*S. aureus*) is the most common bacterial pathogen implicated in septic arthritis. Morbidity rates due to septic arthritis approach 32% among patients treated with antibiotics, with a mortality rate that has stagnated at 12% (Weston *et al*, 1999; Dubost *et al*, 2002). Although the proportion of infections caused by methicillin‐susceptible *S. aureus* (MSSA) remains constant, the development of septic arthritis‐related pathologies and mortality has worsened as the proportion of orthopedic infections caused by MRSA has increased (Martinez‐Aguilar *et al*, 2004; Arnold *et al*, 2006; Al‐Nammari *et al*, 2007). Septic arthritis recurred in 38% of patients treated with systemic antibiotics and/or surgical intervention, with higher rates of treatment failure among patients with MRSA‐induced infection (Salgado *et al*, 2007). Despite antibiotic treatment, patients with septic arthritis develop sequelae such as secondary osteomyelitis, mono‐articular arthritis, limb length discrepancy, or joint fusion (Vincent & Amirault, 1990; Wang *et al*, 2003). Numerous studies have suggested that septic arthritis recurrence may be attributable to the ability of *S. aureus* to penetrate eukaryotic host cells, thus limiting the efficacy of antibiotics that primarily act within the extracellular space (Alder *et al*, 2020; Yu *et al*, 2020; Cahill *et al*, 2021; Kwon *et al*, 2021). Moreover, the inflammation process may be prolonged, causing delayed recovery and residual joint damage, which affects joint articulation, stability, and range of motion (Howard *et al*, 1976; Welkon *et al*, 1986). Existing research to treat septic arthritis has focused on antibiotic treatment and is lacking with respect to evidence and knowledge of the effects of anti‐inflammatory drugs—administered either alone or adjunctive to standard antibiotic therapy—on septic arthritis‐induced joint damage.

Glycolysis begins with the uptake of extracellular glucose from the extracellular environment and intracellular processing of glucose via glycolysis to produce pyruvate along with numerous metabolites (O'Neill *et al*, 2016). Nonproliferative cells usually metabolize glucose to pyruvate via glycolysis in the presence of oxygen during the process of mitochondrial oxidative phosphorylation (OXPHOS) (Vander Heiden *et al*, 2009). When oxygen is limited, nonproliferative cells can redirect the pyruvate generated by glycolysis to lactate to allow glycolysis to continue by recycling nicotinamide adenine dinucleotide for hydrogen (NADH) back to NAD^+^. Anaerobic glycolysis is a relatively inefficient pathway for the generation of intracellular ATP compared to OXPHOS due to the net production of 2 ATP per single molecule of glucose. Nevertheless, anaerobic glycolysis allows for the maintenance of cell viability in oxygen‐limited environments. Glycolytic upregulation has been reported in other immune cell types in the myeloid and lymphoid lineages in the regulation of intracellular metabolic pathways that respond to extracellular signals to mediate immune function, immune cell differentiation, and effector function (Kornberg, 2020). Following inflammatory activation of myeloid and lymphoid lineage cells, lactate is produced by aerobic and anaerobic glycolysis independent of oxygen availability. Patients with septic shock were found to have significantly higher concentrations of lactate, which was associated with a higher risk of overall mortality (Chertoff *et al*, 2015; Shankar‐Hari *et al*, 2016). Synovial lactate levels were higher in patients with inflammatory arthritis than those with non‐inflammatory arthritides (Gobelet & Gerster, 1984; Mathews *et al*, 2007; Horowitz *et al*, 2011; Nair *et al*, 2017; Pucino *et al*, 2020). The highest lactate levels were detected from the synovial fluid of patients with septic arthritis than noninfectious, inflammatory arthritides, suggesting that synovial lactate levels may demonstrate high sensitivity and specificity for septic arthritis (Lenski & Scherer, 2014; Berthoud *et al*, 2020). Despite this, the relationship between septic arthritis and glycolysis and the role of glycolysis‐targeting drugs have not been evaluated and described. Herein, we establish a clinically relevant murine model in which synovial lactate levels are elevated in the setting of MRSA‐induced septic arthritis and investigate the role and effect of adjunctive glycolysis‐targeting therapy for septic arthritis.

## Results

### MRSA incites host inflammation and upregulation of glycolysis in septic arthritis

**Figure 1:**
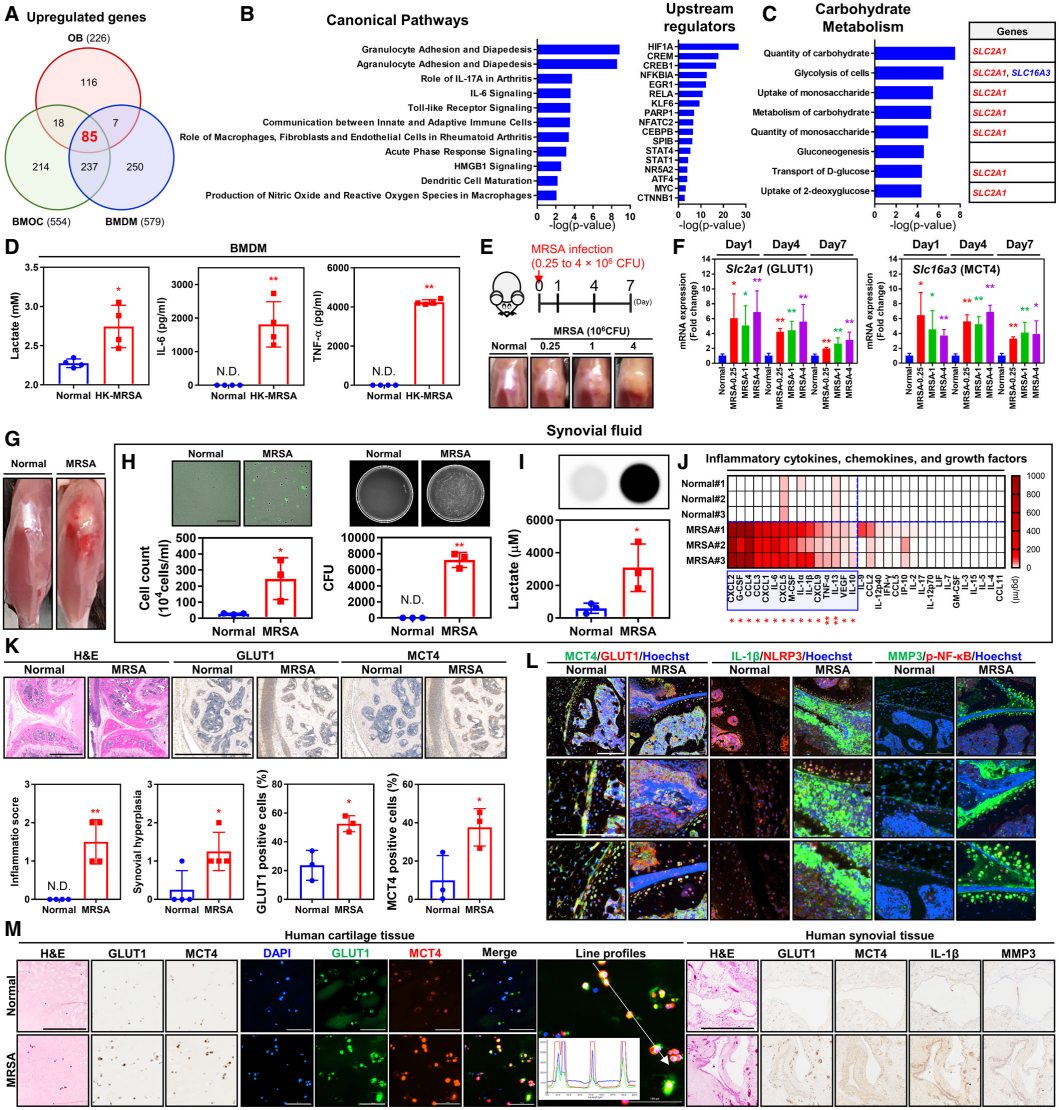
Septic arthritis caused by MRSA infection induces inflammation and glycolysis A–CPrimary osteoblast (OB), bone‐marrow‐derived osteoclast (BMOC), and bone‐marrow‐derived macrophage (BMDM) cells were infected with MRSA, the transcriptome expression profiles of which were analyzed with RNA‐sequencing. The expression of 85 genes increased in the setting of MRSA infection. Subsequently, the canonical pathways, upstream regulators, and corresponding category of carbohydrate metabolism were analyzed and are presented in a histogram. See Appendix Fig S1 for detailed information.DSecretion of lactate, IL‐6, and TNF‐α were measured in BMDM cells infected with heat‐killed MRSA (HK‐MRSA; 4 × 10^6^ CFU) for 24 h.E, FC57BL/6J mice were intraarticularly infected with different concentrations of MRSA (*n* = 4 per group). (E) Physiological changes at 7 days were observed and representative images were captured. (F) mRNA expression levels of *Slc2a1* and *Slc16a1* were measured.G–KC57BL/6J mice were intraarticularly infected with MRSA (4 × 10^6^ CFU; *n* = 3–4 per group) and left untreated for 24 h. (G) Representative images of physiological changes to the knee joint. (H) The amount of infiltrating immune cells in synovial fluid was quantified and detected using MRSA expressive of GFP within immune cells (Scale bar: 100 μm). See EV2 for individual images. MRSA bioburden in synovial fluid was quantified in colony‐forming units (CFU). (I) Lactate level in synovial fluid was measured and the relative luminescence intensity is shown. (J) Intraarticular inflammation including cytokines, chemokines, and growth factors was measured in synovial fluid; blue box indicates factors that changed significantly. See Appendix Fig S2B for individual factor changes. (K) Paraffin‐embedded tissues were sectioned and used to measure inflammation score and synovial hyperplasia. GLUT1 and MCT4 expression was detected, and the percentage of positively staining cells were determined (Scale bar: 1,000 μm).LC57BL/6J mice were intraarticularly infected with MRSA (4 × 10^6^ CFU) and left untreated for 14 days. Paraffin‐embedded tissues were sectioned and used to measure co‐expression and ‐localization levels of MCT4, GLUT1, IL‐1β, NLRP3, MMP3, p‐NF‐κB (Scale bar: 100 μm). See Appendix Fig S2C for individual images.MHuman cartilage and synovial tissue were infected with MRSA (4 × 10^6^ CFU) for 24 h. The co‐expression and localization levels of GLUT1 and MCT4 in human cartilage tissue were measured and confirmed by line profile analysis (Scale bar: 100 μm). Expression of GLUT1, MCT4, IL‐1β, and MMP3 was measured in human synovial tissue (Scale bar: 1,000 μm). Data information: *In vitro* experiments were repeated at least three times with representative results. *In vivo* experiments were repeated in at least two independent experiments, with one or two experiments performed for each group. Error bars show means ± SD with individual data points. Two‐tailed unpaired *t*‐test analysis was conducted to determine statistical significance (*P < 0.05 or **P < 0.01; N.D. = not detected).

We constructed a whole transcriptome data set using RNA sequencing to analyze cellular responses to MRSA infection in bone marrow‐derived macrophages (BMDM), bone marrow‐derived osteoclasts (BMOC), and osteoblasts (OB), which were derived from murine cells. Eighty‐five genes upregulated in the setting of MRSA infection were associated with cellular host defenses such as agranulocyte and granulocyte adhesion and diapedesis, Toll‐like receptor signaling, IL‐6 signaling, and signaling pathways that contribute to arthritic disease (Fig 1A and B; Appendix Fig S1A). Upstream regulatory factor analysis of these genes showed that hypoxia‐inducible factor 1 alpha (HIF‐1α) and nuclear factor κ‐light‐chain‐enhancer of activated B cells (NF‐κB) were major regulators of gene expression and was associated with proinflammatory cytokines, chemokines and glycolysis (Fig 1B; Appendix Fig S1B and C). The expression of genes related to carbohydrate metabolism was significantly augmented with MRSA infection, as expression of the *Slc2a1* gene (also known as GLUT1) increased, so did activity in all metabolic pathways, with the exception of gluconeogenesis (Fig 1C; Appendix Fig S1D). Expression of *Slc2a1* and *Slc16a3* (also known as monocarboxylate transporter 4; MCT4) genes was associated with the upregulation of glycolysis. We observed increases in IL‐6, TNF‐α, and glycolysis‐mediated lactate secretion in heat‐killed MRSA (HK‐MRSA) infected BMDM cells (Fig 1D). We verified increases in lactate secretion via glycolysis in the setting of MRSA‐induced septic arthritis and a dose‐dependent response with inoculated MRSA burden. As the physiological symptoms of septic arthritis worsened with greater MRSA bioburden, the mRNA expression of *Slc2a1* and *Slc16a3* increased, and expression levels slightly decreased over time, but still remained higher than normal (Fig 1 E and F). Joint swelling was observed in our murine models of MRSA‐induced septic arthritis at 1 day, mimicking symptoms of septic arthritis in human patients (Fig 1G). Increases in joint bioburden were verified by the identification of numerous green fluorescent protein (GFP)‐positive cells from synovial fluid, from which a high number of colony‐forming units (CFU) subsequently grew (Fig 1H; Appendix Fig S2A). The accumulation of lactate, proinflammatory cytokines, chemokines, and growth factors in synovial fluid increased with greater MRSA bioburden (Fig 1I and J; Appendix Fig S2B). Histopathologic examination, including inflammation score and synovial hyperplasia, revealed increases in the percentage of cells that stained positive for GLUT1 and MCT4 and mirrored symptomatic progression of septic arthritis in MRSA‐infected mice (Fig 1K). The co‐expression and localization levels of MCT4, GLUT1, IL‐1β, NLRP3, MMP3, and p‐NF‐κB increased by the 14‐day timepoint in chondrocytes and immune cells in our *in vivo* model of septic arthritis (Fig 1L; Appendix Fig S2C). The expression of GLUT1 and MCT4 increased in the setting of MRSA infection and was co‐expressed and localized to infected chondrocytes in human cartilage tissue (Fig 1M). MRSA infection also yielded increases in GLUT1, MCT4, interleukin‐1β (IL‐1β), and matrix metallopeptidase 3 (MMP3) expression in human synovial tissues (Fig 1M). We hypothesized that inflammatory responses and upregulation of glycolysis seen in septic arthritis would be proportional to cartilage disruption in inflammatory arthritides such as rheumatoid arthritis. From single‐cell RNA‐sequencing analysis of synovial tissues isolated from rheumatoid arthritis patients, we identified that the expression of *SLC2A1* and *SLC16A3* increased along with concentrations of proinflammatory factors and glycolytic enzymes (Appendix Fig S3). Taken together, we confirmed that MRSA‐induced septic arthritis markedly increased intraarticular inflammation and that the expression of GLUT1 and MCT4, factors crucial to glycolysis, also increased in the setting of infection, thus augmenting lactate accumulation within synovial fluid. These results suggest that glycolysis can be an important key factor in inflammatory joint damage following infection and autoimmune diseases.

### Intraarticular inflammation and increases in glycolysis persist despite antibiotic treatment of septic arthritis

**Figure 2:**
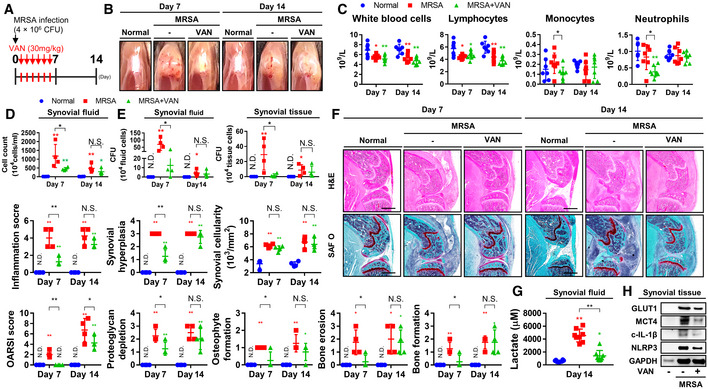
MRSA‐induced septic arthritis incites intraarticular inflammation and lactate production that persists after vancomycin treatment AC57BL/6J mice were subcutaneously treated with vancomycin (30 mg/kg) for 6 days following MRSA (4 × 10^6^ CFU) infection and sacrificed at 7 and 14 days (*n* = 4–8 per group).BPhysiological changes were identified and representative images were generated.CBlood was collected and complete blood counts (CBC) were measured.D, EInfiltrating immune cell counts within synovial fluid and MRSA synovial fluid bioburden were quantified. MRSA bioburden in synovial tissue was quantified.FParaffin‐embedded tissues were sectioned and measured at 7 and 14 days with respect to inflammation score, synovial hyperplasia, synovial cellularity, OARSI score, proteoglycan depletion, osteophyte formation, bone erosion, and bone formation (Scale bar: 1,000 μm).G, HLactate level in synovial fluid was measured at 14 days, and expression of GLUT1, MCT4, cleaved‐IL‐1β, and NLRP3 in synovial tissue was measured with GAPDH as a loading control. Data information: *In vivo* experiments were repeated in at least two independent experiments. Error bars show means ± SD with individual data points. One‐way ANOVA with Tukey's *post hoc* analysis was conducted to determine statistical significance (*P < 0.05 or **P < 0.01; N.D. = not detected; N.S. = not significant). Source data are available online for this figure.

To verify that the activation of glycolysis in MRSA‐induced septic arthritis conditions can be managed with conventional antibiotic treatment, vancomycin was systemically administered for six consecutive days after MRSA inoculation into the knee joint in accordance with clinical guidelines (Liu *et al*, 2011; Fig 2A). Vancomycin treatment prevented the development of symptoms characteristic of septic arthritis such as knee edema within the first 7 days of infection, though these symptoms worsened by day 14 (Fig 2B). Leukopenia was observed at both time points, while neutropenia was observed to occur on day 7, but had normalized by day 14 (Fig 2C). The number of infiltrating immune cells and MRSA CFU counts isolated from synovial fluid and tissue significantly decreased after 1 week of vancomycin treatment, though the difference between vancomycin‐treated and untreated groups was not statistically significant by day 14 (Fig 2D and E). Inflammation score, synovial hyperplasia, synovial cellularity, OARSI score, proteoglycan depletion, osteophyte formation, bone erosion, and bone formation were all observed to increase with MRSA infection and subsequently decreased within 7 days with vancomycin treatment, though most scores worsened by day 14 (Fig 2F). The increase in the number of cells that stained positive for GLUT1 observed with MRSA infection was significantly reduced by, but did not normalize with, vancomycin treatment (Appendix Fig S4). A similar trend was observed with respect to synovial lactate accumulation, which increased in the setting of MRSA infection and was reduced but not fully normalized by vancomycin treatment (Fig 2G). The expression of GLUT1, MCT4, NLR family pyrin domain containing 3 (NLRP3), and mature interleukin‐1β (IL‐1β) increased with MRSA infection, but did not fully normalize with vancomycin treatment (Fig 2H). Our results demonstrate that even following conventional antibiotic treatment, glycolytic processes remained activated, alongside proinflammatory processes destructive to cartilage and bone, although the phenotypic symptoms of septic arthritis were relieved.

**Figure EV1:**
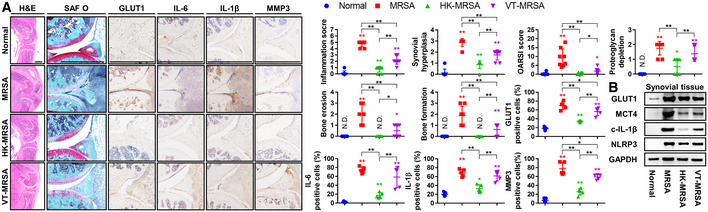
Inactive or antibiotic‐treated MRSA induces inflammatory arthritis along with glycolysis C57BL/6J mice were subcutaneously infected with MRSA (4 × 10^6^ CFU), heat‐killed MRSA (HK‐MRSA), and vancomycin‐treated MRSA (VT‐MRSA) and sacrificed at 7 days (*n* = 8 per group). Paraffin‐embedded tissues were sectioned and measured with respect to inflammation score, synovial hyperplasia, OARSI score, proteoglycan depletion, bone erosion, and bone formation (Scale bar: 1,000 μm). Expression of GLUT1, IL‐6, IL‐1β, and MMP3 was detected, and the percentages of positively staining cells were determined.Expression of GLUT1, MCT4, cleaved‐IL‐1β, and NLRP3 in synovial tissue was measured with GAPDH as a loading control. Data information: *In vivo* experiments were repeated in at least two independent experiments. Error bars show means ± SD with individual data points. One‐way ANOVA with Tukey's *post hoc* analysis was conducted to determine statistical significance (*P < 0.05 or **P < 0.01; N.D. = not detected; N.S. = not significant). Source data are available online for this figure.

We hypothesized that the production of inflammatory factors and activation of glycolysis by immune responses to either inactivated or persistent live MRSA despite conventional antibiotic treatment promulgates the development of inflammatory arthritis. We thus constructed a murine knee joint model infected with HK‐MRSA or vancomycin‐treated MRSA (VT‐MRSA), in which increased inflammation scores, synovial hyperplasia, and proteoglycan depletion were observed (Fig EV1A). OARSI scores, bone erosion, and bone formation developed in VT‐MRSA to similar extents as observed in live MRSA‐infected joints, though lower degrees of inflammation and bone damage were observed in HK‐MRSA‐infected mice. The number of immune cells, chondrocytes, and synoviocytes that stained positive for GLUT1, IL‐6, IL‐1β, MMP3—factors associated with glycolysis, inflammation, and cartilage degradation—were elevated following inoculation of HK‐MRSA and VT‐MRSA. Similarly, the expression of GLUT1, MCT4, NLRP3, and the mature form of IL‐1β increased in the setting of MRSA, HK‐MRSA, and VT‐MRSA infection (Fig EV1B). We confirmed that increased inflammation and glycolysis persisted within the joint space, thereby disrupting articular cartilage and bone, following conventional antibiotic treatment. These results support the necessity of identifying and developing adjuvant therapies that target glycolysis and that can be administered alongside antibiotics to protect against host tissue destruction of articular cartilage and bone.

**Figure 3:**
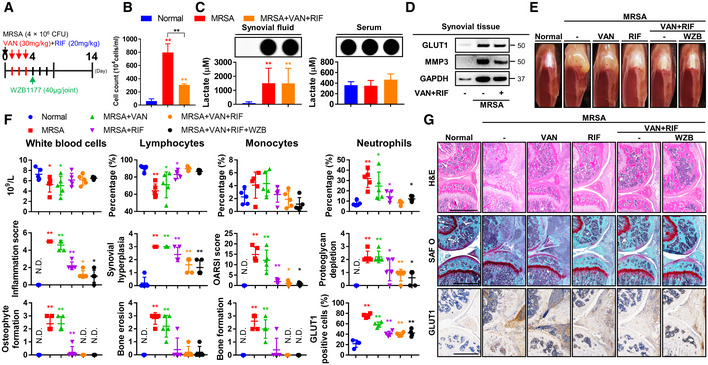
Combined antibiotic treatment of septic arthritis results in sustained intraarticular lactate production not significantly reduced by adjuvant glucose uptake inhibitor treatment C57BL/6J mice were subcutaneously treated with vancomycin (30 mg/kg) and rifampin (20 mg/kg) for 3 days following MRSA (4 × 10^6^ CFU) infection and sacrificed at 4 days (*n* = 3 per group). C57BL/6J mice were subcutaneously treated with vancomycin (30 mg/kg) and/or rifampin (20 mg/kg) for 3 days following MRSA (4 × 10^6^ CFU) infection (*n* = 5 per group). After 1 day, WZB1177 (40 μg/joint), a GLUT1 inhibitor, was intraarticularly injected and sacrificed at 14 days.Infiltrating immune cell counts within synovial fluid were quantified.Serum and synovial fluid lactate levels were measured and the relative luminescence intensity is shown.Expression of GLUT1 and MMP3 in synovial tissue was measured using GAPDH as a loading control.Physiological changes were identified, and representative images were generated.Blood was collected, and complete blood counts (CBC) were measured.Paraffin‐embedded tissues were sectioned and measured with respect to inflammation score, synovial hyperplasia, OARSI score, proteoglycan depletion, osteophyte formation, bone erosion, and bone formation (Scale bar: 1,000 μm). The expression of GLUT1 was detected, and the percentage of positively staining cells was determined. Data information: *In vivo* experiments were repeated in at least two independent experiments. Error bars show means ± SD with individual data points. One‐way ANOVA with Tukey's *post hoc* analysis was conducted to determine statistical significance (*P < 0.05 or **P < 0.01; N.D. = not detected). Source data are available online for this figure.

### Combined systemic antibiotic treatment improves, but adjuvant treatment with a glucose uptake inhibitor demonstrates no additional benefit in, pathologic sequelae of septic arthritis

We identified a heavy intracellular MRSA bioburden within infiltrating cells derived from synovial fluid and confirmed that vancomycin treatment alone was insufficient to eradicate intracellular MRSA infection in the setting of septic arthritis. Similarly, we demonstrated that MRSA can achieve intracellular penetration, which reduces the efficacy of conventional therapeutic antibiotic regimens involving vancomycin, which acts solely within the extracellular compartment (Alder *et al*, 2020; Yu *et al*, 2020; Cahill *et al*, 2021; Kwon *et al*, 2021). The limitation of vancomycin can be improved by combining it with rifampin, which can penetrate cells to act upon intracellular reservoirs of MRSA in bone and soft tissues (Kwon *et al*, 2021, 2022). The combined antibiotic treatment (i.e., vancomycin and rifampin) model described herein was evaluated in the setting of septic arthritis in conjunction with activation of glycolytic pathways (Fig 3A). The number of infiltrating immune cells in synovial fluid was reduced, though the accumulation of lactate within MRSA‐infected synovial fluid and expression of GLUT1 and MMP3 in MRSA‐infected synovial tissue were not significantly reduced, by combined antibiotic therapy (Fig 3B–D). Interestingly, serum lactate levels did not change markedly in the setting of antibiotic‐treated or untreated MRSA infection, suggesting that synovial lactate is produced and accumulates independently of systemic lactate and persists after combined antibiotic therapy.

**Figure EV2:**
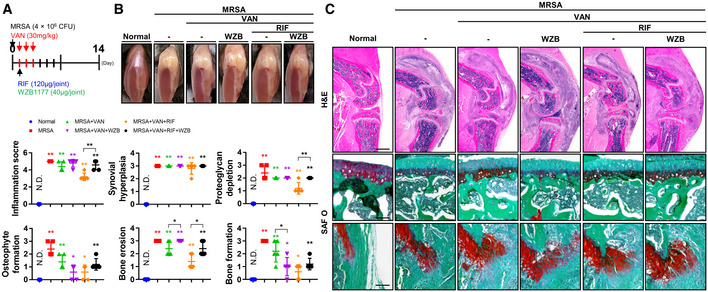
Adjuvant glucose uptake inhibitor treatment exacerbates the effects of combined antibiotic treatment in septic arthritis C57BL/6J mice were subcutaneously treated with vancomycin (30 mg/kg) for 3 days following MRSA (4 × 10^6^ CFU) infection. After MRSA infection, rifampin (120 μg/joint) and/or WZB1177 (40 μg/joint) was intraarticularly injected and sacrificed at 14 days (*n* = 5 per group).Physiological changes were observed, and representative images were generated.Paraffin‐embedded tissues were sectioned and measured with respect to inflammation score, synovial hyperplasia, proteoglycan depletion, osteophyte formation, bone erosion, and bone formation (Scale bar: 1,000 or 100 μm). Data information: *In vivo* experiments were repeated in at least two independent experiments. Error bars show means ± SD with individual data points. One‐way ANOVA with Tukey's *post hoc* analysis was conducted to determine statistical significance (*P < 0.05 or **P < 0.01; N.D. = not detected).

To investigate the function of GLUT1 in septic arthritis, we designed a model in which WZB117, a selective irreversible inhibitor of GLUT1 (Liu *et al*, 2012), was injected into the knee joint after combined antibiotic treatment (Fig 3A). Greater symptomatic improvement of MRSA infection was observed with rifampin treatment alone relative to vancomycin treatment, with the greatest improvement observed in mice that received combinatorial therapy (Fig 3E). Leukopenia caused by MRSA infection was improved with combined antibiotic treatment, while infection‐incited neutrophilia was reduced with combinatorial antibiotic therapy (Fig 3F). Similar trends were observed with respect to the percentage of GLUT1‐staining cells, inflammation score, synovial hyperplasia, OARSI score, proteoglycan depletion, osteophyte formation, bone erosion, and bone formation after combined antibiotic treatment relative to vancomycin or rifampin monotherapy (Fig 3G). However, inhibition of GLUT1 by WZB117 failed to demonstrate a statistically significant effect on physiological and histopathological symptoms of septic arthritis when administered adjunctively to the combined antibiotic regimen. We subsequently constructed a model in which rifampin was injected into the knee joint 24 h after MRSA infection, followed by systemic combined antibiotic treatment (Fig EV2A). Rifampin monotherapy prevented the development of physiological and histopathological symptoms characteristic of septic arthritis such as knee edema and inflammation score than the vancomycin monotherapy condition (Fig EV2B and C). When WZB117 was injected into the knee joint with and without rifampin, GLUT1 inhibition exacerbated or failed to significantly improve the physiological and histopathological symptoms of septic arthritis compared to the combined antibiotic treatment condition alone. We thus confirmed that even though symptoms of septic arthritis may improve with combinatorial antibiotic treatment, intraarticular glycolysis‐mediated lactate production persisted, and adjuvant GLUT1 inhibition failed to ameliorate signs of disease progression.

**Figure 4:**
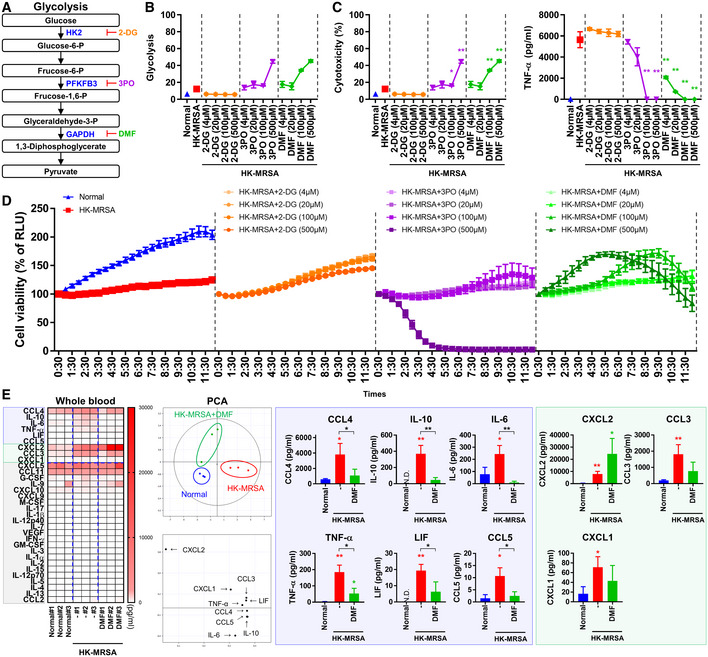
Inhibition of glycolysis with DMF normalizes the production of proinflammatory markers ASchematic of therapeutic targets: 2‐DG targets HK2, 3PO targets PFKFB3, and DMF targets GAPDH within the glycolysis pathway.B–DRAW264.7 cells were treated with 2‐DG, 3PO, and DMF at concentrations of 4–500 μM for 1 h and then infected with HK‐MRSA (4 × 10^6^ CFU) for 12 h. (B, C) Cytotoxicity was measured as a function of lactate dehydrogenase (LDH) level in the supernatant medium and TNF‐α levels in the supernatant was measured. (D) Cell viability was measured in real‐time at 30‐min intervals for total of 12 h.EBlood was collected and treated with DMF (150 μg/ml) for 1 h and infected with HK‐MRSA (4 × 10^6^ CFU) for 6 h. Inflammatory cytokine, chemokine, and growth factors were measured and analyzed by principal component analysis (PCA). The blue and green boxes highlight factors that underwent significant expression changes in the setting of infection. The blue box represents factors that were significantly reduced due to DMF, and the green box represents factors that operated independently of DMF. Data information: *In vitro* experiments were repeated at least three times with representative results. Error bars show means ± SD with individual data points. Two‐tailed unpaired *t*‐test analysis and one‐way ANOVA with Tukey's *post hoc* analysis were conducted to determine statistical significance (*P < 0.05 or **P < 0.01; N.D. = not detected).

### Inhibition of glycolysis with DMF alleviates inflammation via inhibition of downstream NF‐κB and ERK signaling pathways

Since glucose uptake is crucial to immune responses to septic arthritis, we sought to selectively evaluate the separate effects of glycolysis pathway modulation compared to regulation of cellular glucose uptake. To investigate the function of the glycolysis pathway, we selected 2‐deoxyglucose (2‐DG) targeting hexokinase 2 (HK2), 3‐(3‐pyridinyl)‐1‐(4‐pyridinyl)‐2‐propen‐1‐one 1 (3PO) targeting 6‐phosphofructo‐2‐kinase/fructose‐2,6‐biphosphatase 3 (PFKFB3), and GAPDH as molecular targets based on previous studies (O'Neill *et al*, 2016; Kornberg *et al*, 2018; Palsson‐McDermott & O'Neill, 2020) and examined the effect of target inhibition in murine macrophage cells infected with HK‐MRSA (Fig 4A). Treatment with 2‐DG ranging from 4–500 μM did not significantly alter cytotoxicity and TNF‐α production in HK‐MRSA‐infected murine macrophages (Fig 4B and C). DMF treatment significantly reduced TNF‐α production without cytotoxic effects at concentrations < 20 μM, though this did not meaningfully change with 3PO treatment. At concentrations > 100 μM, TNF‐α production normalized with 3PO and DMF treatment, though cytotoxic effects were observed. We determined that 2‐DG treatment did not markedly affect cell viability, while 3PO treatment significantly reduced cell viability at a concentration of 500 μM (Fig 4D). Treatment with DMF slightly decreased cell viability at concentrations higher than 100 μM, though to a lesser extent than did 3PO treatment. Due to the FDA approval status of DMF for the treatment of patients with multiple sclerosis and its anti‐inflammatory effects coupled with mild to no cytotoxic effects, DMF was chosen as the glycolysis‐disrupting agent in our evaluation of the function of glycolysis in MRSA‐induced septic arthritis.

**Figure EV3:**
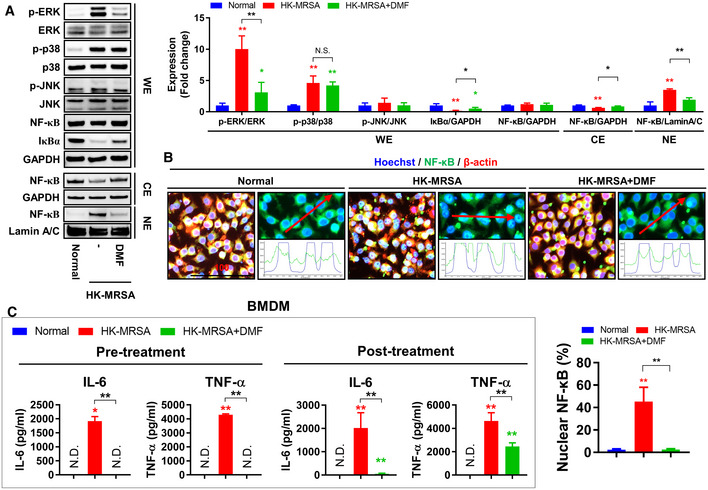
DMF normalizes the generation of inflammation mediated by NF‐κB and ERK signaling pathways A, BRAW264.7 cells were treated with DMF (20 μM) for 1 h and then infected with HK‐MRSA (4 × 10^6^ CFU) for 30 min. (A) Expression of p‐ERK, ERK, p‐p38, p38, p‐JNK, JNK, NF‐κB, and IκBα in the whole protein extraction (WE) and expression of NF‐κB in both cytoplasmic protein extraction (CE) and nuclear protein extraction (NE) were measured; GAPDH was used as a loading control for WE and CE, and Lamin A/C was used as a loading control for NE. (B) The translocation of NF‐κB into the nucleus was measured and analyzed; β‐actin was used for cell staining and Hoechst was used for nuclear staining (Scale bar: 100 μm). See Appendix Fig S5 for individual images.CBMDM cells were treated with DMF (50 μg/ml) for 1 h and infected with HK‐MRSA (4 × 10^6^ CFU) for 24 h, which we termed DMF pretreatment. BMDM cells were infected with HK‐MRSA (4 × 10^6^ CFU) for 1 h and treated with DMF (50 μg/ml) for 24 h, which we termed DMF posttreatment. Production of IL‐6 and TNF‐α were measured. Data information: *In vitro* experiments were repeated at least three times with representative results. Error bars show means ± SD with individual data points. Two‐tailed unpaired *t*‐test analysis and one‐way ANOVA with Tukey's *post hoc* analysis were conducted to determine statistical significance (*P < 0.05 or **P < 0.01; N.D. = not detected). Source data are available online for this figure.

NF‐κB and mitogen‐activated protein kinase (MAPK) signaling pathways are important for proinflammatory cytokine production and were found to be activated in HK‐MRSA‐infected macrophages, with the exception of the c‐JUN N‐terminal kinase (JNK) signaling pathway (Fig EV3A and B; Appendix Fig S5). DMF treatment inhibited signaling through NF‐κB and extracellular signal‐regulated kinase (ERK), though no significant changes in p38 signaling pathway activity were observed. The production of IL‐6 and TNF‐α, which increased in BMDM cells infected with HK‐MRSA, was inhibited with DMF pretreatment, as shown by our previous results (Fig EV3C). DMF treatment after infection with HK‐MRSA, which is termed posttreatment, also suppressed the production of IL‐6 and TNF‐α. The production of proinflammatory cytokines such as IL‐6, TNF‐α, and IL‐10, chemokines such as CCL4 and CCL5, and anti‐inflammatory cytokines such as IL‐10 and leukemia inhibitory factor (LIF) in the bloodstream of HK‐MRSA‐infected mice was increased and subsequently normalized by DMF treatment (Fig 4E). Production of chemokines such as CXCL1, CXCL2, and CCL3, which mediate the recruitment of neutrophils and monocytes, in the bloodstream of HK‐MRSA‐infected mice, was independent of DMF treatment. These results suggest that glycolysis plays an important role in the production of proinflammatory cytokines and chemokines as part of the innate immune response to MRSA infection. We confirmed that DMF targeting of GAPDH, an enzyme important for glycolysis, normalized the production of proinflammatory cytokines and chemokines through inhibition of the NF‐κB and ERK signaling pathways. Our results suggest that the anti‐inflammatory effects of DMF have the potential to protect against cartilage and bone disruption mediated by persistent inflammation.

**Figure 5:**
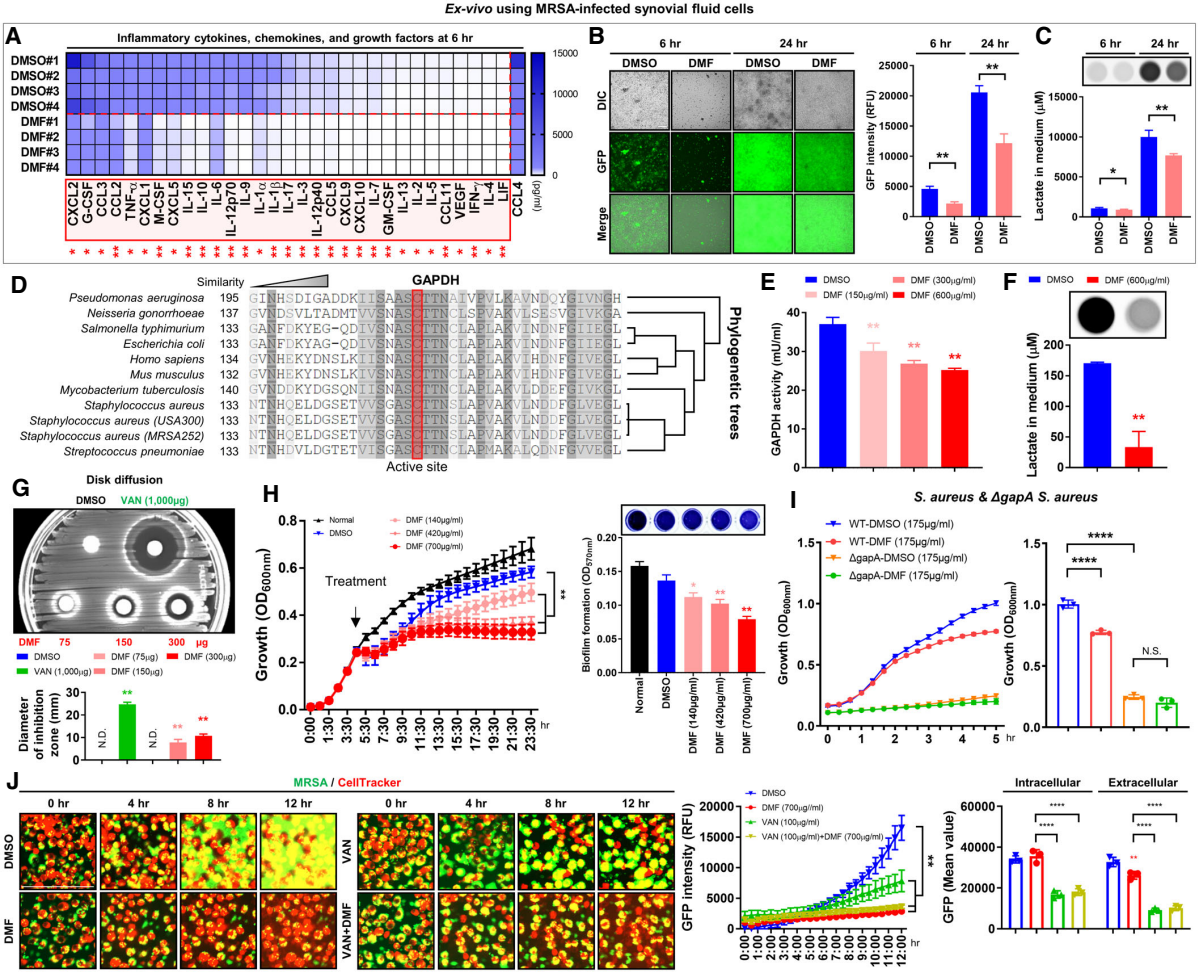
DMF suppresses MRSA growth and biofilm formation via inhibition of glycolysis A–CC57BL/6J mice were intraarticularly infected with MRSA (4 × 10^6^ CFU; *n* = 4–5 per group) for 24 h. Synovial fluid cells were collected and (5 × 10^5^) seeded on antibiotic‐free DMEM medium with or without DMF (300 μg/ml) for 6 and 24 h. (A) Inflammatory cytokine, chemokine, and growth factors in the supernatant medium at 6 h were measured. The red box highlights factors that underwent significant expression change in the setting of DMF treatment. (B) MRSA expressive of green fluorescent protein (GFP) was detected and measured (Scale bar: 100 μm). (C) Lactate levels in the supernatant medium were measured.DAlignment of an amino acid sequence of GAPDH in *Homo sapiens*, *Mus musculus*, *Pseudomonas aeruginosa*, *Neisseria gonorrhoeae*, *Salmonella typhimurium*, *Escherichia coli*, *Mycobacterium tuberculosis*, *Staphylococcus aureus*, *Staphylococcus aureus* (USA300), *Staphylococcus aureus* (MRSA252), and *Streptococcus pneumoniae*; the red box indicates the active site of cysteine. A full amino acid sequence of the phylogenetic tree is in Appendix Fig S6.EWhole protein extracted from MRSA was treated with DMF (150, 300, and 600 μg/ml) for 1 h and GAPDH activity was subsequently measured.FMRSA (4 × 10^6^ CFU) was cultured in LB broth with or without DMF (600 μg/ml) for 1 h and lactate secretion was measured from LB broth.GInhibition zones in disk fusion assays using vancomycin (1,000 μg) and DMF (75–300 μg) were measured and presented in a histogram.HMRSA (4 × 10^6^ CFU) was cultured in LB broth for 4 h and treated with DMF (140, 420, and 700 μg/ml), after which absorbance was measured at 1‐h intervals for 20 h total. Biofilm formation was measured for 24 h, with color changes shown with representative images.I
*Staphylococcus aureus* (RN4220) and ΔgapA *Staphylococcus aureus* (MRSA252; 4 × 10^6^ CFU) were cultured in TSB broth with or without DMF (175 μg/ml) and absorbance was measured at 20‐min intervals for 5 h; DMSO was used as the negative control.JRAW264.7 cells were stained with CellTracker and infected with MRSA (4 × 10^6^ CFU) for 2 h. The cells were then washed to remove extracellular MRSA. Medium containing DMF (700 μg/ml), vancomycin (100 μg/ml), and DMF (700 μg/ml) with vancomycin (100 μg/ml) was added and GFP intensity was measured at 1‐h intervals for a total of 12 h. Representative images of differential fluorescence with CellTracker and GFP at 4‐h intervals for a total of 12 h. See Movie EV2 for individual time points. Intracellular and extracellular MRSA were semi‐quantitatively measured at 12 h. Data information: *In vitro* experiments were repeated at least three times with representative results. *Ex vivo* experiments were repeated twice. Error bars show means ± SD with individual data points. Two‐tailed unpaired *t*‐test analysis was conducted to determine statistical significance (*P < 0.05 or **P < 0.01 or ***P < 0.001 or ****P < 0.0001; N.D. = not detected; N.S. = not significant).

### DMF suppresses extra‐ and intracellular MRSA growth and biofilm formation

**Figure EV4:**
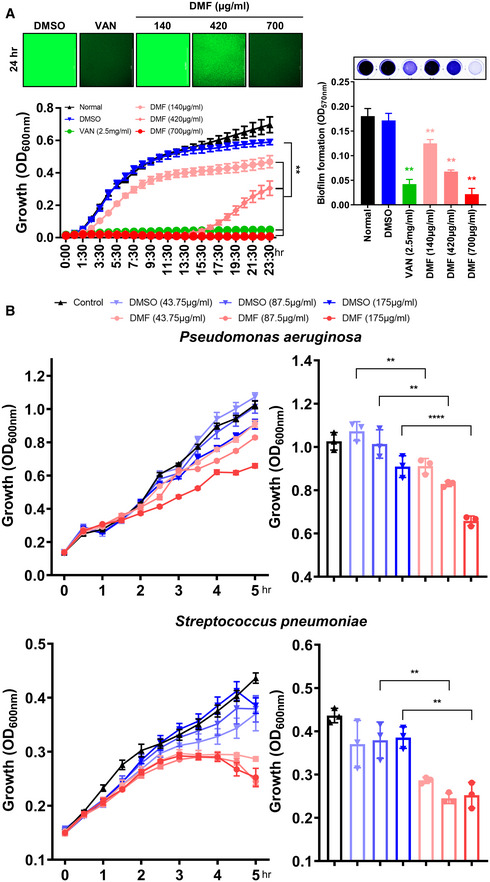
DMF inhibits the growth of numerous types of bacteria MRSA (4 × 10^6^ CFU) was cultured in LB broth with or without DMF (140, 420, and 700 μg/ml). Absorbance and GFP intensity were measured at 1‐h intervals for 24 h; vancomycin (2.5 mg/ml) was used as the positive control and DMSO as the negative control. Images depict MRSA expressive of green fluorescent protein (GFP) (Scale bar: 1,000 μm). See Movie EV1 for MRSA expressing GFP at individual time‐points. Biofilm formation was measured for 24 h, with color changes shown in representative images.
*Pseudomonas aeruginosa* (PA01) was cultured in TSB broth, and *Streptococcus pneumoniae* (TIGER4) was cultured in THB broth with or without DMF (43.75, 87.5, and 175 μg/ml). Absorbance was measured at 20‐min intervals for 5 h with DMSO as the negative control. Data information: *In vitro* experiments were repeated at least three times with representative results. *Ex vivo* experiments were repeated twice. Error bars show means ± SD with individual data points. Two‐tailed unpaired *t*‐test analysis was conducted to determine statistical significance (*P < 0.05 or **P < 0.01 or ***P < 0.001 or ****P < 0.0001; N.D. = not detected; N.S. = not significant).

To verify the anti‐inflammatory effect of DMF in septic arthritis, synovial fluid was isolated from infected mice and treated with DMF in an antibiotic‐free medium. Similar to previous results, DMF treatment significantly normalized the production of proinflammatory cytokines, chemokines, and growth factors from infiltrating immune cells within synovial fluid (Fig 5A). Interestingly, treatment with DMF inhibited the growth of MRSA in synovial fluid and decreased the level of lactate secretion into the medium (Fig 5B and C). Thus, we hypothesized that DMF can inhibit bacterial growth by directly targeting GAPDH in MRSA cells. Sequence homology analysis revealed that the catalytic cysteine within the active site of GAPDH, which is directly inhibited by DMF, is similar in sequence homology between various types of bacterial species and strains, including *S. aureus* and MRSA (Fig 5D; Appendix Fig S6). We confirmed that DMF directly inhibited GAPDH activity and reduced the amount of lactate secreted via glycolysis produced in the setting of MRSA infection (Fig 5E and F). Antibiotic susceptibility tests involving DMF using disk diffusion showed that the inhibition zone of MRSA increased in a dose‐dependent manner (Fig 5G). We thus postulated that DMF targets GAPDH, impeding glycolysis metabolism, thereby inhibiting MRSA growth. MRSA growth was arrested in a dose‐dependent manner with DMF treatment, which also inhibited biofilm formation (Fig EV4A; Movie EV1). DMF has a bacteriostatic effect on MRSA, as evidenced by its reduction of MRSA growth and biofilm formation (Fig 5H). In addition, we demonstrated that the growth of different types of bacterial species such as *Pseudomonas aeruginosa* and *Streptococcus pneumoniae* was also arrested in a dose‐dependent manner with DMF treatment (Fig EV4B). We confirmed that the growth of GapA mutant (ΔgapA) *S. aureus* was lower than that of wild‐type *S. aureus*, and that DMF was not effective in inhibiting the growth of ΔgapA *S. aureus*, thereby verifying that DMF targets GAPDH (Fig 5I). Even after extensive antibiotic therapy, septic arthritis is characterized by high rates of recurrence due to the internalization of *S. aureus* by host cells, allowing for indolent infection and antibiotic resistance (Hunter *et al*, 2015; Brandt *et al*, 2018; Moldovan & Fraunholz, 2019; Alder *et al*, 2020). Based on this evidence of DMF's effect on mammalian cells and bacteria, we hypothesized that DMF can enter eukaryotic cells to exert its effects within the intracellular environment. We confirmed that vancomycin was effective against extracellular MRSA, but not intracellular MRSA through evidence due to the continued proliferation of intracellular MRSA (Fig 5J; Movie EV2). By contrast, DMF protected against MRSA regrowth by preventing the proliferation and escape of MRSA from the intracellular compartment of activated macrophages independent of vancomycin treatment. For the first time, we demonstrate that DMF leads to bacteriostatic effects on MRSA by directly targeting GAPDH, thereby inhibiting MRSA proliferation and biofilm formation, with potential adjunctive utility in the treatment of septic arthritis.

**Figure 6:**
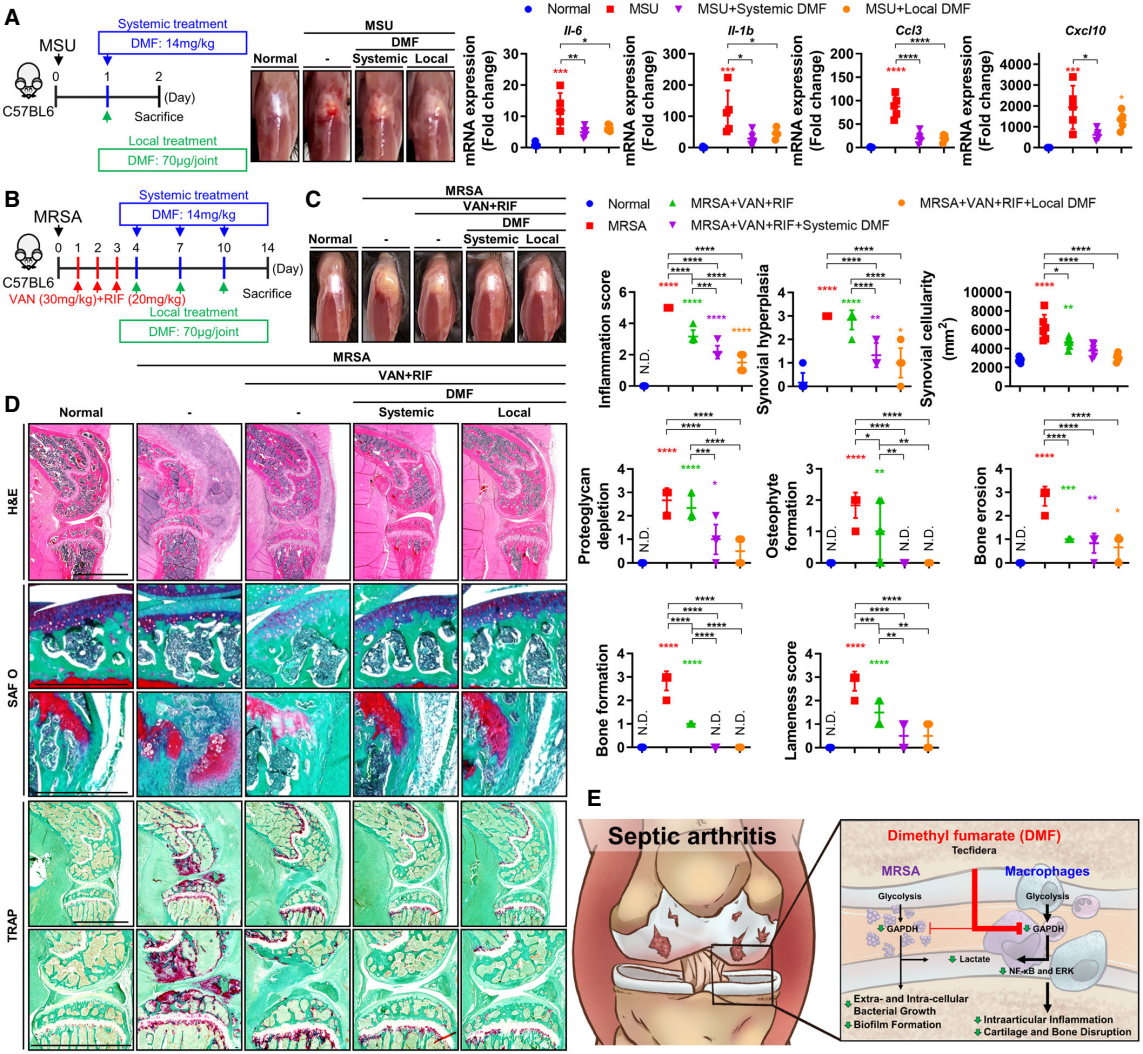
Adjuvant DMF treatment with antibiotic treatment improves the prognosis of MRSA‐induced septic arthritis AC57BL/6J mice were treated with systemic (14 mg/kg) or local (70 μg/joint) DMF treatment following MSU (300 μg/joint) injection (*n* = 5 per group). Observed physiological changes were identified and compared between groups. mRNA expression levels of *Il‐6*, *Il‐1b*, *Ccl3*, and *Cxcl10* were measured.B–DC57BL/6J mice were subcutaneously treated with vancomycin (30 mg/kg) and rifampin (20 mg/kg) for 3 days following MRSA (8 × 10^6^ CFU) infection, followed by systemic (14 mg/kg) or local (70 μg/joint) DMF treatment at 3 days interval for a total of 3 times (*n* = 6 per group). (C) Observed physiological changes were identified and compared between groups. (D) Paraffin‐embedded tissues were sectioned and measured with respect to inflammation score, synovial hyperplasia, synovial cellularity, proteoglycan depletion, osteophyte formation, bone erosion, and bone formation (Scale bar: 2,000 or 500 μm). Bone resorption by osteoclasts was measured by tartrate‐resistant acid phosphatase (TRAP) staining (Scale bar: 2,000 μm). Lameness score was also measured.EIllustrated summary of DMF's effect on MRSA‐induced septic arthritis. Data information: *In vivo* experiments were repeated in at least two independent experiments. Error bars show means ± SD with individual data points. One‐way ANOVA with Tukey's *post hoc* analysis was conducted to determine statistical significance (*P < 0.05 or **P < 0.01 or ***P < 0.001 or ****P < 0.0001; N.D. = not detected).

### Adjuvant DMF treatment alleviates tissue damage in septic arthritis by reducing joint inflammation and articular cartilage and bone destruction

**Figure EV5:**
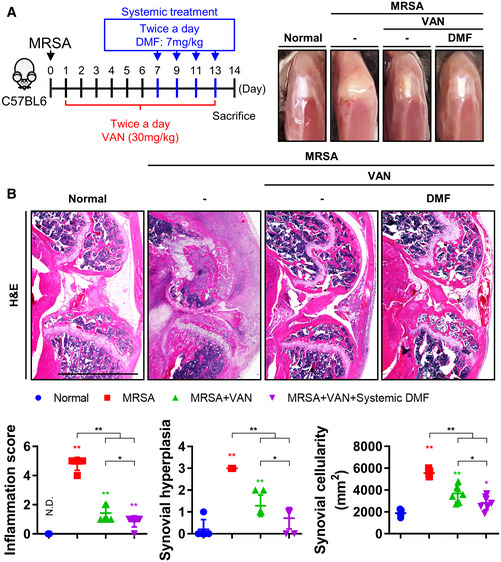
Adjuvant DMF treatment with vancomycin treatment improves the prognosis of MRSA‐induced septic arthritis C57BL/6J mice were subcutaneously treated with vancomycin (30 mg/kg) twice daily for 13 days following MRSA (8 × 10^6^ CFU) infection (*n* = 5–7 per group). At day 7, DMF (7 mg/kg) was subcutaneously administered twice daily at 2‐day intervals for a total of 4 times.
Observed physiological changes were identified and compared between groups.Paraffin‐embedded tissues were sectioned and measured with respect to inflammation score, synovial hyperplasia, and synovial cellularity (Scale bar: 2,000 μm). Data information: *In vivo* experiments were repeated twice per group. Error bars show means ± SD with individual data points. One‐way ANOVA with Tukey's *post hoc* analysis was conducted to determine statistical significance (*P < 0.05 or **P < 0.01; N.D. = not detected).

Since multiple sclerosis patients who receive DMF treatment 2–3 times a day have not been reported to exhibit any notable side effects until 4 weeks of treatment (Fox *et al*, 2012), we sought to evaluate short‐term—1 week—DMF treatment. First, we systemically or intraarticularly administered DMF after intraarticular injection with monosodium urate (MSU), which yielded diminished physiological symptoms of septic arthritis such as knee edema (Fig 6A). We verified that MSU inoculation increased the expression of proinflammatory markers and that these increases were subsequently alleviated by both methods of DMF treatment, lending further credence to the anti‐inflammation effects of DMF. Next, we systemically or intraarticularly administered DMF in 3‐day intervals for a total of three times after combined antibiotic treatment (i.e., vancomycin and rifampin) was systemically administered for 3 days (Fig 6B). Both methods of DMF treatment reduced physiological symptoms such as knee edema in the setting of MRSA infection to a greater extent than combined antibiotic therapy alone (Fig 6C). DMF treatment considerably improved all pathophysiological indicators of disease progression, including lameness score, inflammation score, synovial cellularity, synovial hyperplasia, proteoglycan depletion, osteophyte formation, bone erosion, and bone formation more than combined antibiotic treatment alone (Fig 6D). A large number of osteoclasts (OC), as evidenced by tartrate‐resistant acid phosphatase (TRAP)‐positively staining cells, were observed in the damaged subchondral trabecular bone of the MRSA‐infected joint, yielding decreased trabecular bone epiphyseal bone quality. OCs were also identified in cancellous bone and growth plates and were associated with local reductions in cancellous trabecular bone number and thickness. Combined antibiotic treatment reduced the total number of OC present, though they were still observed in trabecular bone within the epiphysis, cancellous bone, and growth plates of infected, treated mice, as well as in articular cartilage. Both methods of DMF treatment reduced OC counts in epiphyseal trabecular bone and cancellous bone to a greater extent than combined antibiotic treatment alone, suggesting that DMF can protect against OC‐mediated cartilage and bone damage. Similarly, we identify that the expression of numerous inflammation‐associated parameters were reduced following treatment with twice daily systemic vancomycin administered over 13 days in conjunction with twice daily systemic DMF treatment every other day for 7 days (Fig EV5).

## Discussion

Therapeutic drug repurposing constitutes a useful and economical method of biomedical inquiry by reducing the time and cost associated with new drug development (Pushpakom *et al*, 2019). In this study, we provide evidence that adjunctive, repurposed administration of DMF in conjunction with systemic antibiotics alleviated symptoms of MRSA‐induced septic arthritis by protecting against articular cartilage and bone disruption that persists even after antibiotic treatment and further reducing MRSA bioburden (Fig 6E).

Metabolic reprogramming occurred in response to changes in nutrient or oxygen availability and is regulated by pathogen‐associated molecular patterns (PAMPs), damage‐associated molecular patterns (DAMPs), and local antigen and cytokine levels. Macrophages stimulated with innate immune receptors by pathogenic ligands produce proinflammatory cytokines and chemokines and lactate via upregulation of glycolysis (Rodriguez‐Prados *et al*, 2010). In models of rheumatoid arthritis, glycolysis‐mediated lactate produced by host tissues accumulated in inflamed synovial fluid and incited greater IL‐17 production upon uptake into CD4^+^ T cells, exacerbating joint destruction (Pucino *et al*, 2019). This suggests that host‐produced lactate can perpetuate and worsen inflammatory damage by reprogramming the intracellular metabolism of immune cells. We confirmed that lactate, proinflammatory cytokine, chemokine, and growth factor levels, including IL‐17, increased and accumulated in the synovial fluid of mice with MRSA septic arthritis. While the symptoms of septic arthritis were relieved by combined antibiotic treatment alone, the severity of intraarticular inflammation, which was observed to correspond with intraarticular lactate levels, failed to normalize. The progressive articular cartilage degradation and bone disruption secondary to joint inflammation in these septic arthritis models persisted following antibiotic treatment. This progressive joint destruction was still observed in the presence of HK‐MRSA and VT‐MRSA, which are characterized by reduced bacteria viability, suggesting that inflammation persists despite hindered bacterial proliferation, necessitating targeted anti‐inflammatory treatment for joint protection. DMF demonstrated greater anti‐inflammatory effects compared to other glycolysis inhibitors and reduced the production of proinflammatory markers by inactivating signaling through the NF‐κB and ERK‐mediated pathways in HK‐MRSA‐infected macrophages. DMF also normalized the production of proinflammatory markers by MRSA‐infected synovial cells and HK‐MRSA‐infected macrophages, which suggests that DMF can alleviate the secretion of proinflammatory factors produced by immune cells in response to live or dead bacteria. Combined antibiotic treatment with adjunctive systemic and intraarticular DMF treatment significantly reduced inflammation scores and the number of infiltrating immune cells, improving lameness in the setting of MRSA‐infected, septic joints. Articular cartilage and bone disruption was hindered by normalizing osteophyte formation and osteoclast differentiation with DMF treatment. Conversely, treatment with glucose uptake inhibitors was either ineffectual or exacerbated the symptoms of septic arthritis, suggesting that they were ineffective in suppressing joint disruption. These findings suggest that articular cartilage and bone disruption following infection persists via glycolysis‐mediated proinflammatory factors and that DMF may have an adjunctive role in the treatment of septic arthritis by protecting both cartilage and bone in septic joints. We suggest that synovial fluid lactate levels can serve as a potential joint fluid biomarker of septic arthritis. DMF has the potential to suppress the vicious cycle of excessive inflammation by inhibiting secondary inflammation mediated by lactate derived from primary immune and tissue cells.

A recent study showed that synovial fluid lactate and IL‐10 levels are increased in human periprosthetic joint infection patients (Heim *et al*, 2020). Reduction of lactate in biosynthesis‐defective *S. aureus* reduced levels of IL‐10, an anti‐inflammatory cytokine, altering neutrophil and immune cell infiltration following joint infection. These results suggest that increases in lactate, as secreted by bacterial glycolysis, facilitates persistent infection by activating anti‐inflammatory cytokines such as IL‐10 in the presence of a normally functioning innate immune system (Prince, 2020). This corroborates the theory that bacterial metabolites can exacerbate infectious disease progression by modulating immune cell activity and lends credence for the use of therapeutics targeting bacterial glycolysis. Previous studies have demonstrated that GAPDH is essential for glycolysis and plays an important role in the growth and virulence of staphylococcal species (Purves *et al*, 2010). Herein, we identified that DMF directly inhibits GADPH activity, thereby suppressing lactate secretion, MRSA growth, and MRSA biofilm formation. In synovial fluid cells extracted from MRSA‐infected models, DMF suppressed MRSA regrowth and lactate secretion and the production of inflammatory markers. Septic arthritis is characterized by high rates of recurrence due to the internalization of *S. aureus* by host cells (Hunter *et al*, 2015; Brandt *et al*, 2018; Moldovan & Fraunholz, 2019; Alder *et al*, 2020). Our results demonstrate that macrophage exposure to MRSA results in the intracellular proliferation of MRSA and reemergence into the extracellular environment over time. Although vancomycin was effective against extracellular MRSA, MRSA continued to proliferate in intracellular reservoirs. DMF inhibited the proliferation of intracellular MRSA, thereby reducing the extent of bacterial extravasation from the intracellular compartment for recalcitrant infection. The effectiveness of DMF may be mediated by the inhibition of glycolysis in host cells, which subsequently hinders intracellular MRSA survival and replication, or by dual inhibition of glycolysis in both host cells and MRSA. We identified that the sequence homology of the DMF‐targeting sequence in GAPDH was almost identical between Gram‐positive and ‐negative bacterial species including MRSA and *S. aureus*. Therefore, DMF inhibits diverse types of bacterial species growth and survival in both the intra‐ and extracellular environments and may be safely combined with conventional antibiotic therapy as an adjuvant cartilage and bone protection agent to improve the treatment of septic arthritis. DMF has the potential to inhibit the production of lactate derived from bacteria in adjuvant therapies for septic arthritis, thereby inhibiting lactate‐mediated metabolic reprogramming of host cells to improve the therapeutic efficacy of existing regimens to dually reduce bacterial bioburden and joint damage in septic arthritis.

In this study, we verified that adjuvant treatment of septic arthritis with both antibiotics and DMF to target glycolysis is efficacious in preventing the destructive sequelae of joint infection. However, our research has some limitations. First, in patients with septic arthritis, the disease is usually mediated by a bloodstream or periprosthetic joint infection (Carpenter *et al*, 2011). In this study, since the effect of DMF was verified in a model in which a large number of MRSA was injected into the knee joint, this model may represent patients with periprosthetic joint, but not bloodstream infection. Therefore, future studies will evaluate the adjuvant therapeutic effect of DMF in mitigating cartilage destruction in the setting of bloodstream or periprosthetic joint MRSA infection. In addition, septic arthritis caused by *S. aureus* species, including MRSA, is mediated by a number of virulence factors such as alpha‐toxin and staphylococcal coagulases and iron‐scavenging surface proteins secreted by the bacteria, as well as defective host responses involving innate immune and tissue cells (Pietrocola *et al*, 2017; Kwiecinski & Horswill, 2020). In this study, we demonstrate the efficacy and potential of DMF to regulate glycolysis‐mediated host responses to MRSA infection, which we show to be crucial to the propagation of intraarticular inflammation even following conventional antibiotic treatment. Future studies will evaluate the function of MRSA‐secreted virulence factors on disease pathogenesis and how, if any, these pathways are affected by DMF administration and glycolysis downregulation in the inflammatory joint micro‐environment. Taken together, we highlight the function of glycolysis and the potential of glycolysis‐targeted therapy to alleviate and/or obviate the risk of joint destruction in MRSA‐induced septic arthritis.

## Materials and Methods

### Cell culture and reagents

The murine macrophage cell line RAW264.7 was purchased from ATCC (San Diego, CA, USA; catalog no. ATCC® TIB‐71™) and cultured in high glucose Dulbecco's Modified Eagle's Medium (DMEM; Thermo Fisher Scientific, Inc., Waltham, MA, USA; catalog no. 11965118) containing 1% of penicillin/streptomycin solution (Thermo Fisher Scientific, Inc.; catalog no. 15070063) and 10% of fetal bovine serum (FBS; Thermo Fisher Scientific, Inc.; catalog no. 16140071). The primary mouse macrophage cells derived from bone marrow cells were performed on male C57BL/6J mice (6‐ to 8‐weeks‐old; Jackson Laboratory, Bar Harbor, Maine, USA) at a pathogen‐free animal facility. After euthanasia of the mice by CO_2_ in compliance with Institutional Animal Care and Use Committee guidelines, primary mouse monocyte cells were collected from femora and tibiae aseptically and flushed out with cold DMEM by means of a sterile 1 ml syringe. Red blood cells (RBC) contained in primary mouse monocyte cells were removed by RBC Lysis Buffer (Invitrogen, Carlsbad, CA, USA; catalog no. 00‐4333‐57). The remaining impurities were filtered through a 40 μm Nylon Cell Strainer (Corning Incorporated Life Science, NY, USA; catalog no. 431750). The suspended cells were incubated overnight in a complete DMEM solution containing 10% of FBS and 1% of a penicillin/streptomycin solution (Thermo Fisher Scientific, Inc.). After a day, the suspended cells were cultured with DMEM containing 10% of FBS, 1% of a penicillin/streptomycin solution, and 30 ng/ml of a macrophage colony‐stimulating factor (M‐CSF; R&D Systems Inc., Minneapolis, MN, USA; catalog no. 416‐ML or PeproTech, Rocky Hill, NJ, USA; catalog no. 315‐02) for 6–8 days. All cells were incubated in a humidified atmosphere containing 5% of CO_2_ at 37°C (Thermo Fisher Scientific, Inc.).

Vancomycin hydrochloride was purchased from Sigma‐Aldrich Co. (St. Louis, MO, USA; catalog no. 1709007). Rifampin was purchased from G‐Biosciences (St. Louis, MO, USA; catalog no. RC‐192). DMF was purchased from Acros Organics (Pittsburgh, PA, USA; catalog no. 222181000). Monosodium urate was purchased from InvivoGen (San Diego, CA, USA; catalog no. tlrl‐msu). 2‐Deoxy‐D‐glucose (2‐DG) and 3‐(3‐pyridinyl)‐1‐(4‐pyridinyl)‐2‐propen‐1‐one 1 (3PO) were purchased from Cayman Chemical Co. (Ann Arbor, MI, USA; catalog no. 14325 and 19276, respectively). DMSO was purchased from Spectrum Chemical MFG Corp. (Gardena, CA, USA; catalog no. D1258).

### MRSA culture and sample preparation

MRSA USA300‐FPR3757 expressive of GFP was provided by Alice Prince at Columbia University (Diep *et al*, 2006). A single MRSA colony was cultured on Mueller‐Hinton agar plates (Sigma‐Aldrich Co.; catalog no.70191) containing oxacillin (6 μg/ml; Sigma‐Aldrich Co.; catalog no. 1481000), then planktonically cultured in lysogeny broth (LB; Invitrogen; catalog no. 10855021) containing oxacillin (6 μg/ml; Sigma‐Aldrich Co.) in a 35°C incubator for 24 h, after which absorbance (600 nm) was measured with a NanoDrop™ 2000c Spectrophotometer (Thermo Fisher Scientific, Inc.). MRSA (2 × 10^9^ CFU/ml) was treated with vancomycin (20 mg/ml) for 24 h to make vancomycin‐treated MRSA (VT‐MRSA). MRSA (2 × 10^9^ CFU/ml) incubated at 65°C for 2 h to make heat‐killed MRSA (HK‐MRSA). These samples were washed by Dulbecco's phosphate‐buffered saline solution (DPBS; Thermo Fisher Scientific, Inc.; catalog no. 14190144) at least thrice before experimentation.

### RNA‐Sequencing analysis

Bone marrow from 6 to 8 weeks old female C57BL/6J mice femurs were flushed with MEM alpha (MEMα; catalog no. 12561056) medium containing 10% of fetal bovine serum (Gemini Bio, Woodland, CA, USA; catalog no. 100‐106) and 1% of penicillin/streptomycin solution (Thermo Fisher Scientific, Inc.). The bone marrow cells sat overnight in complete MEMα medium containing 10 ng/ml of M‐CSF (Shenandoah Biotechnology, Warwick, PA, USA; catalog no. 200‐08AF). The next morning, the floating cells were collected and seeded in 6‐well plates at 0.5 × 10^6^/well and grown in complete MEMα medium containing 10 ng/ml of M‐CSF for 2 days. The media was then changed with complete MEMα medium containing 20 ng/ml of M‐CSF for bone marrow‐derived macrophages (BMDM). A second set of cells was seeded in 12‐well plates at 0.15 × 10^6^/well and grown in complete MEMα medium containing 10 ng/ml of M‐CSF for 2 days. After 1 day, the media was changed to complete MEMα medium containing 20 ng/ml of M‐CSF and 80 ng/ml of RANKL (Shenandoah Biotechnology; catalog no. 200‐04AF) and allowed to sit for 3 more days, after which the medium was changed once more to obtain bone marrow‐derived osteoclasts (BMOC). Mouse osteoblasts (OB) were obtained by outgrowing mouse femoral bone chips in complete MEMα medium containing ascorbic acid (50 μg/ml, Sigma‐Aldrich Co.; catalog no. 1043003) and culturing these cells for 5 days. The medium was changed to antibiotic‐free medium before infection. The next day, the cells were either left uninfected or infected with MRSA (USA300) at a MOI of 10. After 24 h, the media were changed to MEMα containing 50 μg/ml of gentamicin (Sigma‐Aldrich Co.; catalog no. 1289003). At the end of each time point, the cells were washed twice with PBS, and RNA was harvested using a Qiagen RNeasy mini kit (Qiagen) according to the manufacturer's instructions. The quality and quantity of RNA samples were determined using Agilent bioanalyzer at Columbia Medical Center. 100 ng of total RNA were submitted and sequenced by the Columbia Genome Center. Briefly, the RNA library was prepared using a TruSeq® Stranded mRNA Library Prep kit (Illumina, San Diego, CA, USA) and Index Adapters were added using a TruSeq® RNA Single Indexes Set A kit (Illumina). The libraries were then mixed with NextSeq 500 v2.5 reagent kit and sequenced in an Illumina's NextSeq 500 sequencer. RNA‐Sequencing data can be found in the Sequence Read Archive (SRA) under the National Center for Biotechnology Information (accession number: PRJNA647064). Differentially expressed genes in MRSA infection were compared to the noninfectious conditions and defined as genes with a fold‐change ≥ 2 and a Benjamini–Hochberg adjusted *P*‐value < 0.05. Genes commonly expressed in all cells were selected through Venn diagram analysis and canonical pathways, upstream regulator factors, and biologic function were analyzed by Ingenuity Pathway Analysis (IPA; Qiagen Bioinformatics).

### Single‐cell RNA‐Sequencing analysis

We used the single‐cell RNA‐Sequencing data set using human synovial tissue isolated from rheumatoid arthritis patients (Zhang *et al*, 2019), which was uploaded and accessed via the ImPort (https://www.immport.org/shared/study/SDY998) or the Single Cell Portal hosted by Broad Institute of MIT and Harvard (https://portals.broadinstitute.org/single_cell/study/amp‐phase‐1). We analyzed expression levels of proteins from synovial fluid and tissue in a septic arthritis model using a single‐cell RNA‐Sequencing data set of rheumatoid arthritis patients using the Web‐based Single Cell Portal hosted by Broad Institute of MIT and Harvard.

### *In vivo* animal experiments

All animal experiments were approved by the Institutional Animal Care and Use Committee (IACUC; Number: 2020‐20129) of Yale University. Male C57BL/6J mice (10‐ to 14‐weeks old) were purchased from the Jackson Laboratory. Mice were anesthetized with a combination of ketamine (10 mg/ml; Ketaset®, Zoetis Inc., MI, USA) with xylazine (1 mg/ml; AnaSed® Injection, Akorn Inc., IL, USA). Fur surrounding the knee joint was removed using Veet® In Shower Cream (Reckitt Benckiser, Slough, UK) and softly washed with warm water. Skin overlying the knee was sterilized with povidone‐iodine pads and isopropyl alcohol pads (Professional Disposables International, Inc., NY, USA).

MRSA (0.25 × 10^6^, 1 × 10^6^, and 4 × 10^6^ CFU/10 μl) were intraarticularly injected under the patella using a U‐100 Micro‐Fine Insulin Syringe (28‐gauge needle; BD Biosciences) and sacrificed after 1–7 days. Control animals were injected with the same volume of DPBS (Thermo Fisher Scientific, Inc.).

MRSA (4 × 10^6^ CFU/10 μl) were intraarticularly injected under the patella using a U‐100 Micro‐Fine Insulin Syringe and sacrificed 24 h after infection. Control animals were injected with the same volume of DPBS (Thermo Fisher Scientific, Inc.).

For the single systemic antibiotic treatment condition, vancomycin (30 mg/kg) was subcutaneously injected using a U‐100 Micro‐Fine Insulin Syringe daily for 6 days after MRSA (4 × 10^6^ CFU/10 μl) infection of the knee joint and sacrificed at 7 and 14 days. Control animals were injected with the same volume of DPBS (Thermo Fisher Scientific, Inc.).

MRSA (2 × 10^7^ CFU/10 μl), HK‐MRSA, and VT‐MRSA were intraarticularly injected under the patella using U‐100 Micro‐Fine Insulin Syringe and sacrificed 7 days after infection. Control was intraarticularly injected with the same volume of DPBS (Thermo Fisher Scientific, Inc.).

For the combined systemic antibiotics treatment group, vancomycin (30 mg/kg) and/or rifampin (20 mg/kg) was subcutaneously injected using a U‐100 Micro‐Fine Insulin Syringe daily for 3 days after MRSA (4 × 10^6^ CFU/10 μl) infection of the knee joint and sacrificed 4 days after infection. The next day, WZB1177 (40 μg/joint) was intraarticularly injected using a U‐100 Micro‐Fine Insulin Syringe and sacrificed at the 14‐day timepoint. Controls were intraarticularly injected with the same volume of DPBS (Thermo Fisher Scientific, Inc.).

Vancomycin (30 mg/kg) was subcutaneously injected using a U‐100 Micro‐Fine Insulin Syringe daily for 3 days after MRSA (4 × 10^6^ CFU/10 μl) infection of the knee joint. The day after initial MRSA infection, rifampin (120 μg/joint) and/or WZB1177 (40 μg/joint) was intraarticularly injected using a U‐100 Micro‐Fine Insulin Syringe, after which mice were sacrificed at 14‐days. Controls were intraarticularly injected with the same volume of DPBS (Thermo Fisher Scientific, Inc.).

For the systemic or intraarticular DMF treatment groups, vancomycin (30 mg/kg) and rifampin (20 mg/kg) were subcutaneously injected using a U‐100 Micro‐Fine Insulin Syringe for 3 days following infection of the knee joint with MRSA (8 × 10^6^ CFU/10 μl). The next day, DMF was subcutaneously (14 mg/kg for the systemic treatment group) or intraarticularly (70 μg/joint for the intraarticular treatment group) administered using a U‐100 Micro‐Fine Insulin Syringe at 3‐day intervals for a total of 3 times, after which mice were sacrificed at the 14‐day time‐point. Controls were intraarticularly injected with the same volume of DPBS (Thermo Fisher Scientific, Inc.).

For the systemic or intraarticular DMF treatment groups, MSU (300 μg/joint) was intraarticularly injected using a U‐100 Micro‐Fine Insulin Syringe for 1 day. The next day, DMF was subcutaneously (14 mg/kg for the systemic treatment group) or intraarticularly (70 μg/joint for the intraarticular treatment group) administered using a U‐100 Micro‐Fine Insulin Syringe for 1 day; mice were sacrificed the following day. Controls were intraarticularly injected with the same volume of DPBS (Thermo Fisher Scientific, Inc.).

After intraarticular injection, the skin overlying the knee joint was sterilized as before. All mice were then placed on a warming pad and monitored until ambulatory. All mice were randomly grouped and housed at 22 ± 3°C on a 12‐h light and dark cycle in ventilated cages and were given chow and water *ad libitum*. Lameness was scored according to the reported experimental guideline (Kwon *et al*, 2021).

### *Ex vivo*: human cartilage and synovial tissue experiments

Specimens of human cartilage and synovial tissues were collected during knee joint surgery as was approved by the Institutional Review Board (IRB; Number: 2000021232) of Yale University. The experiments conformed to the principles set out in the WMA Declaration of Helsinki and the Department of Health and Human Services Belmont Report. All patients signed an informed consent form for participation for the use of their biological tissues. Tissues were transferred to a 12‐well plate (BD Biosciences) containing DMEM (Thermo Fisher Scientific, Inc.) with 10% FBS (Thermo Fisher Scientific, Inc.) and then infected with MRSA (4 × 10^6^ CFU) for 24 h. Tissue samples were fixed using PROTOCOL™ 10% Buffered Formalin (Thermo Fisher Scientific, Inc.; catalog no. 23‐245684) and embedded in paraffin for immunohistochemistry (IHC) and multiplex‐IHC (m‐IHC) analysis.

### *Ex vivo*: synovial fluid experiments

10 μl of DPBS (Thermo Fisher Scientific, Inc.) was intraarticularly injected under the patella using a U‐100 Micro‐Fine Insulin Syringe (28‐gauge needle; BD Biosciences), aspirated, and transferred to sterile microcentrifuge tubes. Synovial fluid cells were counted using a TC20™ Automated Cell Counter (Bio‐Rad Laboratories, Redmond, WA, USA). Cells (5 × 10^5^) were seeded on DMEM containing 10% of FBS with and without DMF (300 μg/ml) and incubated for 6 and 24 h. MRSA expressive of GFP was detected using the ZOE™ Fluorescent Cell Imager (Bio‐Rad Laboratories), and GFP intensity was measured using a BioTek Cytation™ 5 Cell Imaging Multi‐Mode Reader and analyzed using BioTek Gen5 software (BioTek Instruments Inc., Winooski, VT, USA). The production of lactate and proinflammatory cytokines, chemokines, and growth factors in supernatant medium was measured as described below.

### *Ex vivo*: whole‐blood experiment

Fresh blood was collected by cardiac puncture and divided into 150 μl portions into separate BD Microtainer Tube Blood Collection Lithium Heparin (BD Biosciences) vials. DMF was administered for 1 h, after which the blood was infected with HK‐MRSA (4 × 10^6^ CFU) for 6 h. Serum was isolated by centrifugation (1,107 *g*) for 30 min. The supernatant was subsequently used for cytokines, chemokines, and growth factors multiplex analysis.

### *In vitro* experiments

RAW264.7 (5 × 10^4^) cells were seeded on 96‐well plates and then treated with 2‐DG, 3PO, and DMF at concentrations of 4–500 μM for 1 h. These cells were infected with HK‐MRSA (4 × 10^6^ CFU) for 12 h, which were subsequently used for enzyme‐linked immunosorbent, lactate dehydrogenase (LDH), and cell viability assays.

RAW264.7 (1 × 10^6^) cells were seeded on 6‐well plates for Western blot assay and (5 × 10^4^) cells were seeded on 96‐well plates for immunocytochemistry assay. Cells were treated with DMF (20 μM) for 1 h and then infected with HK‐MRSA (4 × 10^6^ CFU) for 30 min.

BMDM (5 × 10^5^) cells were seeded on 24‐well plates and infected with HK‐MRSA (4 × 10^6^ CFU) for 24 h, which were subsequently used for lactate and enzyme‐linked immunosorbent assays.

BMDM (5 × 10^5^) cells were seeded on 24‐well plates and treated with DMF (50 μg/ml) for 1 h and then infected with HK‐MRSA (4 × 10^6^ CFU) for 24 h. BMDM (5 × 10^5^) cells were seeded on 24‐well plates and infected with HK‐MRSA (4 × 10^6^ CFU) for 1 h and then treated with DMF (50 μg/ml) for 24 h. These samples were used for enzyme‐linked immunosorbent assays.

### Complete blood count analysis

Blood was collected by cardiac puncture using a 1 ml BD Slip‐Tip Disposable Tuberculin Syringe (28‐gauge; BD Biosciences) and then immediately mixed with a 0.5 M EDTA pH 8.0 solution (1:10 ratio; AmericanBio, Natick, MA, USA; catalog no. AB00502) in sterile microcentrifuge tubes. CBC analysis, including the number and/or percentage of white blood cells, lymphocytes, monocytes, and neutrophils, was performed using the Abaxis VetScan HM5 Hematology System (Abaxis North America, Union City, CA, USA) according to the manufacturer's instructions.

### Synovial fluid collection and analysis

10 μl of DPBS (Thermo Fisher Scientific, Inc.) was intraarticularly injected under the patella using a U‐100 Micro‐Fine Insulin Syringe (28‐gauge needle; BD Biosciences), aspirated, and transferred to sterile microcentrifuge tubes. Synovial fluid cell number (1:10 dilution) was measured using a TC20™ Automated Cell Counter (Bio‐Rad Laboratories); MRSA expressive of GFP in synovial fluid was detected using the ZOE™ Fluorescent Cell Imager (Bio‐Rad Laboratories). Synovial fluid cells (1 × 10^4^) were seeded on Mueller–Hinton agar plates (Sigma‐Aldrich Co.) containing oxacillin (6 μg/ml) and incubated for 24–48 h. The images were captured with a ChemiDoc™ Touch Imaging System (Bio‐Rad Laboratories), and MRSA colony forming units (CFU) were quantified using ImageJ software (Schneider *et al*, 2012).

### Enzyme‐linked immunosorbent assay analysis

The levels of IL‐6 and TNF‐α in supernatant medium were measured using an IL‐6 and TNF‐α ELISA Ready‐SET‐Go!™ Kit (Invitrogen; catalog no. 88‐7064‐88 and 88‐7324‐88, respectively) according to the manufacturer's instructions. The level of IL‐6 and TNF‐α was measured with a BioTek Cytation™ 5 Cell Imaging Multi‐Mode Reader and analyzed using BioTek Gen5 software (BioTek Instruments Inc.).

### Lactate dehydrogenase analysis

The levels of LDH in supernatant medium were measured using CyQUANT™ LDH Cytotoxicity Assay (Invitrogen; catalog no. C20301) according to the manufacturer's instructions; the LDH value measured in nontreatment using the lysis buffer was set to 100% of the cytotoxicity. The level of LDH was measured with a BioTek Cytation™ 5 Cell Imaging Multi‐Mode Reader and analyzed using BioTek Gen5 software (BioTek Instruments Inc.).

### Cell viability analysis

Cell viability was measured using RealTime‐Glu™ MT Cell Viability Assay (Promega Corporation, WI, USA; catalog no. G9711) according to the manufacturer's instructions. The luminescence signal was measured using a BioTek Cytation™ 5 Cell Imaging Multi‐Mode Reader and analyzed using BioTek Gen5 software (BioTek Instruments Inc.) at intervals of 30 min for 12 h total under a humidified atmosphere containing 5% of CO_2_ at 37°C.

### Lactate analysis

The level of lactate in supernatant medium (1:50 dilution by assay buffer) was measured using the Lactate‐Glo™ Assay kit (Promega Corporation; catalog no. J5021) according to the manufacturer's instructions. Lactate levels in the synovial fluid and serum (1:50 dilution by assay buffer) were measured using the Lactate‐Glo™ Assay kit according to the manufacturer's instructions. Lactate levels in supernatant medium (1:50 dilution by assay buffer), derived from *ex‐vivo* experiments, were measured using the Lactate‐Glo™ Assay kit according to the manufacturer's instructions.

MRSA (4 × 10^6^ CFU) was cultured in LB broth with or without DMF (600 μg/ml) for 1 h. The level of lactate in LB broth without MRSA was measured using the Lactate‐Glo™ Assay according to the manufacturer's instructions. Lactate levels were measured with a BioTek Cytation™ 5 Cell Imaging Multi‐Mode Reader and analyzed using BioTek Gen5 software (BioTek Instruments Inc.). Luminescence intensity was detected by the ChemiDoc™ Touch Imaging System (Bio‐Rad Laboratories).

### Multiplex cytokines, chemokines, and growth factors analysis

Synovial fluid samples derived from *in vivo* models were mixed with assay buffer (1:25 dilution). Serum samples derived from *in vivo* models were mixed with assay buffer (1:5 dilution). Supernatant medium derived from *ex vivo* experiments were used as an original medium. Synovial fluid, serum, and supernatant medium samples were submitted to the Immune Monitoring Core Facility at Yale University and the number of cytokines, chemokines, and growth factors were measured by MILLIPLEX MAP Mouse Cytokine/Chemokine Magnetic Bead Panel‐Immunology Multiplex Assay from Millipore (Billerica, MA, USA; catalog no. MCYTMAG‐70K‐PX32) according to the manufacturer's instructions. Principal component analysis (PCA) was performed with QStudioMetrics (https://github.com/gmrandazzo/QStudioMetrics).

### RNA extraction and real‐time polymerase chain reaction analysis

Total RNA in mouse knee joint tissues was isolated using a GE Healthcare Illustra™ RNAspin Mini Isolation Kit (GE Healthcare, Madison, WI, USA; catalog no. 25050072) according to the manufacturer's instructions. RNA concentration was measured with a NanoDrop™ 2000c Spectrophotometer (Thermo Fisher Scientific, Inc.), and cDNA was synthesized with ReverTra Ace® qPCR RT Master Mix (Toyobo, Osaka, Japan; catalog no. FSQ‐201) according to the manufacturer's instructions. mRNA expression was evaluated by a Thunderbird® SYBR® qPCR Mix (Toyobo; catalog no. QPS‐201) mixed with specific primer sequences; *Slc16a3*: 5′‐TGCCAGTCTCTGGACCTCTT‐3′ and 5′‐GCAGAGCCTGAGAAAAATGG‐3′; *Slc2a1*: 5′‐TTCTCTGTCGCCCTCTTTGT‐3′ and 5′‐GAGAAGCCCATAAGCACAGC‐3′; *Gapdh*: 5′‐GTGGAGTCATACTGGAACATGTAG‐3′ and 5′‐AATGGTGAAGGTCGGTGTG‐3′ according to the manufacturer's instructions. Gene expression was measured by using a StepOnePlus™ Real‐Time PCR System (Applied Biosystems, Foster City, CA, USA), from which relative mRNA expression was normalized with *Gapdh* using the $2^{-\Delta \Delta C_{t}}$ method. mRNA expression was evaluated using a SsoFast™ EvaGreen® Supermix with Low ROX (Bio‐Rad Laboratories; catalog no. #1725211) kit mixed with specific primer sequences; *Il‐6*, *Il‐1b*, *Ccl3*, and *Cxcl10* (Kwon *et al*, 2021) according to the manufacturer's instructions. Gene expression was measured by using a ViiA™ 7 Real‐Time PCR System (Applied Biosystems), from which relative mRNA expression was normalized with *Gapdh* using the $2^{-\Delta \Delta C_{t}}$ method.

### Immunohistochemistry analysis

IHC was performed according to the reported experimental materials and protocols (Kwon *et al*, 2021) using slides of MRSA‐infected knee joint tissues. Slides were incubated with primary antibodies (1:200 dilution) such as GLUT1 (Abcam; cat. Number: ab115730), MCT4 (Abcam; catalog no. ab180699), IL‐6 (Thermo Fisher; catalog no. AHC0762), IL‐1β (Abcam; catalog no. ab9722), and MMP3 (Abcam; catalog no. ab52915). Images were detected with a BioTek Cytation 5 Cell Imaging Multi‐Mode Reader and analyzed by BioTek Gen5 software (Bio‐Tek Instruments Inc.). The percentage of staining positive cells was counted using QuPath software (Bankhead *et al*, 2017).

### Histological evaluation

Knee joint tissue sections were then embedded in paraffin and stained with hematoxylin and eosin (H&E) and safranin O (SAF O) according to the manufacturer's instructions. Images of the stained knee joint tissues were captured with a BioTek Cytation 5 Cell Imaging Multi‐Mode Reader and analyzed by BioTek Gen5 software (Bio‐Tek Instruments Inc.). H&E‐stained images were used for inflammation scoring and to characterize synovial hyperplasia and synovial cellularity according to previous guidelines (Kwon *et al*, 2021). SAF O‐stained images were used to generate OARSI scores and to characterize osteophyte formation, proteoglycan depletion, bone erosion, and bone formation according to previous guidelines (Kwon *et al*, 2021).

### Multiplex‐immunohistochemistry analysis

Slides of MRSA‐infected human cartilage tissues and knee joint tissues were used for multiplex‐IHC analysis using primary antibodies (1:200 dilution) such as GLUT1 (Abcam; catalog no. ab115730) and MCT4 (Abcam; catalog no. ab180699), IL‐1β (Abcam; catalog no. ab9722), NLRP3 (AdipoGen, San Diego, CA, USA; catalog no. AG‐20B‐0014‐C100), MMP3 (Abcam; catalog no. ab52915), and NF‐κB p65 (Cell Signaling Technology Inc.; catalog no. #8242) according to the reported experimental materials and protocols (Kwon *et al*, 2021). Fluorescence was detected with a BioTek Cytation 5 Cell Imaging Multi‐Mode Reader and analyzed by BioTek Gen5 software (Bio‐Tek Instruments Inc.).

### Protein extraction and Western blot analysis

Extraction of whole protein from synovial tissues was performed using Tissue Protein Extraction Reagent (T‐PER; containing a Protease and Phosphatase Inhibitor Cocktail; Thermo Fisher Scientific, Inc.; catalog no. 78510 and 78440, respectively) according to the manufacturer's instructions. Extraction of whole protein from macrophage cells was performed using Mammalian Protein Extraction Reagent (M‐PER; containing a Protease and Phosphatase Inhibitor Cocktail; Thermo Fisher Scientific, Inc.; catalog no. 78501 and 78440, respectively) according to the manufacturer's instructions. Extraction of nuclear and cytoplasmic protein from macrophage cells was performed using NE‐PER Nuclear and Cytoplasmic Extraction Reagents (NE‐PER; containing a Protease and Phosphatase Inhibitor Cocktail; Thermo Fisher Scientific, Inc.; catalog no. 78833 and 78440, respectively) according to the manufacturer's instructions. Protein concentration was measured and normalized with a bicinchoninic acid (BCA) protein assay kit (Thermo Fisher Scientific, Inc.; catalog no. 23225) according to the manufacturer's instructions. Western blot was performed according to the reported experimental materials and protocols (Kwon *et al*, 2021). Membranes were immunoblotted with primary antibodies (1:1,000 dilution), such as GLUT1 (Abcam; catalog no. ab115730), MCT4 (Abcam; catalog no. ab180699), cleaved‐IL‐1β (Abcam; catalog no. ab52718), NLRP3 (AdipoGen; catalog no. AG‐20B‐0014‐C100), MMP3 (Abcam; catalog no. ab52915), p‐ERK (Cell signaling Technology Inc.; catalog no. #4370), ERK (Cell signaling Technology Inc.; catalog no. #4695), p‐p38 (Cell signaling Technology Inc.; catalog no. #4511), p38 (Cell signaling Technology Inc.; catalog no. #8690), p‐JNK (Cell signaling Technology Inc.; catalog no. #9251), JNK (Cell signaling Technology Inc.; catalog no. #9252), NF‐κB (Cell signaling Technology Inc.; catalog no. #8242), IκBα (Cell signaling Technology Inc.; catalog no. #4814), Lamin A/C (Cell signaling Technology Inc.; catalog no. #4777), and GAPDH (Cell signaling Technology Inc.; catalog no. #2118) and incubated on a shaker at 4°C overnight. The following day, membranes were treated with HRP‐conjugated antimouse or antirabbit immunoglobulin G antibody (1:1,000 dilution, Cell Signaling Technology Inc.; catalog no. #7076 and #7074, respectively) for 1 h. Protein expression levels were detected by the ChemiDoc™ Touch Imaging System (Bio‐Rad Laboratories) using SuperSignal West Pico PLUS Chemiluminescent Substrate or SuperSignal West Femto Maximum Sensitivity Substrate (Thermo Fisher Scientific, Inc.). Band intensities were measured with ImageJ software (Schneider *et al*, 2012). Protein expression levels were normalized to GAPDH expression levels, and expression levels of phosphorylated proteins were normalized to nonphosphorylated proteins expression levels.

### GAPDH sequence alignment and phylogenetic tree analysis

The sequence alignment and phylogenetic tree of GAPDH in *Homo sapiens* (UniProt: P04406), *Mus musculus* (UniProt: P16858), *Staphylococcus aureus* (UniProt: P0A038), *Staphylococcus aureus* (USA300; UniProt: A0A0H2XHX6), *Staphylococcus aureus* (MRSA252; UniProt: Q6GIL8), *Salmonella typhimurium* (14028s; UniProt: A0A0F6B0L7), *Escherichia coli* (UniProt: C3T6W2), *Mycobacterium tuberculosis* (UniProt: A0A045ITJ4), *Streptococcus pneumoniae* (UniProt: I6L8L9), *Neisseria gonorrhoeae* (UniProt: Q83UU4), and *Pseudomonas aeruginosa* (UniProt: A0A4U9LV61) were analyzed by UniProt Align (https://www.uniprot.org/align).

### GAPDH activity analysis

A single MRSA colony was planktonically cultured in LB (Invitrogen) medium containing oxacillin (6 μg/ml; Sigma‐Aldrich Co.) for 24 h. MRSA (2 × 10^7^ CFU) was lysed by application of Bacterial Protein Extraction Reagent (B‐PER; Thermo Fisher Scientific, Inc. catalog no. 78243) containing lysozyme (5 mg/ml; Thermo Fisher Scientific, Inc. catalog no. 90082), lysostaphin (0.5 mg/ml; Sigma‐Aldrich Co. catalog no. L7386), and Protease and Phosphatase Inhibitor Cocktail (Thermo Fisher Scientific, Inc.) for 37°C for 1 h. Proteins were isolated by centrifugation (20,784 *g* for 10 min; Thermo Fisher Scientific, Inc.) and treated with DMF (150, 300, and 600 μg/ml) for 1 h. GAPDH activity was subsequently measured using a GAPDH activity assay kit (Abcam, Cambridge, MA, USA, catalog no. ab204732) according to the manufacturer's instructions. Absorbance (450 nm) was detected using BioTek Cytation™ 5 Cell Imaging Multi‐Mode Reader and analyzed using BioTek Gen5 software (BioTek Instruments Inc.).

### Disk diffusion analysis

A single MRSA colony was planktonically cultured in LB (Invitrogen) medium containing oxacillin (6 μg/ml; Sigma‐Aldrich Co.) for 24 h. The medium containing MRSA was spread onto Mueller‐Hinton agar plates (Sigma‐Aldrich Co.) with oxacillin (6 μg/ml) using a sterile Cotton‐Tipped Applicator (McKesson Medical, San Francisco, CA, USA) and grown in a 35°C incubator for 1 h. Different concentrations of DMF (75, 150, and 300 μg) and vancomycin (1,000 μg) were prepared and 20 μl of each sample was loaded onto sterile blank paper discs (6 mm; Thermo Fisher Scientific, Inc.; catalog no. S70150A) and dried for 1 h. The dried discs were transferred to a plate and then incubated for 24 h. The images were captured with a ChemiDoc™ Touch Imaging System (Bio‐Rad Laboratories) which the diameter of each inhibition zone was measured using ImageJ software (Schneider *et al*, 2012).

### Growth analysis

Different concentrations of DMF (140, 420, and 700 μg/ml) and vancomycin (2.5 mg/ml) were added to LB (200 μl; Invitrogen) medium containing oxacillin (6 μg/ml; Sigma‐Aldrich Co.) in 96‐well plates (BD Biosciences); DMSO was used as the negative control. MRSA (4 × 10^6^ CFU) was seeded into the medium, and absorbance (600 nm) was measured using BioTek Cytation™ 5 Cell Imaging Multi‐Mode Reader (BioTek Instruments Inc.) in a 35°C incubator at intervals of 1 h for a total of 24 h. Data were analyzed using BioTek Gen5 software (BioTek Instruments Inc.) (Movie EV1).

For the antibiotic effect analysis, MRSA (4 × 10^6^ CFU) was seeded into LB (200 μl; Invitrogen) medium containing oxacillin (6 μg/ml; Sigma‐Aldrich Co.) in 96‐well plates (BD Biosciences) and incubated in a 35°C at intervals of 1 h for a total of 4 h. Different concentrations of DMF (140, 420, and 700 μg/ml) were added to the medium, and absorbance (600 nm) was measured using a BioTek Cytation™ 5 Cell Imaging Multi‐Mode Reader and analyzed using BioTek Gen5 software (BioTek Instruments Inc.) at intervals of 1 h for 20 h total; DMSO was used as the negative control. *Pseudomonas aeruginosa* strain PA01 was grown in tryptic soy broth (TSB; BD Biosciences, catalog no. 211825) at 37°C overnight as shown in our publication (Peng *et al*, 2022), and *Streptococcus pneumoniae* strain TIGER4 was purchased from ATCC and grown in a humidified incubator with 5% CO_2_ in Todd Hewitt broth (THB; BD Biosciences, catalog no. 249240). *Staphylococcus aureus* strain RN4220 and ΔgapA *Staphylococcus aureus* strain RN4220, which were kindly provided by Julie A. Morrissey at the University of Leicester (Purves *et al*, 2010), were grown in TBS. ΔgapA *Staphylococcus aureus* strain RN4220 was selected using tetracycline (Sigma‐Aldrich Co., catalog no. 87128) plates. To test the effectiveness of DMF, the overnight cultures of bacteria were subcultured at a 1:10 ratio and mixed with various concentrations of DMF in a 96‐well plate (Corning Incorporated Life Science). The bacterial growth was measured by measuring absorbance (600 nm) over a period of time using an automated reader (BioTek Cytation 3 imaging reader, BioTek Instruments Inc.) at 37°C with 10 s of shaking before measurement; DMSO was used as the negative control.

### Biofilm formation analysis

Different concentrations of DMF (140, 420, and 700 μg/ml) and vancomycin (2.5 mg/ml) were added to LB (200 μl; Invitrogen) medium containing oxacillin (6 μg/ml; Sigma‐Aldrich Co.) in 96‐well plates (BD Biosciences). MRSA (4 × 10^6^ CFU) was seeded into the medium and incubated for 24 h; DMSO was used as the negative control. MRSA (4 × 10^6^ CFU) seeded into LB (200 μl; Invitrogen) medium containing oxacillin (6 μg/ml; Sigma‐Aldrich Co.) in 96‐well plates (BD Biosciences) and incubated at 35°C for 4 h. Different concentrations of DMF (140, 420, and 700 μg/ml) were applied to medium for 20 h; DMSO was used as the negative control.

The media was removed and slides were fixed with methanol (J.T. Baker, Center Valley, PA, USA; catalog no. 909302) for 10 min and then washed with DPBS (Thermo Fisher Scientific, Inc.) thrice. 200 μl of crystal violet solution (0.1%; Ward's Natural Science, NY, USA; catalog no. 548‐62‐9) was then applied and allowed to incubate for 15 min, after which the slides were washed with DPBS (Thermo Fisher Scientific, Inc.) thrice. 200 μl of acetic acid (33%; Sigma‐Aldrich Co.; catalog no. 695092) was added, and absorbance (570 nm) was measured using a BioTek Cytation™ 5 Cell Imaging Multi‐Mode Reader and analyzed using BioTek Gen5 software (BioTek Instruments Inc.).

### Intracellular MRSA analysis

RAW264.7 (1 × 10^4^) cells were seeded on 96‐well plates (BD Biosciences) and stained with CellTracker™ Red CMTPX Dye according to the manufacturer's instructions. Cells were infected with MRSA (4 × 10^6^ CFU) for 2 h and washed 3 times to remove extracellular MRSA. DMEM containing DMF (700 μg/ml), vancomycin (100 μg/ml), and DMF (700 μg/ml) with vancomycin (100 μg/ml) were added, and GFP intensity was measured and fluorescence using CellTracker was detected at 1‐h intervals for 12 h total (Movie EV2) using a BioTek Cytation™ 5 Cell Imaging Multi‐Mode Reader and analyzed using BioTek Gen5 software (BioTek Instruments Inc.). Intracellular and extracellular MRSA were semi‐quantitatively measured at 12 h using QuPath software (Bankhead *et al*, 2017).

### Statistical analysis

All experimental data was analyzed using one‐way analysis of variance (ANOVA) or two‐tailed unpaired *t*‐test in GraphPad Prism Version 7 and 8 (GraphPad Software, Inc., La Jolla, CA, USA). *In vitro* experiments were repeated at least three times, and *In vivo* experiments were repeated in at least two independent experiments. All statistical parameters, *P*‐values, *n* numbers, and other relevant detailed information are reported in figure legends.

**The paper explained**

**Problem**

Recurrent bacterial infections of bone and joints are very difficult to treat due to the re‐emergence and hardy survival of intracellularly hidden bacteria. Although large amounts of lactate, a product of anaerobic glycolysis, have been observed in the synovial fluid of patients with septic arthritis, the functional significance of glycolysis in host inflammation and bacterial proliferation in the context of septic arthritis have not been evaluated.

**Results**

We confirmed that the production of inflammatory markers and glycolysis products increases in methicillin‐resistant *Staphylococcus aureus* (MRSA)‐infected bone and immune cells using RNA‐sequencing analysis. We observed the accumulation of inflammatory cytokines and lactate intraarticularly in a clinically relevant murine model of MRSA‐induced septic arthritis and verified these findings in MRSA‐infected human synovial and cartilage tissues. We verified that high concentrations of lactate and proinflammatory markers persisted within the joint space even after conventional antibiotic treatment. Adjuvant therapy with glucose uptake inhibitors in combination with antibiotics was ineffective, but dimethyl fumarate (DMF), which selectively targets the glycolysis pathway, was more effective in reducing the production and secretion of proinflammatory markers than other tested drug candidates. Moreover, DMF inhibited MRSA proliferation and biofilm formation via bacteriostasis and improved the efficacy of vancomycin, which is ineffective against intracellular MRSA, by successfully hindering the proliferation of intracellular MRSA. Both systemic and intraarticular adjunctive DMF administration after antibiotic treatment yielded more favorable prognoses inclusive of cartilage and bone protection, inflammation reduction, and reduced lameness in the setting of murine septic arthritis.

**Impact**

We provide evidence of the potential of repurposing DMF to ameliorate septic arthritis by exerting bacteriostatic effects upon MRSA growth and mitigating intraarticular inflammation that persists after conventional antibiotic treatment. Our results suggest that glycolysis may serve as a viable therapeutic target for the improved treatment of septic arthritis and synovial lactate may be a useful diagnostic marker of septic arthritis.

## Author contributions

**Francis Y Lee:** Conceptualization; resources; formal analysis; supervision; funding acquisition; validation; investigation; methodology; writing—original draft; project administration; writing—review and editing. **Hyuk‐Kwon Kwon:** Conceptualization; resources; data curation; software; formal analysis; supervision; validation; investigation; visualization; methodology; writing—original draft; project administration; writing—review and editing. **Kristin E Yu:** Data curation; formal analysis; writing—original draft; writing—review and editing. **Sean V Cahill:** Data curation; formal analysis; writing—original draft; writing—review and editing. **Kareme D Alder:** Data curation; formal analysis; writing—original draft; writing—review and editing. **Christopher M Dussik:** Data curation; formal analysis; writing—original draft; writing—review and editing. **Sang‐hun Kim:** Data curation; formal analysis; writing—original draft; writing—review and editing. **Lokesh Sharma:** Data curation; formal analysis; writing—original draft; writing—review and editing. **Jungho Back:** Conceptualization; data curation; formal analysis; writing—original draft; writing—review and editing. **Irvin Oh:** Conceptualization; writing—original draft; writing—review and editing.

## Disclosure and competing interests statement

The authors declare that they have no conflict of interest.

## For more information

- ImPort: https://www.immport.org/shared/study/SDY998
- Single Cell Portal: https://portals.broadinstitute.org/single_cell/study/amp‐phase‐1

## Supporting information



Appendix

Expanded View Figures PDF

Movie EV1

Movie EV2

Source Data for Expanded View

PDF+

Source Data for Figure 2

Source Data for Figure 3

## Data Availability

The RNA‐sequencing data have been deposited to the Sequence Read Archive (SRA) under the National Center for Biotechnology Information (accession number: PRJNA647064; Title: Transcriptome profiling of bone marrow‐derived macrophage, osteoblast, and osteoclast cells infected with MRSA).
