# Supplementary material for: Concurrent targeting of glycolysis in bacteria and host cell inflammation in septic arthritis
Source: EMBO Mol Med. 2022 Nov 10;14(12):e15284. doi: 10.15252/emmm.202115284 (PMC9728052; doi:10.15252/emmm.202115284)
Supplement: Supplementary file 1 — Appendix [file EMMM-14-e15284-s007.pdf]

Appendix Figure

Table of contents

Appendix Fig. S1 Analysis results of RNA-sequencing, which is related to Fig 1A-C.

Appendix Fig. S2 Septic arthritis caused by MRSA infection induces inflammation and glycolysis, which is related to Fig 1.

Appendix Fig. S3 Single-cell RNA-sequencing results from synovial tissue—including different types of B cells, fibroblasts, monocytes, and T cells—derived from rheumatoid arthritis patients.

Appendix Fig. S4 GLUT1 expression using immunohistochemistry from antibiotic treatment of septic arthritis, which is related to Fig 2F.

Appendix Fig. S5 Analysis of NF-κB translocation into the nucleus, which is related to Fig 4F.

Appendix Fig. S6 Analysis of alignment of an amino acid sequence of GAPDH, which is related to Fig 5D.

A

Ingenuity Canonical Pathways	-log(p-value)	Ratio	
Osteoarthritis Pathway	5.54	0.0392	VEGFA,DDIT4,GREM1,PTGS2,CEBPB,SDC4,NOS2,TNF
IL-6 Signaling	3.58	0.0382	VEGFA,IL1A,NFKBIA,CEBPB,TNF
Acute Phase Response Signaling	3.13	0.0305	IL1A,NFKBIA,Saa3,CEBPB,TNF
PPAR Signaling	3.04	0.0404	IL1A,NFKBIA,PTGS2,TNF
LXR/RXR Activation	2.87	0.0364	IL1A,PTGS2,NOS2,TNF
HMGB1 Signaling	2.58	0.0303	IL1A,VCAM1,LIF,TNF
Dendritic Cell Maturation	2.18	0.0234	IL1A,NFKBIA,CD83,TNF
Colorectal Cancer Metastasis Signaling	1.63	0.016	VEGFA,PTGS2,NOS2,TNF

B

Upstream Regulator	-log(p-value)	Target molecules in dataset
HIF1A	26.80	ADM,APLN,BNIP3,Ccl2,CXCL2,CXCL3,Cxcl3,DDIT4,EGLN3,HK2,IL1A,NDRG1,NOS2,PFKFB3,PFKL,PTGS2,SLC16A3,SLC2A1,TNF,TNFAIP2,VEGFA
CREM	17.83	ATF3,BHLHE40,BTG2,CEBPB,CSRNP1,GADD45B,JUNB,NFIL3,NR4A1,PPP1R15A,Sik1,TIPARP,TNF,VEGFA
CREB1	16.77	ATF3,BHLHE40,BTG2,CEBPB,CSRNP1,GADD45B,JUNB,NFIL3,NR4A1,PPP1R15A,Sik1,TIPARP,VEGFA
NFKBIA	12.62	Ccl2,CCL3L3,Ccl7,CXCL2,CXCL3,IL1A,LIF,PTGS2,TNF
EGR1	12.26	Ccl2,CCL3L3,Ccl7,CXCL2,CXCL3,F3,JUNB,NDRG1,TNF,VCAM1
RELA	10.77	Ccl2,CXCL2,CXCL3,IL1A,NFKBIA,NOS2,NR4A1,PTGS2,Saa3,TNF
KLF6	9.38	Ccl2,CCL3L3,IL1A,NOS2,PTGS2,TNF
PARP1	6.94	Ccl2,CXCL3,NOS2,PTGS2,TNF,VCAM1
NFATC2	6.59	CXCL3,EGR3,INHBA,IRF1,NFKBIZ,PTGS2,TNF
CEBPB	6.45	APLN,CEBPB,IER3,NOS2,PPP1R15A,PTGS2,Saa3,TNF
SPIB	6.17	ATF3,GEM,Gm6377,HSPA1A/HSPA1B,Hspa1b,KDM6B
STAT4	5.34	BCL3,CXCL2,CXCL3,KDM6B,TNF
STAT1	4.32	Ccl2,CCRL2,IRF1,NOS2,Slfn2,TNF
NR5A2	3.97	CEBPB,Hspa1b,JUNB,TNF
ATF4	3.91	ATF3,CEBPB,DDIT4,NDRG1,VEGFA
MYC	3.25	HK2,PFKFB3,PFKL,SLC16A3,SLC2A1
CTNNB1	2.82	BHLHE40,GADD45B,GREM1,PTGS2,Saa3,SPRY4,VCAM1

C

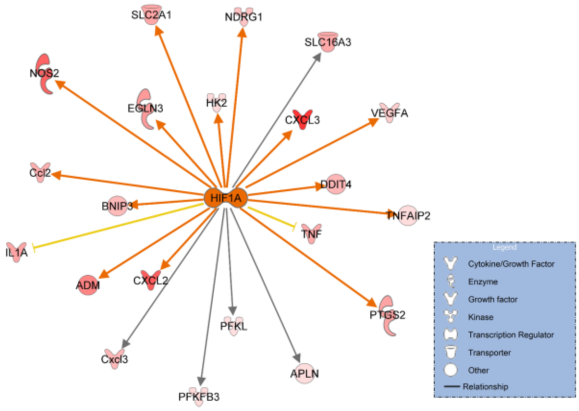

	Log$_2$ expression				Log$_2$ expression		
Gene	OB	BMOC	BMDM	Gene	OB	BMOC	BMDM
SLC16A3	3.24	2.29	1.51	HK2	1.38	3.21	1.36
SLC2A1	3.27	3.19	2.06	IL1A	3.35	8.84	8.48
ADM	4.60	6.56	4.23	NDRG1	2.25	2.88	2.97
APLN	1.24	4.59	2.32	NOS2	5.85	7.84	7.14
BNIP3	2.58	2.19	1.99	PFKFB3	1.34	3.21	3.23
Ccl2	2.98	1.98	1.32	PFKL	1.10	1.65	1.02
CXCL2	7.65	7.60	6.07	PTGS2	3.41	8.45	5.32
CXCL3	2.69	5.94	5.17	TNF	3.16	5.57	3.49
DDIT4	2.70	5.06	3.09	TNFAIP2	1.24	1.19	1.77
EGLN3	3.76	3.99	2.47	VEGFA	1.91	2.83	1.75

D

Categories	Diseases or Functions Annotation	-log(p-value)	Molecules	Molecules
Carbohydrate metabolism	Quantity of carbohydrate	7.52	APLN,Ccl2,CEBPB,DUSP1,HK2,JUNB,NOS2,P2RY2,PFKFB3,PPP1R15A,PTGS2,RGS16,Sik1,SLC2A1,TNF	15
	Glycolysis of cells	6.43	DDIT4,IER3,NOS2,PFKFB3,SLC16A3,SLC2A1	6
	Uptake of monosaccharide	5.46	Ccl2,CEBPB,NR4A3,PFKFB3,PPP1R15A,PTGS2,SLC2A1,TNF	8
	Metabolism of carbohydrate	5.28	APLN,CEBPB,CXCL3,HK2,NOS2,NR4A1,PFKL,PPP1R15A,SLC2A1,TNF	10
	Quantity of monosaccharide	5.00	Ccl2,CEBPB,HK2,NOS2,PFKFB3,PPP1R15A,RGS16,Sik1,SLC2A1,TNF	10
	Gluconeogenesis	4.60	ATF3,Ccl2,CEBPB,DUSP1,Sik1	5
	Transport of D-glucose	4.42	HK2,NR4A1,NR4A3,SLC2A1,TNF	5
	Uptake of 2-deoxyglucose	4.38	Ccl2,PFKFB3,PTGS2,SLC2A1,TNF	5

Appendix Fig. S1 Analysis results of RNA-sequencing, which is related to Fig 1A-C.

Primary osteoblast (OB), bone-marrow-derived osteoclast (BMOC), and bone-marrow-derived macrophage (BMDM) cells were infected with MRSA, the transcriptome expression profiles of which were analyzed by RNA-sequencing. The expression of 85 genes increased in the setting of MRSA infection.

A. related to Fig 1B. Detailed information on canonical pathways.

B. related to Fig 1B. Detailed information on upstream regulators.

C. related to Fig 1B. Detailed information of genes targeted by HIF-1α to upstream regulators.

D. related to Fig 1C. Detailed information on the category of carbohydrate metabolism.

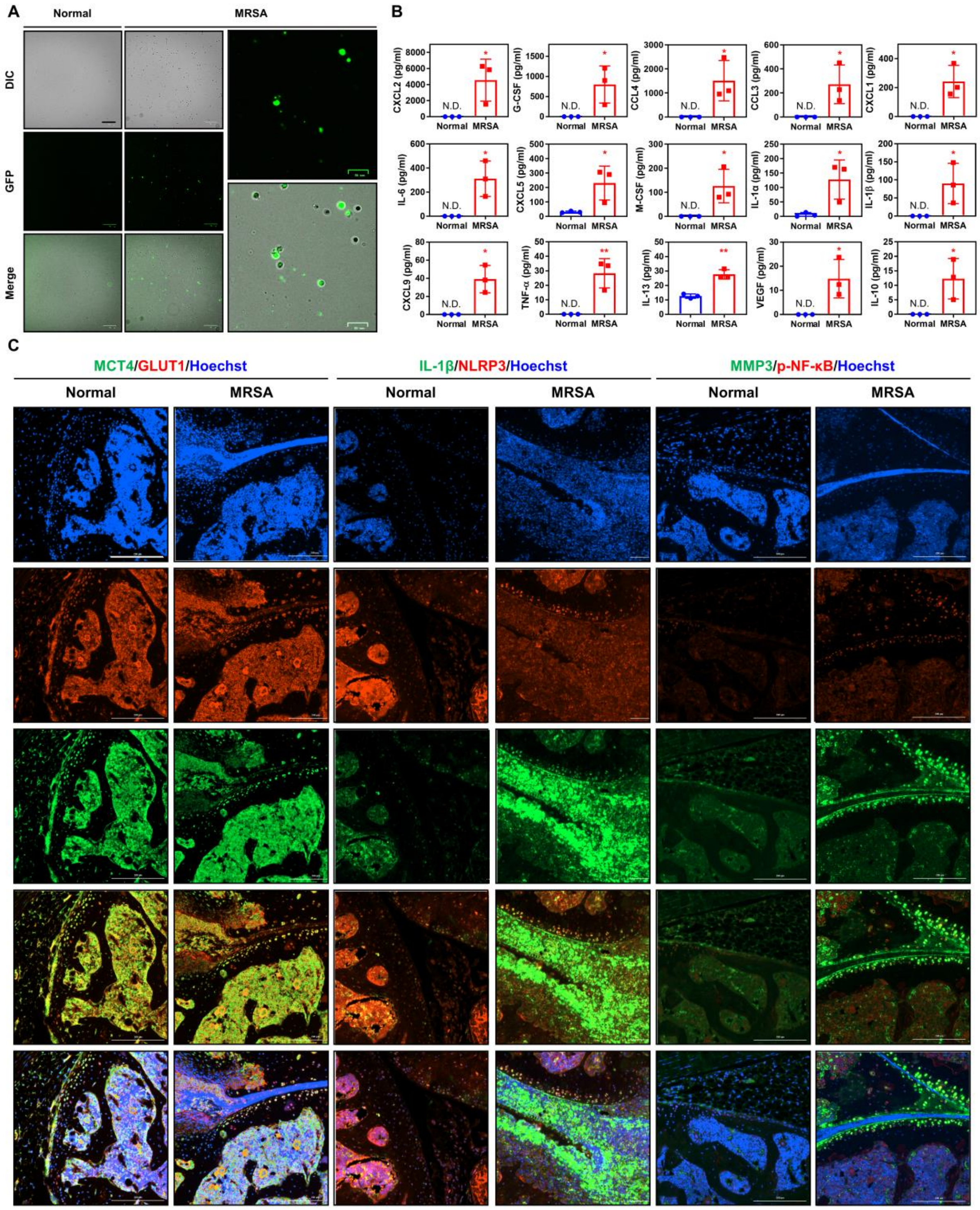

Appendix Fig. S2 Septic arthritis caused by MRSA infection induces inflammation and glycolysis, which is related to Fig 1.

A. Analysis results of synovial fluid derived from MRSA-infected septic arthritis, which is related to Fig 1H. MRSA expressive of green fluorescent protein (GFP) in immune cells infiltrating into synovial fluid was detected (Scale bar: 100 μm).

B. Changes in the expression profiles of pro-inflammatory factors within the articular space, including cytokines, chemokines, and growth factors; related to Fig 1J. Error bars show means ± SD with individual data points (*n* = 3 per group). Two-tailed unpaired *t*-test analysis was conducted to determine statistical significance (*p<0.05 or **p< 0.01; N.D. = not detected).

C. Analysis results of co-expression and -localization levels of MCT4, GLUT1, IL-1β, NLRP3, MMP3, p-NF-κB (Scale bar: 200 μm), which is related to Fig 1L.

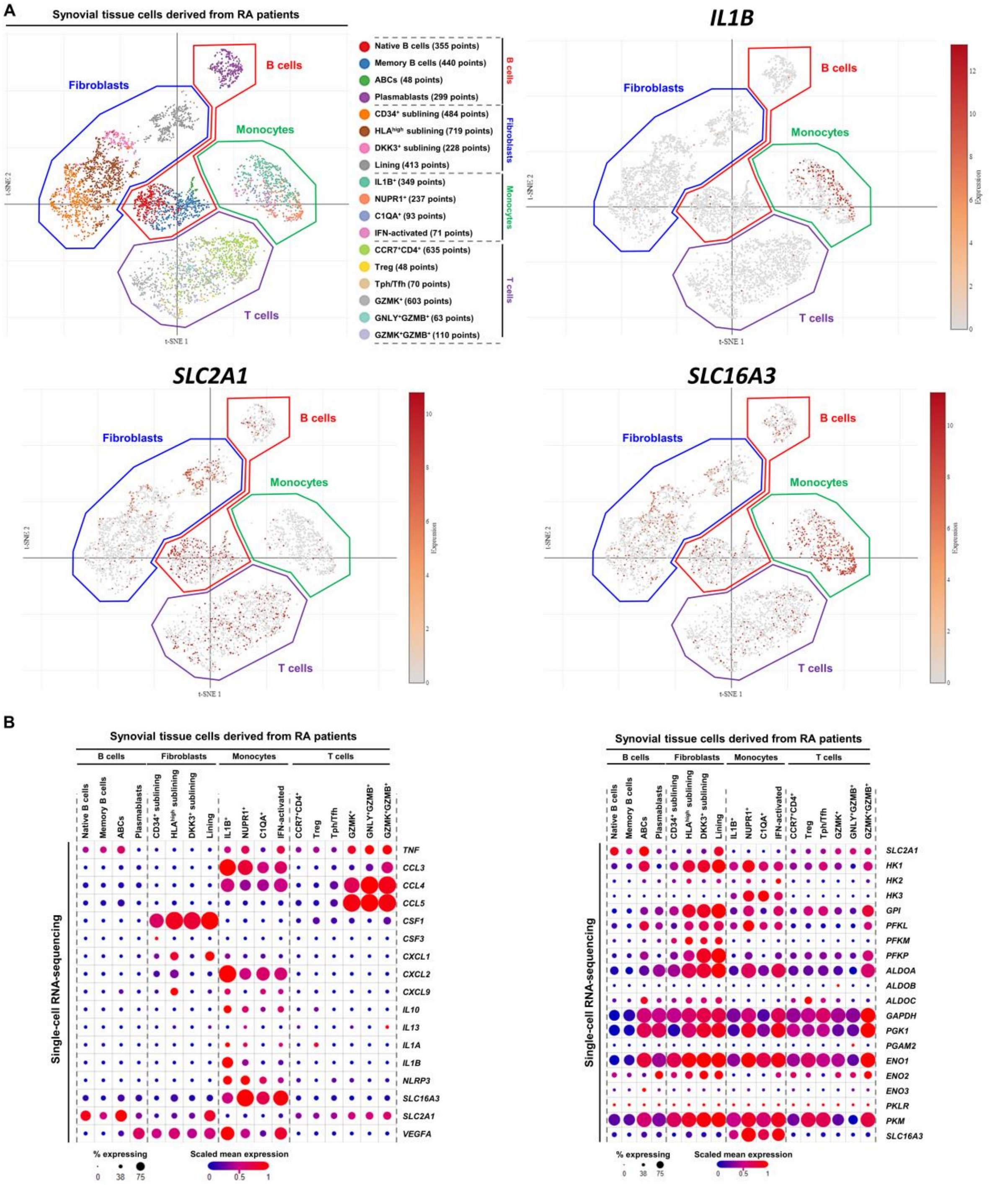

Appendix Fig. S3 Single-cell RNA-sequencing results from synovial tissue—including different types of B cells, fibroblasts, monocytes, and T cells—derived from rheumatoid arthritis patients.

A. t-distributed stochastic neighbor embedding (t-SNE) clustering map of *IL1B*, *SLC2A1*, and *SLC16A3* in synovial tissue cells including different types of B cells, fibroblasts, monocytes, and T cells.

B. Dot plot showing representative marker genes, which are associated with inflammation and glycolysis, from single-cell RNA-sequencing analysis of synovial tissue samples derived from rheumatoid arthritis patients.

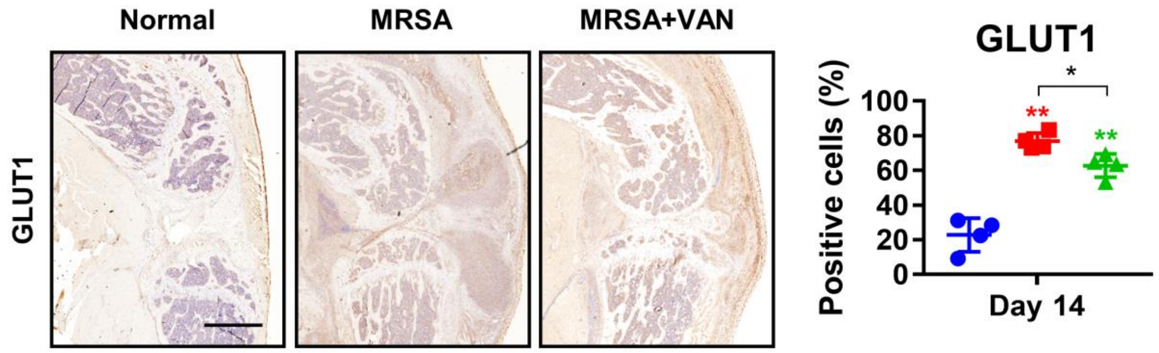

Appendix Fig. S4 GLUT1 expression using immunohistochemistry from antibiotic treatment of septic arthritis, which is related to Fig 2F.

The expression of GLUT1 at 14 days was detected and the percentage of positively staining cells determined (Scale bar: 1,000 μm). *In vivo* experiments were repeated twice per group. Error bars show means ± SD with individual data points (n = 4 per group). One-way ANOVA with Tukey's post hoc analysis was conducted to determine statistical significance (*p<0.05 or **p< 0.01).

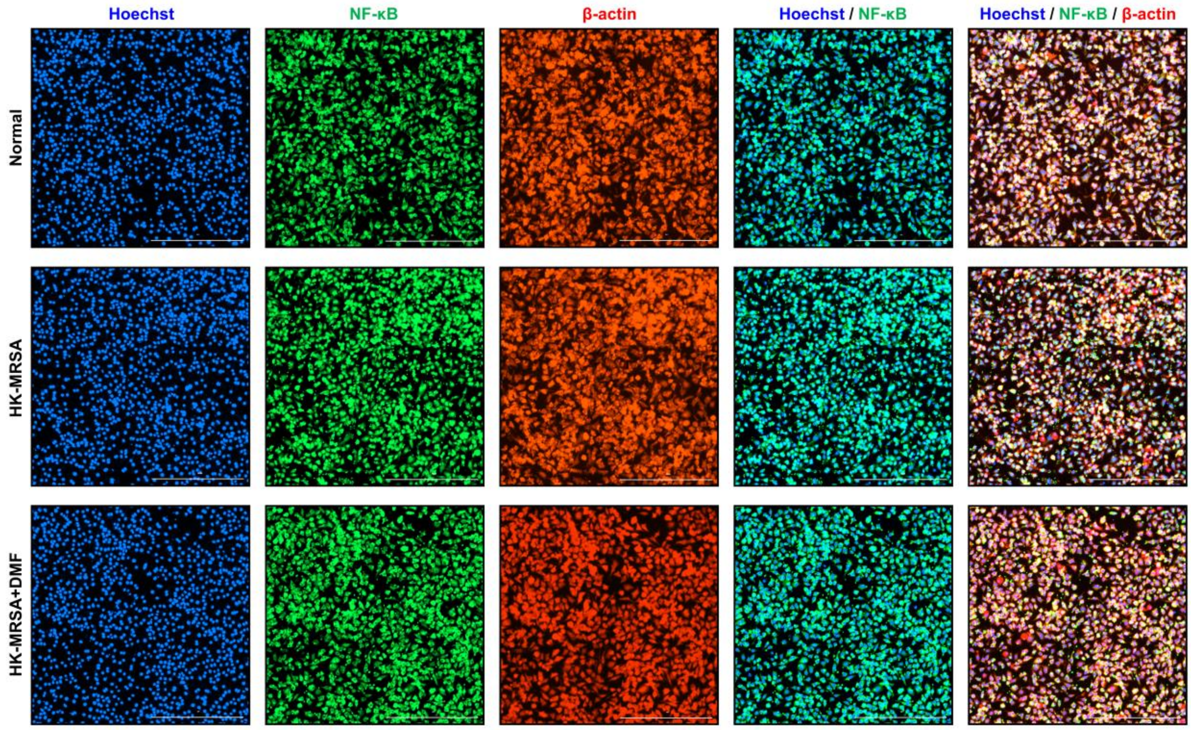

Appendix Fig. S5 Analysis of NF-κB translocation into the nucleus, which is related to Fig 4F.

RAW264.7 cells were treated with DMF (20 μM) for 1 hour and then infected with HK-MRSA (4×10^6 CFU) for 30 min. The translocation of NF-κB into the nucleus was measured and analyzed; β-actin was used for cell staining and Hoechst was used for nuclear staining (Scale bar: 300 μm).

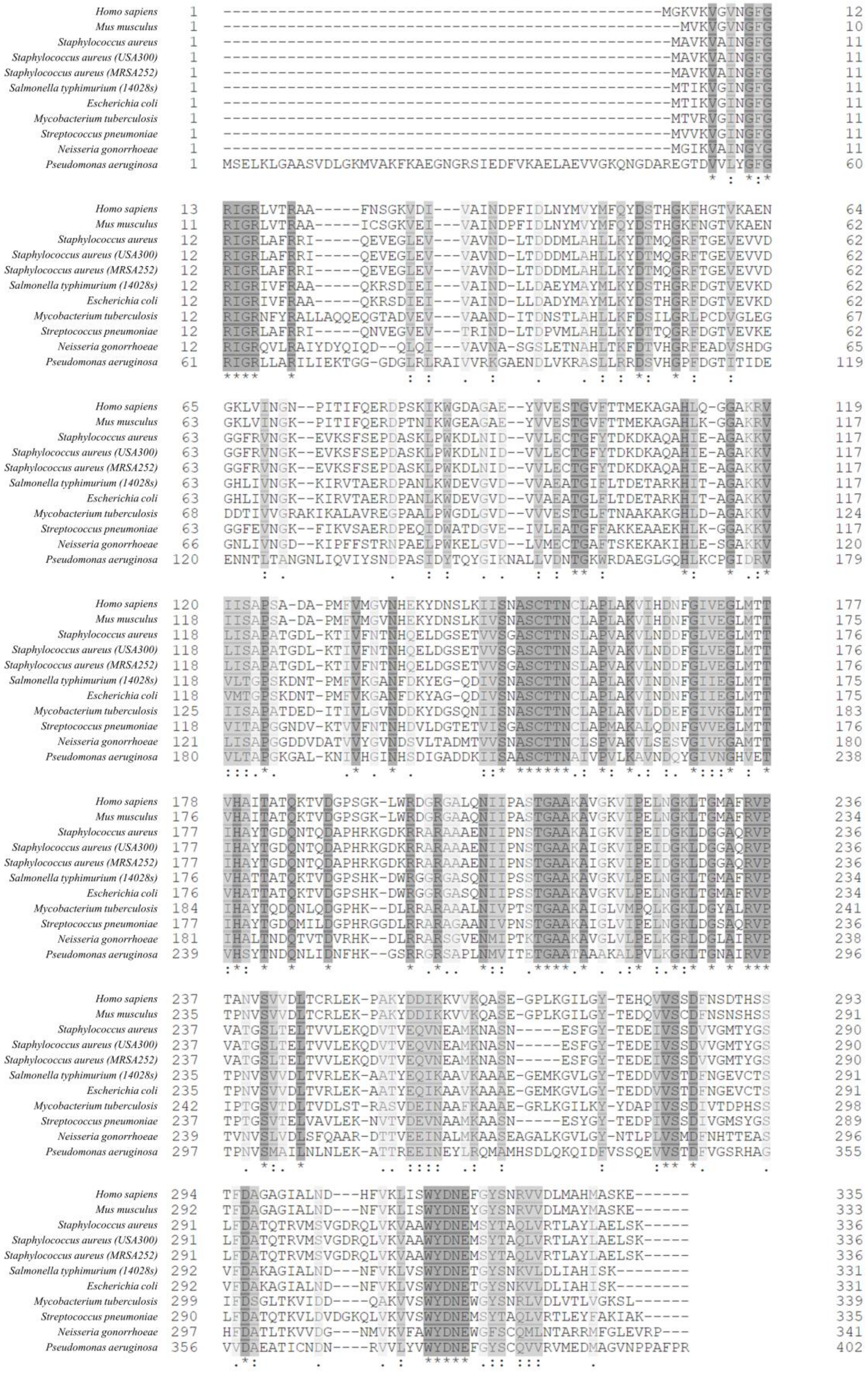

Appendix Fig. S6 Analysis of alignment of an amino acid sequence of GAPDH, which is related to Fig 5D.

Alignment of an amino acid sequence of GAPDH in *Homo sapiens, Mus musculus, Pseudomonas aeruginosa, Neisseria gonorrhoeae, Salmonella typhimurium, Escherichia coli, Mycobacterium tuberculosis, Staphylococcus aureus, Staphylococcus aureus* (USA300), *Staphylococcus aureus* (MRSA252), and *Streptococcus pneumoniae*.
