# Supplementary material for: Concurrent targeting of glycolysis in bacteria and host cell inflammation in septic arthritis
Source: EMBO Mol Med. 2022 Nov 10;14(12):e15284. doi: 10.15252/emmm.202115284 (PMC9728052; doi:10.15252/emmm.202115284)
Supplement: Supplementary file 2 — Expanded View Figures PDF [file EMMM-14-e15284-s003.pdf]

Expanded View Figures

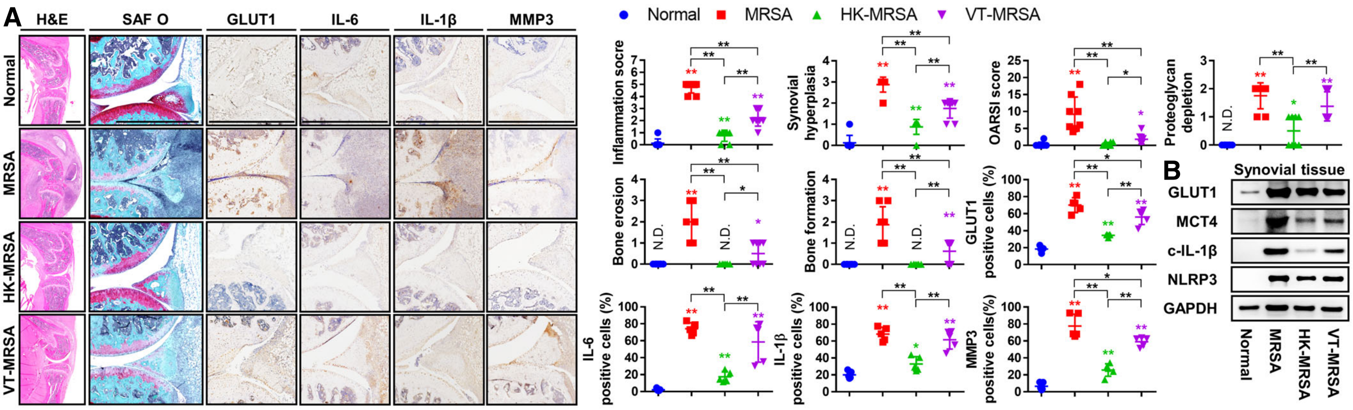

Figure EV1. Inactive or antibiotic-treated MRSA induces inflammatory arthritis along with glycolysis.

C57BL/6J mice were subcutaneously infected with MRSA (4×10^6 CFU), heat-killed MRSA (HK-MRSA), and vancomycin-treated MRSA (VT-MRSA) and sacrificed at 7 days ($n = 8$ per group).

A Paraffin-embedded tissues were sectioned and measured with respect to inflammation score, synovial hyperplasia, OARSI score, proteoglycan depletion, bone erosion, and bone formation (Scale bar: 1,000 μm). Expression of GLUT1, IL-6, IL-1β, and MMP3 was detected, and the percentages of positively staining cells were determined.

B Expression of GLUT1, MCT4, cleaved-IL-1β, and NLRP3 in synovial tissue was measured with GAPDH as a loading control.

Data information: *In vivo* experiments were repeated in at least two independent experiments. Error bars show means ± SD with individual data points. One-way ANOVA with Tukey's *post hoc* analysis was conducted to determine statistical significance (*$P < 0.05$ or **$P < 0.01$; N.D. = not detected; N.S. = not significant).
Source data are available online for this figure.

 © 2022 The Authors

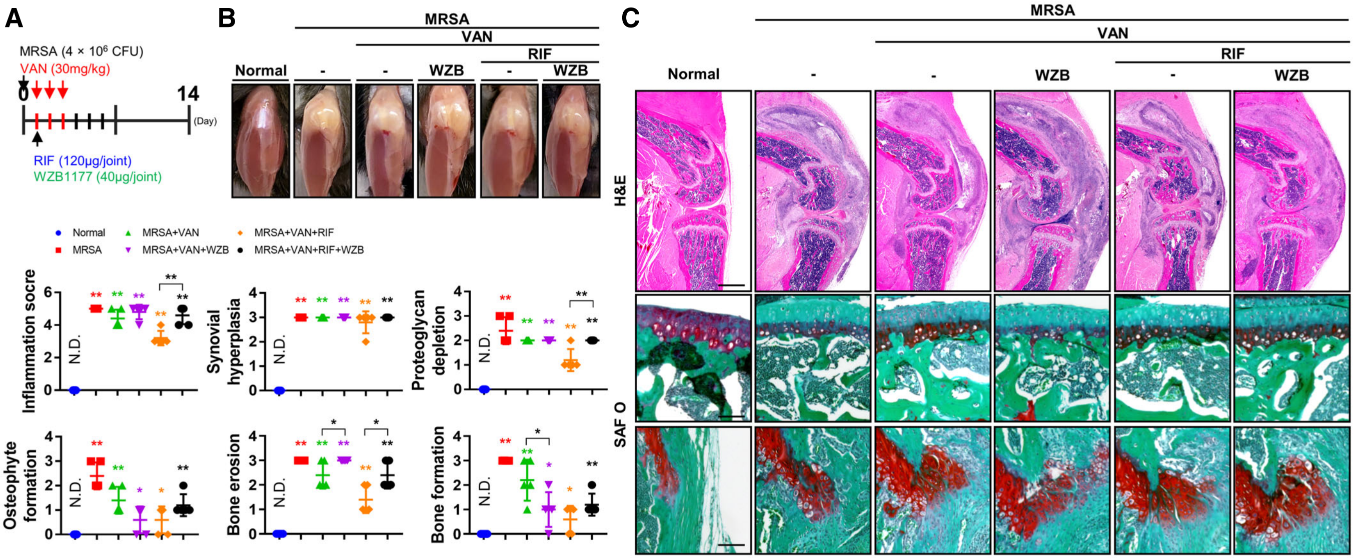

Figure EV2. Adjuvant glucose uptake inhibitor treatment exacerbates the effects of combined antibiotic treatment in septic arthritis.

A C57BL/6J mice were subcutaneously treated with vancomycin (30 mg/kg) for 3 days following MRSA (4×10^6 CFU) infection. After MRSA infection, rifampin (120 μg/joint) and/or WZB1177 (40 μg/joint) was intraarticularly injected and sacrificed at 14 days ($n = 5$ per group).

B Physiological changes were observed, and representative images were generated.

C Paraffin-embedded tissues were sectioned and measured with respect to inflammation score, synovial hyperplasia, proteoglycan depletion, osteophyte formation, bone erosion, and bone formation (Scale bar: 1,000 or 100 μm).

Data information: *In vivo* experiments were repeated in at least two independent experiments. Error bars show means ± SD with individual data points. One-way ANOVA with Tukey's *post hoc* analysis was conducted to determine statistical significance (*$P < 0.05$ or **$P < 0.01$; N.D. = not detected).

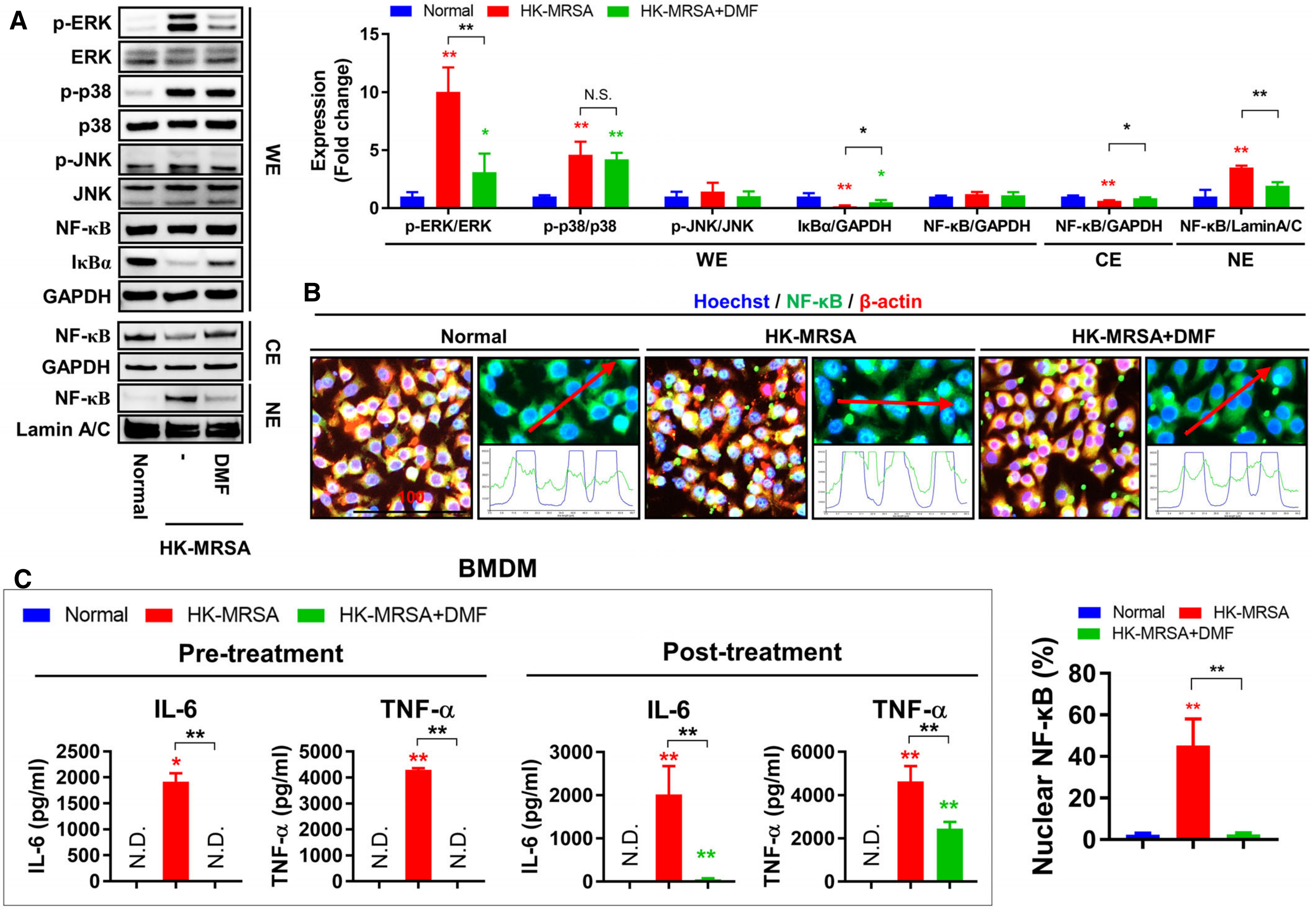

Figure EV3. DMF normalizes the generation of inflammation mediated by NF-κB and ERK signaling pathways.

A, B RAW264.7 cells were treated with DMF (20 μM) for 1 h and then infected with HK-MRSA (4×10^{6} CFU) for 30 min. (A) Expression of p-ERK, ERK, p-p38, p38, p-JNK, JNK, NF-κB, and IκBα in the whole protein extraction (WE) and expression of NF-κB in both cytoplasmic protein extraction (CE) and nuclear protein extraction (NE) were measured; GAPDH was used as a loading control for WE and CE, and Lamin A/C was used as a loading control for NE. (B) The translocation of NF-κB into the nucleus was measured and analyzed; β-actin was used for cell staining and Hoechst was used for nuclear staining (Scale bar: 100 μm). See Appendix Fig S5 for individual images.

C BMDM cells were treated with DMF (50 μg/ml) for 1 h and infected with HK-MRSA (4×10^{6} CFU) for 24 h, which we termed DMF pretreatment. BMDM cells were infected with HK-MRSA (4×10^{6} CFU) for 1 h and treated with DMF (50 μg/ml) for 24 h, which we termed DMF posttreatment. Production of IL-6 and TNF-α were measured.

Data information: *In vitro* experiments were repeated at least three times with representative results. Error bars show means ± SD with individual data points. Two-tailed unpaired *t*-test analysis and one-way ANOVA with Tukey's *post hoc* analysis were conducted to determine statistical significance (*$P < 0.05$ or **$P < 0.01$; N.D. = not detected).
Source data are available online for this figure.

Figure EV4. DMF inhibits the growth of numerous types of bacteria.

A MRSA (4×10^{6} CFU) was cultured in LB broth with or without DMF (140, 420, and 700 μg/ml). Absorbance and GFP intensity were measured at 1-h intervals for 24 h; vancomycin (2.5 mg/ml) was used as the positive control and DMSO as the negative control. Images depict MRSA expressive of green fluorescent protein (GFP) (Scale bar: 1,000 μm). See Movie EV1 for MRSA expressing GFP at individual time-points. Biofilm formation was measured for 24 h, with color changes shown in representative images.

B *Pseudomonas aeruginosa* (PA01) was cultured in TSB broth, and *Streptococcus pneumoniae* (TIGER4) was cultured in THB broth with or without DMF (43.75, 87.5, and 175 μg/ml). Absorbance was measured at 20-min intervals for 5 h with DMSO as the negative control.

Data information: *In vitro* experiments were repeated at least three times with representative results. *Ex vivo* experiments were repeated twice. Error bars show means ± SD with individual data points. Two-tailed unpaired *t*-test analysis was conducted to determine statistical significance (*$P < 0.05$ or **$P < 0.01$ or ***$P < 0.001$ or ****$P < 0.0001$; N.D. = not detected; N.S. = not significant).

© 2022 The Authors

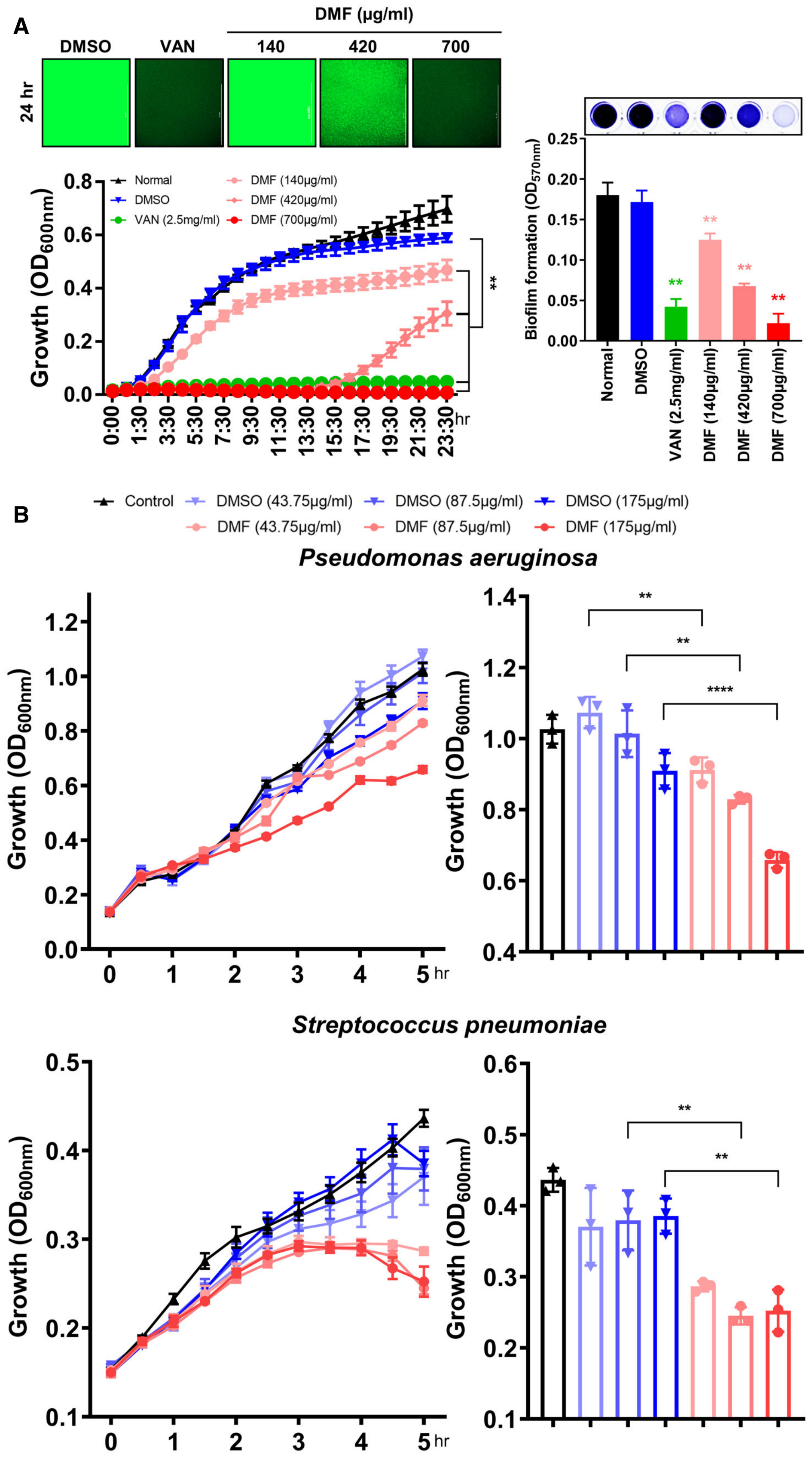
A
DMF (µg/ml)
DMSO
VAN
140
420
700
24 hr
Normal
DMSO
VAN (2.5mg/ml)
DMF (140µg/ml)
DMF (420µg/ml)
DMF (700µg/ml)
Growth (OD600nm)
hr
**
Biofilm formation (OD570nm)
B
Control
DMSO (43.75µg/ml)
DMSO (87.5µg/ml)
DMSO (175µg/ml)
DMF (43.75µg/ml)
DMF (87.5µg/ml)
DMF (175µg/ml)
Pseudomonas aeruginosa
Streptococcus pneumoniae
****

Figure EV4.

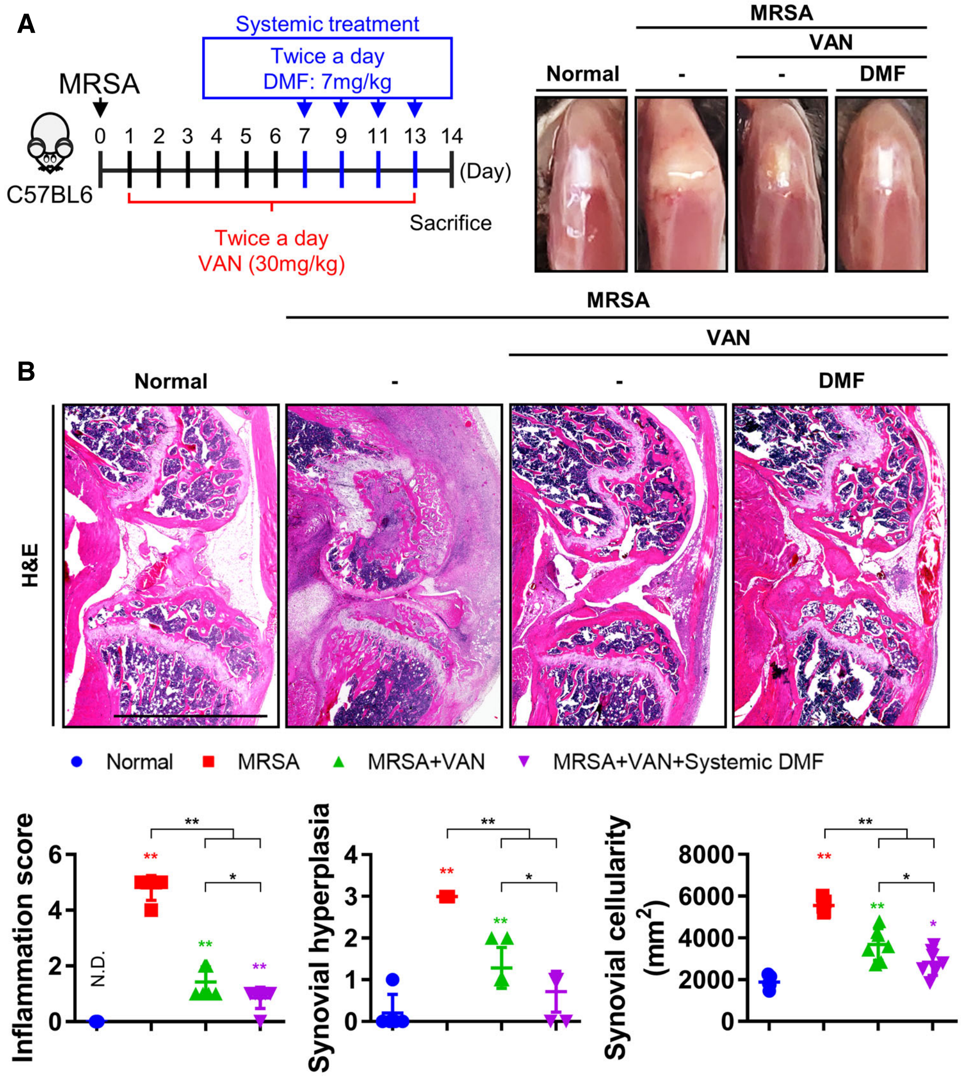

Figure EV5. Adjuvant DMF treatment with vancomycin treatment improves the prognosis of MRSA-induced septic arthritis.

C57BL/6J mice were subcutaneously treated with vancomycin (30 mg/kg) twice daily for 13 days following MRSA (8×10^6 CFU) infection (n = 5–7 per group). At day 7, DMF (7 mg/kg) was subcutaneously administered twice daily at 2-day intervals for a total of 4 times.

A Observed physiological changes were identified and compared between groups.

B Paraffin-embedded tissues were sectioned and measured with respect to inflammation score, synovial hyperplasia, and synovial cellularity (Scale bar: 2,000 μm).

Data information: *In vivo* experiments were repeated twice per group. Error bars show means ± SD with individual data points. One-way ANOVA with Tukey's *post hoc* analysis was conducted to determine statistical significance (*P < 0.05 or **P < 0.01; N.D. = not detected).

 © 2022 The Authors
