# Supplementary figures and images for: Concurrent targeting of glycolysis in bacteria and host cell inflammation in septic arthritis
Source: EMBO Mol Med. 2022 Nov 10;14(12):e15284. doi: 10.15252/emmm.202115284 (PMC9728052; doi:10.15252/emmm.202115284)

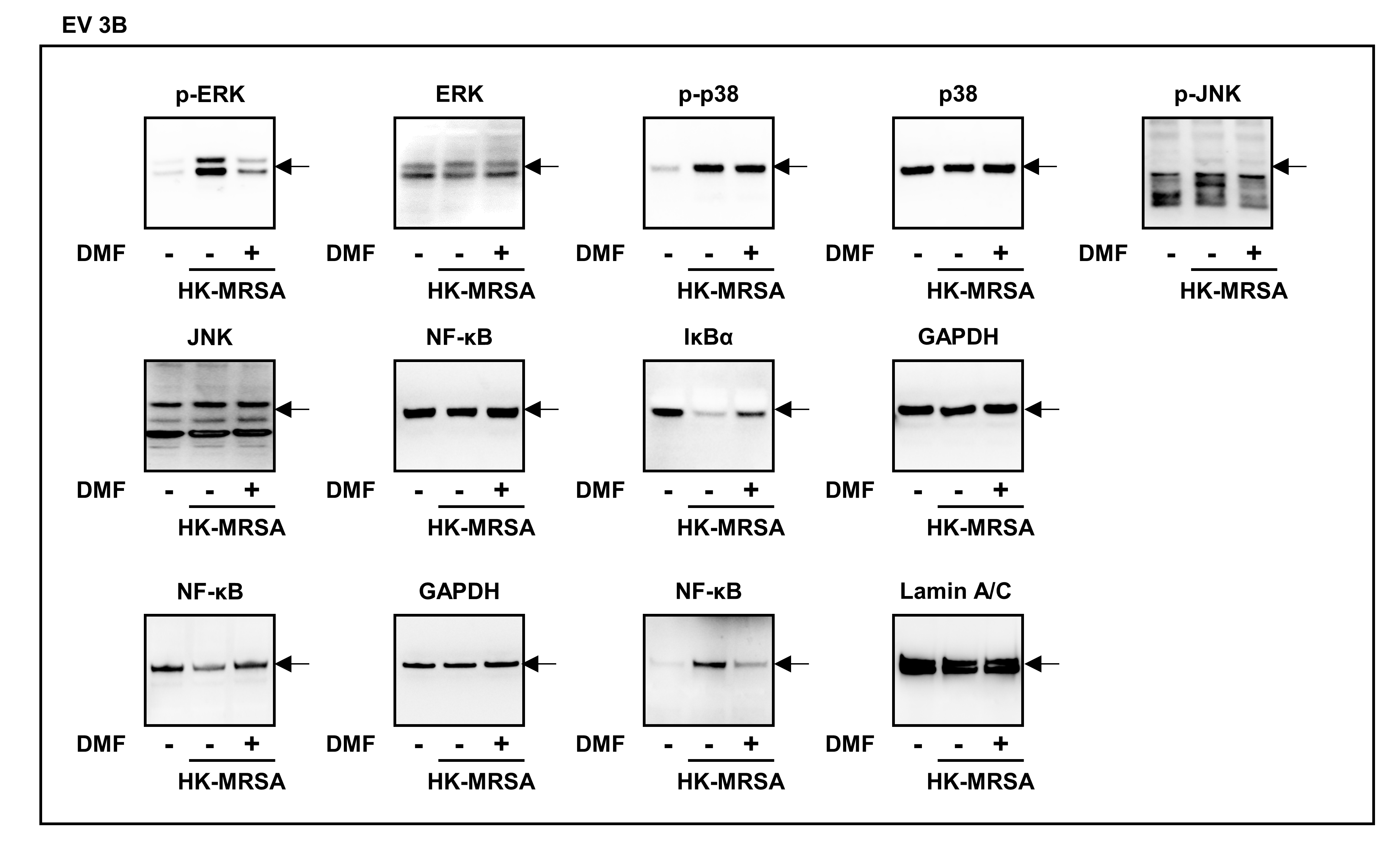

Supplement: Supplementary file 5 — Source Data for Expanded View [file EMMM-14-e15284-s006.zip › Fig_EV_3B.TIF]

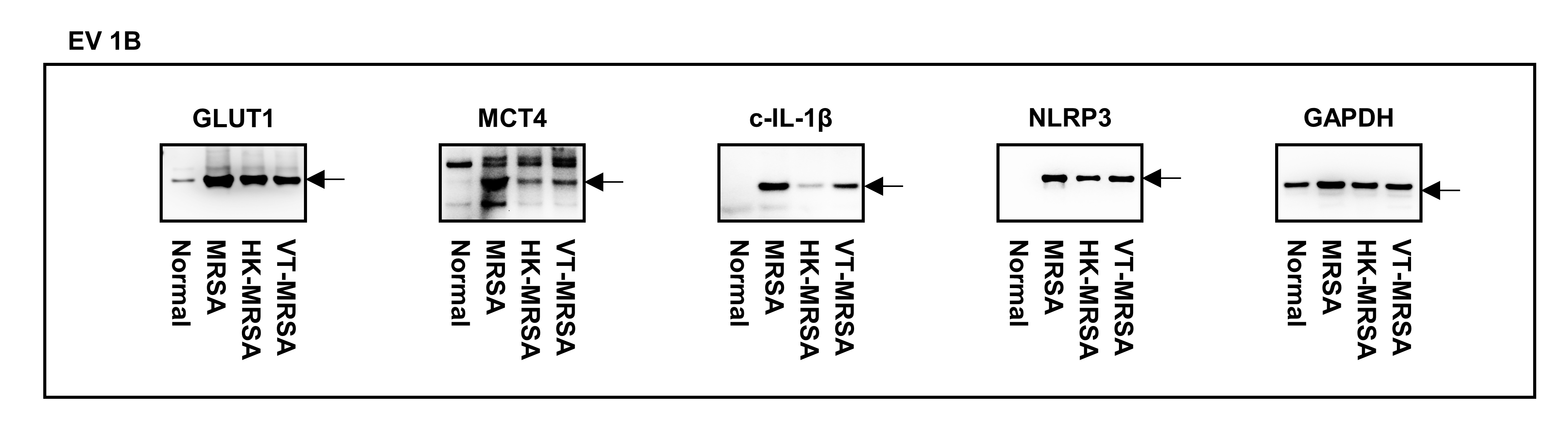

Supplement: Supplementary file 5 — Source Data for Expanded View [file EMMM-14-e15284-s006.zip › Fig_EV_1B.TIF]

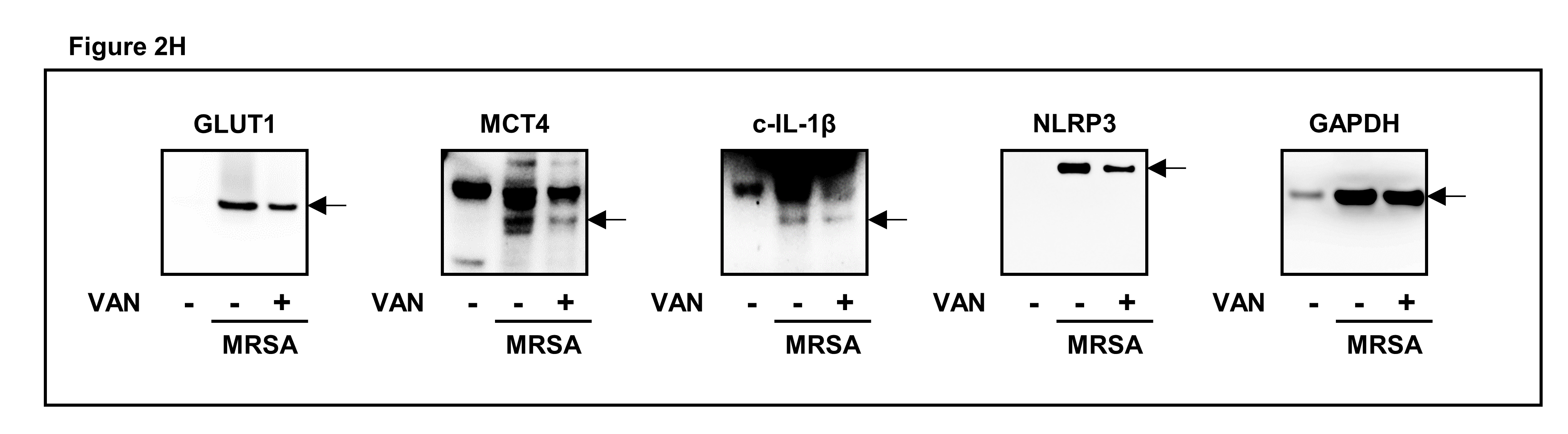

Supplement: Supplementary file 7 — Source Data for Figure 2 [file EMMM-14-e15284-s005.tif]

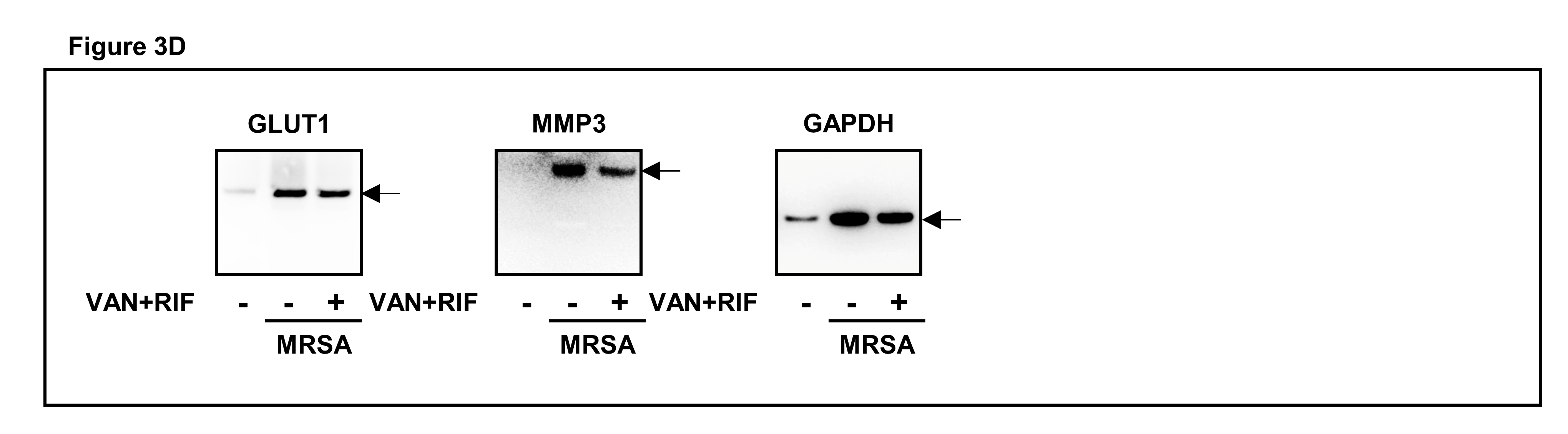

Supplement: Supplementary file 8 — Source Data for Figure 3 [file EMMM-14-e15284-s008.tif]
