# Supplementary material for: Concurrent targeting of glycolysis in bacteria and host cell inflammation in septic arthritis
Source: EMBO Mol Med. 2022 Nov 10;14(12):e15284. doi: 10.15252/emmm.202115284 (PMC9728052; doi:10.15252/emmm.202115284)
Supplement: Supplementary file 9 [file EMMM-14-e15284-s004.pdf]

EMBO Molecular Medicine

Concurrent targeting of glycolysis in bacteria and host cell inflammation in septic arthritis

Francis Lee, Hyuk-Kwon Kwon, Kristin Yu, Sean Cahill, Kareme Alder, Christopher Dussik, Sang-hun Kim, Lokesh Sharma, Jungho Back, and Irvin Oh
DOI: 10.15252/emmm.202115284

Corresponding author: Francis Lee (francis.lee@yale.edu)

Review Timeline:

Submission Date:	25th Feb 22
Editorial Decision:	24th Mar 22
Revision Received:	15th Sep 22
Editorial Decision:	4th Oct 22
Revision Received:	14th Oct 22
Accepted:	18th Oct 22

Editor: Zeljko Durdevic

Transaction Report:

(Note: With the exception of the correction of typographical or spelling errors that could be a source of ambiguity, letters and reports are not edited. Depending on transfer agreements, referee reports obtained elsewhere may or may not be included in this compilation. Referee reports are anonymous unless the Referee chooses to sign their reports.)

24th Mar 2022

Dear Prof. Lee,

Thank you for the submission of your manuscript to EMBO Molecular Medicine. We have now received feedback from the three reviewers who agreed to evaluate your manuscript. As you will see from the reports below, the referees acknowledge the interest of the study but also raise important concerns that should be addressed in a major revision. Focus of the revision should be in increasing the sample size, biological replicates, clarifying the effect of DMF on inflammation and restructuring the figures.

Further consideration of a revision that addresses reviewers' concerns in full will entail a second round of review. EMBO Molecular Medicine encourages a single round of revision only and therefore, acceptance or rejection of the manuscript will depend on the completeness of your responses included in the next, final version of the manuscript. For this reason, and to save you from any frustrations in the end, I would strongly advise against returning an incomplete revision.

We would welcome the submission of a revised version within six months for further consideration. Please let us know if you require longer to complete the revision.

Please use this link to login to the manuscript system and submit your revision: https://embomolmed.msubmit.net/cgi-bin/main.plex

I look forward to receiving your revised manuscript.

Yours sincerely,

Zeljko Durdevic

Zeljko Durdevic
Editor
EMBO Molecular Medicine

***** Reviewer's comments *****

Referee #1 (Remarks for Author):

The manuscript presents a comprehensive study on the treatment of septic arthritis in a mouse model by intervention in glycolysis.
The studies are done in mice and ex vivo using human cartilage, whole blood, and synovial fluid.
Methods are clearly presented and reproducible.
Results are solid.
Figures are overwhelming.
Conclusions are supported by the results and properly discussed.

Referee #2 (Comments on Novelty/Model System for Author):

1-Some of the sample sizes were on the smaller (n=4 in vivo for exmaple) size and it is not clear if multiple independent experiments were conducted.
2-A novel observation, though targeting bacterial metabolism is not a surprising outcome

Referee #2 (Remarks for Author):

In this study the authors investigated the targeting of glycolysis to improve the outcome of septic arthritis. They showed that a drug, DMF, that targets glycolysis in S. aureus was able to inhibit its capacity to grow as well as induce inflammation in immune cells. While the in vitro data is solid, some additional experiments are required to confirm the utility of DMF with existing therapies in vivo and confirm some of its antimicrobial properties.

Treatment of mice with DMF was after the first 7 days of antibiotics. Thus, it is hard to compare the effectiveness of this treatment given the different treatment conditions. How does this outcome compare if mice are given antibiotic therapy on days 7-14 also? How does antibiotic therapy alone compare to DMF alone? These fundamental comparisons are missing from the study to truly conclude whether the addition of DMF to existing treatment strategies is advantageous.

Figure 2. Day 7 to 14. What is the cfu by day 7 after vancomycin treatment? Is the inflammation etc observed by day 14 just outgrowth from the uneliminated bacteria once the vancomycin treatment has stopped?

In the growth assays, DMF inhibited growth but there was no difference with the addition of vancomycin. Growth assays with lower concentrations would get to the point of whether antibiotics with DMF are suitable adjunctive therapies.

Utilizing a mutant in glycolysis from the S. aureus transposon library collection to demonstrate that DMF does not have any effect on its growth would confirm that this is indeed the target pathway.

With the fluorescence assay it is hard to know that its extracellular vs intracellular, repeating these experiments with an antibiotic intracellular protection assay and quantifying the outcome would establish this.

It was concluded that DMF influenced the outcome due to both the inhibition of MRSA growth but the reduction in inflammation as a result of immune cell stimulation. In the in vivo model it is difficult to dissect these two form one another. Repeating the

infection with a compound known to suppress inflammation and observe the outcome to infection, ie phenocopy DMF would support this conclusion. Alternatively, performing a sterile model of arthritis to demonstrate the utility of DMF in reducing inflammation induced sequalae.

Referee #3 (Comments on Novelty/Model System for Author):

The manuscript addresses the important clinical problem of septic arthritis for which treatment is hampered by the emergence of antimicrobial resistance and the persistence of bacteria in the joint. The experiments are well designed and performed and the findings are novel. However, the manuscript and particularly Figures are so dense in data that it needs to be heavily refined to be accessible to a broad audience. The adequacy of the animal model is not justified - in particular the reproducibility is not discussed nor how much the model reflects the human disease biology.

Referee #3 (Remarks for Author):

Septic arthritis, the infection of the joint with bacteria, is considered a medical emergency. The bacterium S. aureus is the major cause of septic arthritis, but treatment with antibiotics is complicated due to the emergence of antimicrobial resistance and the intrinsic capacity of S. aureus to enter and survive inside eukaryotic cells. The authors argue that inflammatory markers and glycolysis products (such as lactate) are hallmarks of S. aureus induced septic arthritis, and that these properties continue in the synovial environment after antibiotic treatment. The authors also argue that therapy with a drug that targets the glycolysis pathways reduced the production of proinflammatory markers, inhibited S. aureus replication and improved the efficacy of antibiotic treatment in the joint environment. The experiments and well designed and the results have clinical relevance. This is a potentially an important paper that would move the field forwards in the development of novel approaches to treat septic arthritis caused by S. aureus. Unfortunately, the data are unsatisfactorily presented and poorly communicated in part. This severely limits the accessibility of the work to a broad audience. This problem and others need to be addressed further.
1. There is an overwhelming amount of data in each Figure. The crowdedness of the Figures means that the simple message of each Figure becomes lost in complex presentation of data. There are some Figures that are too small. There is also a lack of detail for some important Figures that makes the data hard to interpret. My suggestion is that the authors carefully consider which data is important to tell the story of each Figure and then simplify the Figures.
2. The transitions and logic for moving between sections of the manuscript are not justified clearly to the reader. Line 172-208 - there is no rationale explained for why this question was addressed following the closure of Fig 1. Currently, I do not understand why this work is included. Likewise, Lines 212-219 do no justify why it is important to perform the subsequent study or provide a hypothesis.
3. Mouse models
1. There is no discussion of the context of the mouse models. The mouse models are the result of injection of large numbers of S. aureus into the knee joint - this is not reflective of the human disease. Furthermore, S. aureus is known to produce many virulence and immune evasion factors that specifically target human but not mouse innate immune cells that are important in the pathogenesis of septic arthritis, such as neutrophils.
2. How reproducible is the collected from the mouse model? In Fig 1K there is a inflammation score of 1-2 ascribed to mice. In Fig 2D - the inflammation score is 4-5 for mice infected with MRSA. In Fig 1K the synovial hyperplasia score is 1-2, but in 2E is it
3. It appears there is a lot of variation in the progress of septic arthritis in this model - what are the explanations for this?
4. Statistical analysis. In some experiments it is unclear how statistical analysis has been performed - particularly when there are undetectable values in control groups. For example, in Fig1D - how has statistical analysis been performed for IL-6 and TNF-a if these cytokines are non-detectable in the control samples? Likewise, 1K and other figures in the manuscript.
5. Line 127-130, clarification on whether the experiment is utilizing human or mouse samples would be beneficial
6. Fig 1F and Line 142-145 - slc2a and scl16a gene expression decreases over time but this is not mentioned or discussed. What does this mean? Is this reflective of human disease?
7. Line 154 "In a severe model...." What is this model? How is it different to the previously used model in Fig in 1E/F/H/I.
8. Fig 5B and lines 297 -298. "DMF inhibited the regrowth of MRSA". This data does not quantify MRSA regrowth as it is not measuring MRSA viability.
9. Fig 5G- too small and should be confirmed for multiple MRSA / S. aureus strains. Did DMF also impair growth of other bacteria as analysed in Fig 5D.
10. Lines 417-420 "Therefore, DMF inhibits diverse types of bacterial species....." The authors have not tested this in their study and the statement is unsupported.
11. Line 416 - "gram" should be "Gram".

***** Reviewer's comments *****

Referee #1 (Remarks for Author):

The manuscript presents a comprehensive study on the treatment of septic arthritis in a mouse model by intervention in glycolysis.
The studies are done in mice and ex vivo using human cartilage, whole blood, and synovial fluid.
Methods are clearly presented and reproducible.
Results are solid.
Figures are overwhelming.
Conclusions are supported by the results and properly discussed.
Response: We appreciate for your kind comments about our work. We apologize for the overwhelming array of figures provided and have tried to modify the graphics included to be less overwhelming.

Referee #2 (Comments on Novelty/Model System for Author):

1-Some of the sample sizes were on the smaller (n=4 in vivo for exmaple) size and it is not clear if multiple independent experiments were conducted.
2-A novel observation, though targeting bacterial metabolism is not a surprising outcome
Response: We appreciate your thoughtful comments. In accordance with the guidelines of our institution's animal research ethics committee, experiments were conducted using the minimum number of animals necessary. Per the reviewer's suggestion, we increased the sample size of the experiments performed and mentioned replicate experiments that were carried out.

Referee #2 (Remarks for Author):

In this study the authors investigated the targeting of glycolysis to improve the outcome of septic arthritis. They showed that a drug, DMF, that targets glycolysis in S. aureus was able to inhibit its capacity to grow as well as induce inflammation in immune cells. While the in vitro data is solid, some additional experiments are required to confirm the utility of DMF with existing therapies in vivo and confirm some of its antimicrobial properties.

Treatment of mice with DMF was after the first 7 days of antibiotics. Thus, it is hard to compare the effectiveness of this treatment given the different treatment conditions. How does this outcome compare if mice are given antibiotic therapy on days 7-14 also? How does antibiotic therapy alone compare to DMF alone? These fundamental comparisons are missing from the study to truly conclude whether the addition of DMF to existing treatment strategies is advantageous.
Response: We apologize for the confusion in conveying the meaning of the results. We did aim to verify the effect of the combination and post-treatment of DMF under the antibiotic therapy typically used in clinical practice does not verify the therapeutic effect of septic arthritis using DMF alone. Therefore, as shown in Figure 6A-C, we validated a model in which DMF was administered systemically or intraarticularly in 3-day intervals for a total of three times after combined antibiotic therapy was systemically administered for 3 days. In addition, as shown in Figure 6D and E, we validated a model in which DMF was systemically administered 4 times every other day during the systemic administration of antibiotics. These results showed that the adjuvant administration of DMF relieved symptoms of septic arthritis when compared to the antibiotic treatment group, which result in support that adjuvant DMF treatment was more effective in septic arthritis. Accordingly, as the reviewer recommended, we modified the paragraph of the manuscript to support the audience with a more clear understanding of the design and results of the experiment.

Figure 2. Day 7 to 14. What is the cfu by day 7 after vancomycin treatment? Is the inflammation etc observed by day 14 just outgrowth from the uneliminated bacteria once the vancomycin treatment has stopped?

Response: We apologize for t making the results too complicated to understand. We measured the CFU in synovial fluid and synovial tissue at 7 and 14 days after vancomycin treatment and this information has been included in Figure 2E. At these timepoints, a small number of MRSA remained even after antibiotic treatment. Thus, we hypothesized that the inflammatory symptoms were caused by the small number of remaining MRSA remaining following antibiotic treatment or even inactivated MRSA. This hypothesis was verified using HK-MRSA and VT-MRSA and demonstrated inflammation and cartilage disruption in both infections (Fig. 2I). These results suggest that small colonies of remaining, viable bacteria or even inactivated bacteria following antibiotic treatment can incite inflammation. We corrected the images and descriptions in the manuscript so that the relevant results are clearly visible.

In the growth assays, DMF inhibited growth but there was no difference with the addition of vancomycin. Growth assays with lower concentrations would get to the point of whether antibiotics with DMF are suitable adjunctive therapies.
Response: We apologize for any confusion with the results. We verified MRSA growth inhibition by DMF at concentrations of 140-700 µg in a dose-dependent manner. Although MRSA growth inhibition was significantly higher using high concentrations of DMF, we confirmed that DMF at a concentration of 140 µg/mL still significantly reduced the growth of MRSA. We used vancomycin, a specific MRSA antibiotic according to CDC guidelines, as a positive control and observed a near complete inhibition of MRSA growth, as expected, though not inflammation. As such, we demonstrate that antibiotics are effective in inactivating bacterial (MRSA) replication, but inflammation persists regardless of antibiotic treatment. Similar results were obtained using other bacterial species. Our results provided a rationale for the bacteriostatic effect of DMF and the relevant portions of the manuscript and figures were updated accordingly.

Utilizing a mutant in glycolysis from the S. aureus transposon library collection to demonstrate that DMF does not have any effect on its growth would confirm that this is indeed the target pathway.
Response: We appreciate your suggestion to elevate the quality of the manuscript. We experimented with the effect of DMF on the GapA mutant strain of *S. aureus*. We observed that growth inhibition of *S. aureus* by DMF was interrupted in GapA mutant *S. aureus*, suggesting that DMF inhibits the growth of *S. aureus* by targeting GAPDH. These additional results are added to the Figure and explained in the manuscript.

With the fluorescence assay it is hard to know that its extracellular vs intracellular, repeating these experiments with an antibiotic intracellular protection assay and quantifying the outcome would establish this.
Response: We apologize for any confusion this set of experiments caused. We used vancomycin, which was effective against extracellular MRSA but not against intracellular

MRSA because it is incapable of intracellular penetration, for the antibiotic intracellular protection assay as requested by the reviewer. In the group treated with vancomycin, extracellular MRSA growth was inhibited, but intracellular MRSA growth increased, as demonstrated by increased intracellular GFP signal fluorescence. However, DMF inhibited the growth of both extracellular and intracellular MRSA. To complement our results, we semi-quantitatively measured intracellular and extracellular MRSA and added these data to our results.

It was concluded that DMF influenced the outcome due to both the inhibition of MRSA growth but the reduction in inflammation as a result of immune cell stimulation. In the in vivo model it is difficult to dissect these two form one another. Repeating the infection with a compound known to suppress inflammation and observe the outcome to infection, ie phenocopy DMF would support this conclusion. Alternatively, performing a sterile model of arthritis to demonstrate the utility of DMF in reducing inflammation induced sequalae.
Response: We appreciate for this valuable suggestion. We designed a new experiment accordingly to investigate the effect of DMF in a model of monosodium urate crystals (MSU)-induced inflammatory arthritis. We demonstrated that DMF relieved the inflammation caused by MSU, which supports the utility of DMF in reducing inflammation-induced sequelae. This result was added as a figure and explained in the manuscript.

Referee #3 (Comments on Novelty/Model System for Author):

The manuscript addresses the important clinical problem of septic arthritis for which treatment is hampered by the emergence of antimicrobial resistance and the persistence of bacteria in the joint. The experiments are well designed and performed and the findings are novel. However, the manuscript and particularly Figures are so dense in data that it needs to be heavily refined to be accessible to a broad audience. The adequacy of the animal model is not justified - in particular the reproducibility is not discussed nor how much the model reflects the human disease biology.
Response: We appreciate your dedication and useful comments. We made improvements to the figures to make them accessible to and decipherable by a broad audience. In addition, we discussed the adequacy and reproducibility of animal models and how the extent to which they reflect human disease biology.

Referee #3 (Remarks for Author):

Septic arthritis, the infection of the joint with bacteria, is considered a medical emergency. The bacterium S. aureus is the major cause of septic arthritis, but treatment with antibiotics is complicated due to the emergence of antimicrobial resistance and the intrinsic capacity of S. aureus to enter and survive inside eukaryotic cells. The authors argue that inflammatory markers and glycolysis products (such as lactate) are hallmarks of S. aureus induced septic arthritis, and that these properties continue in the synovial environment after antibiotic treatment. The authors also argue that therapy with a drug that targets the glycolysis pathways reduced the production of proinflammatory markers, inhibited S. aureus replication and improved the efficacy of antibiotic treatment in the joint environment. The experiments and well designed and the results have clinical relevance. This is a potentially an important paper that would move the field forwards in the development of novel approaches to treat septic arthritis caused by S. aureus. Unfortunately, the data are unsatisfactorily presented and poorly communicated in part. This severely limits the accessibility of the work to a broad audience. This problem and others need to be addressed further.

1. There is an overwhelming amount of data in each Figure. The crowdedness of the Figures means that the simple message of each Figure becomes lost in complex presentation of data. There are some Figures that are too small. There is also a lack of detail for some important Figures that makes the data hard to interpret. My suggestion is that the authors carefully consider which data is important to tell the story of each Figure and then simplify the Figures.

Response: We apologize for the overwhelming layout of the figures that rendered them difficult to interpret. Based on the reviewer's suggestion, the figures have been modified and simplified to make them easier to interpret. (All results excluded from the main figure have been moved to EV.)

2. The transitions and logic for moving between sections of the manuscript are not justified

clearly to the reader. Line 172-208 - there is no rationale explained for why this question was addressed following the closure of Fig 1. Currently, I do not understand why this work is included. Likewise, Lines 212-219 do no justify why it is important to perform the subsequent study or provide a hypothesis.

Response: Based on the reviewer suggestions, we improved our explanation of the evidence and hypothesis for performing the subsequent studies and experiments.

3. Mouse models

1. There is no discussion of the context of the mouse models. The mouse models are the result of injection of large numbers of S. aureus into the knee joint - this is not reflective of the human disease. Furthermore, S. aureus is known to produce many virulence and immune evasion factors that specifically target human but not mouse innate immune cells that are important in the pathogenesis of septic arthritis, such as neutrophils.

Response: We appreciate for these comments. We revised the discussion section to mention and explain study limitations, including regarding the application of our model and study of associated virulence factors, which we hope to further explore in subsequent research.

2. How reproducible is the collected from the mouse model? In Fig 1K there is a inflammation score of 1-2 ascribed to mice. In Fig 2D - the inflammation score is 4-5 for mice infected with MRSA. In Fig 1K the synovial hyperplasia score is 1-2, but in 2E is it 3. It appears there is a lot of variation in the progress of septic arthritis in this model - what are the explanations for this?

Response: We apologize for the confusion associated with this section. When we constructed our model of septic arthritis, all models of infection were symptomatic, and inter-model variation was less than 15% with respect to inflammation scores. Figure 1K represents a model 1 day after infection with MRSA, and Figure 2D is a model at 7 and 14 days after infection with the same amount of MRSA. Inflammation scores and synovial hyperplasia scores were different between Figure 1K and 2D because symptoms worsen over time after infection. The manuscript was revised for clarity.

4. Statistical analysis. In some experiments it is unclear how statistical analysis has been performed - particularly when there are undetectable values in control groups. For example, in Fig1D - how has statistical analysis been performed for IL-6 and TNF-a if these cytokines are non-detectable in the control samples? Likewise, 1K and other figures in the manuscript.

Response: We apologize that this information on the statistical analyses performed was omitted. We set the undetected value to 0 and all results were analyzed using a one-way analysis of variance (ANOVA) or two-tailed unpaired *t*-test in GraphPad Prism. This information was added to the methods and figure legends.

5. Line 127-130, clarification on whether the experiment is utilizing human or mouse samples

would be beneficial

Response: We appreciate your valuable comments. For clarity, we modified the sentence in the manuscript explaining the use of these murine derived cells.

6. Fig 1F and Line 142-145 - slc2a and scl16a gene expression decreases over time but this is not mentioned or discussed. What does this mean? Is this reflective of human disease?

Response: We appreciate for these comments. Our results showed a slight decrease in the mRNA expression of GLUT1 and MCT4 and the amount of lactate in the synovial fluid over time in the setting of MRSA infection; these levels remained remarkably higher than those of the uninfected animals. We believe that this pattern tends to be markedly increased in the early stage of infection due to acute immune responses and are decreased by the removal of MRSA by immune cells and death of these responding host immune cells. Previous studies showed that the expression of GLUT1 and the amount of synovial lactate were increased in inflammatory arthritis patients, which is related to the extent of inflammatory articular cartilage damage. Although the results of GLUT1 expression have not yet been clearly presented to the best of our knowledge, the amount of synovial lactate in septic arthritis patients was significantly higher than that of other inflammatory arthritis (Berthoud *et al*, 2020; Lenski & Scherer, 2014). Therefore, based on the numerous results including synovial lactate in this study, we believe that the expression of GLUT1 remains higher than normal and will be fully reflected in human disease.

7. Line 154 "In a severe model...." What is this model? How is it different to the previously used model in Fig in 1E/F/H/I.

Response: We apologize for this oversight. The Fig.1G-K results were the result of the 1 day models following MRSA infection, and the results shown in Fig. 1L were described as representing a severe model because it was measured at 14 days after MRSA infection with joint destruction. The wording of the manuscript and figure legends has been revised for clarity.

8. Fig 5B and lines 297 -298. "DMF inhibited the regrowth of MRSA". This data does not quantify MRSA regrowth as it is not measuring MRSA viability.

Response: Thank you for these comments. We used MRSA expressive of in these *in vivo* and *in vitro* experiments. Fig. 5B measured the effect of DMF in *ex vivo* experiments using synovial fluid samples derived from MRSA-infected septic arthritis models. Therefore, the intensity of GFP was used as a surrogate for MRSA growth. However, as the reviewer's comment, the expression "regrowth" was judged to be inappropriate, so the wording was revised in the relevant sections.

9. Fig 5G- too small and should be confirmed for multiple MRSA / S. aureus strains. Did DMF

also impair growth of other bacteria as analysed in Fig 5D.

Response: The response to this comment is explained along with the response to question 10 below.

10. Lines 417-420 "Therefore, DMF inhibits diverse types of bacterial species....." The authors have not tested this in their study and the statement is unsupported.

Response: We appreciate your valuable suggestions. We enlarged the image of Fig. 5G to make it more easy to view and understand. We heeded the suggestion to measure the bacteriostatic effect of DMF using *Pseudomonas aeruginosa* and *Streptococcus pneumoniae*. This result showed that DMF inhibited the growth of different types of Gram-positive and -negative bacteria. These additional results were added to the Figure section and described in the manuscript.

11. Line 416 - "gram" should be "Gram".

Response: We apologize for the typo. The manuscript has been revised accordingly.

4th Oct 2022

Dear Prof. Lee,

Thank you for the submission of your revised manuscript to EMBO Molecular Medicine. I am pleased to inform you that we will be able to accept your manuscript pending the following final amendments:

1) Please implement suggestions from the referee #3 and further simplify the figures. A substantial portion of data could be moved into an "Appendix". Please check "Author Guidelines" for more information.
https://www.embopress.org/page/journal/17574684/authorguide#expandedview
2) Figures: There are currently 9 EV Figures. Please reorganize them to a max. of 5 EV figures by combining EV Figures with one panel into one figure, e.g. EV Figures 2, 3 and 4 could be merged into one figure. Alternatively, you could compile some EV figures in an Appendix, with a ToC and legends added, correct nomenclature is "Appendix Figure S1" etc.
3) In the main manuscript file, please do the following:
- Correct/answer the track changes suggested by our data editors by working from the attached document.
- Remove text highlight colour.
- Add callouts for Figure 6A.
- In M&M, provide the antibody dilutions that were used for each antibody.
- In M&M, statistical paragraph should reflect all information that you have filled in the Authors Checklist, especially regarding randomization, blinding, replication.
- In M&M, please include statement that the experiments with human samples conformed to the principles set out in the WMA Declaration of Helsinki and the Department of Health and Human Services Belmont Report.
- Remove "Supporting Information" list.
- Author contributions: Please remove it from the manuscript and specify author contributions in our submission system. CRediT has replaced the traditional author contributions section because it offers a systematic machine-readable author contributions format that allows for more effective research assessment. You are encouraged to use the free text boxes beneath each contributing author's name to add specific details on the author's contribution. More information is available in our guide to authors:
https://www.embopress.org/page/journal/17574684/authorguide#authorshipguidelines
- Please rename "Competing Interest" to "Disclosure Statement & Competing Interests" and move it after the "Acknowledgements". We updated our journal's competing interests policy in January 2022 and request authors to consider both actual and perceived competing interests. Please review the policy https://www.embopress.org/competing-interests and update your competing interests if necessary.
- Add data availability statement. Raw data from large-scale datasets (RNA sequencing and single-cell RNA sequencing) should be deposited in one of the relevant databases and made freely available prior the publication of the manuscript. Use the following format to report the accession number of your data:

The datasets produced in this study are available in the following databases:
[data type]: [full name of the resource] [accession number/identifier] ([doi or URL or identifiers.org/DATABASE:ACCESSION])

Please check "Author Guidelines" for more information.
https://www.embopress.org/page/journal/17574684/authorguide#availabilityofpublishedmaterial
4) Movies: Please rename movie files to Movie EV1 and EV2. Their legends should be removed from the manuscript and zipped to the corresponding movie file.
5) Synopsis:
- Please remove synopsis image and text from the main manuscript files and provide them as separate files.
- Synopsis text: In addition to bullet points, please provide a short standfirst (maximum of 300 characters, including space) and submit the synopsis text as a separate .doc file.
- Synopsis image: Please provide a separate, high-resolution 550 px-wide x (250-400)-px high .jpeg file.
- Please check your synopsis text and image and submit their final versions with your revised manuscript. Please be aware that in the proof stage minor corrections only are allowed (e.g., typos).
6) For more information: This space should be used to list relevant web links for further consultation by our readers. Could you identify some relevant ones and provide such information as well? Some examples are patient associations, relevant databases, OMIM/proteins/genes links, author's websites, etc...
7) Source data: We encourage you to include the source data for figure panels that show essential data. Numerical data should be provided as individual .xls or .csv files (including a tab describing the data). For blots or microscopy, uncropped images should be submitted (using a zip archive if multiple images need to be supplied for one panel). Please check "Author Guidelines" for more information. https://www.embopress.org/page/journal/17574684/authorguide#sourcedata
8) As part of the EMBO Publications transparent editorial process initiative (see our Editorial at http://embomolmed.embopress.org/content/2/9/329), EMBO Molecular Medicine will publish online a Review Process File (RPF) to accompany accepted manuscripts. This file will be published in conjunction with your paper and will include the anonymous referee reports, your point-by-point response and all pertinent correspondence relating to the manuscript. Let us know whether

you agree with the publication of the RPF and as here, if you want to remove or not any figures from it prior to publication. Please note that the Authors checklist will be published at the end of the RPF.
9) Please provide a point-by-point letter INCLUDING my comments as well as the reviewer's reports and your detailed responses (as Word file).

I look forward to reading a new revised version of your manuscript as soon as possible.

Yours sincerely,

Zeljko Durdevic

Zeljko Durdevic
Editor
EMBO Molecular Medicine

***** Reviewer's comments *****

Referee #2 (Remarks for Author):

The authors have been very responsive to reviewer concerns, undertaking new experiments to address the prior comments.

Referee #3 (Comments on Novelty/Model System for Author):

My major comments on the manuscript from Review 1 still stand. Principally the manuscript addresses an important clinical problem of the treatment of septic arthritis. The experiments are solid and the findings are interesting. However, the manuscript still remains so dense in content that it is not accessible to a broad audience. In particular, the Figures are overwhelming and I don't think they have been edited sufficiently to be accessible to a broad audience.

Referee #3 (Remarks for Author):

The authors have improved the transition and flow of the manuscript, as well as the details and context of the mouse models utilized in the manuscript. However, the Figures are overwhelming in data/panels. I still believe this prevents the manuscript from having clear and concise messages and being accessible to a broad audience. I further encourage the authors to simplify their Figures.

***** Reviewer's comments *****

Referee #2 (Remarks for Author):

The authors have been very responsive to reviewer concerns, undertaking new experiments to address the prior comments.

Response: We appreciate your kind comments about our manuscript.

Referee #3 (Comments on Novelty/Model System for Author):

My major comments on the manuscript from Review 1 still stand. Principally the manuscript addresses an important clinical problem of the treatment of septic arthritis. The experiments are solid and the findings are interesting. However, the manuscript still remains so dense in content that it is not accessible to a broad audience. In particular, the Figures are overwhelming and I don't think they have been edited sufficiently to be accessible to a broad audience.

Referee #3 (Remarks for Author):

The authors have improved the transition and flow of the manuscript, as well as the details and context of the mouse models utilized in the manuscript. However, the Figures are overwhelming in data/panels. I still believe this prevents the manuscript from having clear and concise messages and being accessible to a broad audience. I further encourage the authors to simplify their Figures.

Response: We apologize for the overwhelming array of figures provided and have tried to modify the graphics included to be less overwhelming.

We are pleased to inform you that your manuscript is accepted for publication and is now being sent to our publisher to be included in the next available issue of EMBO Molecular Medicine.

EMBO Press Author Checklist

Corresponding Author Name: Francis Y Lee	
Journal Submitted to: EMBO Molecular Medicine	
Manuscript Number: EMM-2021-15284	

USEFUL LINKS FOR COMPLETING THIS FORM
The EMBO Journal - Author Guidelines
EMBO Reports - Author Guidelines
Molecular Systems Biology - Author Guidelines
EMBO Molecular Medicine - Author Guidelines

Reporting Checklist for Life Science Articles (updated January 2022)
This checklist is adapted from Materials Design Analysis Reporting (MDAR) Checklist for Authors. MDAR establishes a minimum set of requirements in transparent reporting in the life sciences (see Statement of Task: 10.31222/osf.io/9sm4x). Please follow the journal's guidelines in preparing your manuscript.
Please note that a copy of this checklist will be published alongside your article.

Abridged guidelines for figures
1. Data
The data shown in figures should satisfy the following conditions:
- the data were obtained and processed according to the field's best practice and are presented to reflect the results of the experiments in an accurate and unbiased manner.
- ideally, figure panels should include only measurements that are directly comparable to each other and obtained with the same assay.
- plots include clearly labeled error bars for independent experiments and sample sizes. Unless justified, error bars should not be shown for technical replicates.
- if n<5, the individual data points from each experiment should be plotted. Any statistical test employed should be justified.
- Source Data should be included to report the data underlying figures according to the guidelines set out in the authorship guidelines on Data Presentation.

2. Captions
Each figure caption should contain the following information, for each panel where they are relevant:
- a specification of the experimental system investigated (eg cell line, species name).
- the assay(s) and method(s) used to carry out the reported observations and measurements.
- an explicit mention of the biological and chemical entity(ies) that are being measured.
- an explicit mention of the biological and chemical entity(ies) that are altered/varied/perturbed in a controlled manner.
- the exact sample size (n) for each experimental group/condition, given as a number, not a range;
- a description of the sample collection allowing the reader to understand whether the samples represent technical or biological replicates (including how many animals, litters, cultures, etc.).
- a statement of how many times the experiment shown was independently replicated in the laboratory.
- definitions of statistical methods and measures:
  - common tests, such as t-test (please specify whether paired vs. unpaired), simple χ2 tests, Wilcoxon and Mann-Whitney tests, can be unambiguously identified by name only, but more complex techniques should be described in the methods section;
  - are tests one-sided or two-sided?
  - are there adjustments for multiple comparisons?
  - exact statistical test results, e.g., P values = x but not P values < x;
  - definition of 'center values' as median or average;
  - definition of error bars as s.d. or s.e.m.

Please complete ALL of the questions below.
Select "Not Applicable" only when the requested information is not relevant for your study.

Materials

Newly Created Materials	Information included in the manuscript?	In which section is the information available? (Reagents and Tools Table, Materials and Methods, Figures, Data Availability Section)
New materials and reagents need to be available; do any restrictions apply?	Not Applicable	

Antibodies	Information included in the manuscript?	In which section is the information available? (Reagents and Tools Table, Materials and Methods, Figures, Data Availability Section)
For **antibodies** provide the following information:<br>- Commercial antibodies: RRID (if possible) or supplier name, catalogue number and or/clone number<br>- Non-commercial: RRID or citation	Yes	Materials and Methods

DNA and RNA sequences	Information included in the manuscript?	In which section is the information available? (Reagents and Tools Table, Materials and Methods, Figures, Data Availability Section)
**Short novel DNA or RNA including primers, probes**: provide the sequences.	Yes	Materials and Methods

Cell materials	Information included in the manuscript?	In which section is the information available? (Reagents and Tools Table, Materials and Methods, Figures, Data Availability Section)
**Cell lines:** Provide species information, strain. Provide accession number in repository **OR** supplier name, catalog number, clone number, and/**OR** RRID.	Yes	Materials and Methods
**Primary cultures:** Provide species, strain, sex of origin, genetic modification status.	Yes	Materials and Methods
Report if the cell lines were recently **authenticated** (e.g., by STR profiling) and tested for mycoplasma contamination.	Yes	Materials and Methods

Experimental animals	Information included in the manuscript?	In which section is the information available? (Reagents and Tools Table, Materials and Methods, Figures, Data Availability Section)
**Laboratory animals or Model organisms:** Provide species, strain, sex, age, genetic modification status. Provide accession number in repository **OR** supplier name, catalog number, clone number, **OR** RRID.	Yes	Materials and Methods
**Animal observed in or captured from the field:** Provide species, sex, and age where possible.	Yes	Materials and Methods
Please detail **housing and husbandry conditions**.	Yes	Materials and Methods

Plants and microbes	Information included in the manuscript?	In which section is the information available? (Reagents and Tools Table, Materials and Methods, Figures, Data Availability Section)
**Plants:** provide species and strain, ecotype and cultivar where relevant, unique accession number if available, and source (including location for collected wild specimens).	Not Applicable	
**Microbes:** provide species and strain, unique accession number if available, and source.	Yes	Materials and Methods

Human research participants	Information included in the manuscript?	In which section is the information available? (Reagents and Tools Table, Materials and Methods, Figures, Data Availability Section)
If collected and within the bounds of privacy constraints report on age, sex and gender or ethnicity for all study participants.	Not Applicable	

Core facilities	Information included in the manuscript?	In which section is the information available? (Reagents and Tools Table, Materials and Methods, Figures, Data Availability Section)
If your work benefited from core facilities, was their service mentioned in the acknowledgments section?	Yes	Acknowledgments section

Design

Study protocol	Information included in the manuscript?	In which section is the information available? (Reagents and Tools Table, Materials and Methods, Figures, Data Availability Section)
If study protocol has been **pre-registered, provide DOI in the manuscript**. For clinical trials, provide the trial registration number **OR** cite DOI.	Not Applicable	
Report the **clinical trial registration number** (at ClinicalTrials.gov or equivalent), where applicable.	Not Applicable	

Laboratory protocol	Information included in the manuscript?	In which section is the information available? (Reagents and Tools Table, Materials and Methods, Figures, Data Availability Section)
Provide DOI OR other citation details if **external detailed step-by-step protocols** are available.	Not Applicable	

Experimental study design and statistics	Information included in the manuscript?	In which section is the information available? (Reagents and Tools Table, Materials and Methods, Figures, Data Availability Section)
Include a statement about **sample size** estimate even if no statistical methods were used.	Yes	Materials and Methods
Were any steps taken to minimize the effects of subjective bias when allocating animals/samples to treatment (e.g. **randomization procedure**)? If yes, have they been described?	Yes	Materials and Methods
Include a statement about **blinding** even if no blinding was done.	Not Applicable	
Describe **inclusion/exclusion criteria** if samples or animals were excluded from the analysis. Were the criteria pre-established?<br><br>If sample or data points were omitted from analysis, report if this was due to attrition or intentional exclusion and provide justification.	Not Applicable	
For every figure, are **statistical tests** justified as appropriate? Do the data meet the assumptions of the tests (e.g., normal distribution)? Describe any methods used to assess it. Is there an estimate of variation within each group of data? Is the variance similar between the groups that are being statistically compared?	Yes	Figures

Sample definition and in-laboratory replication	Information included in the manuscript?	In which section is the information available? (Reagents and Tools Table, Materials and Methods, Figures, Data Availability Section)
In the figure legends: state number of times the experiment was **replicated** in laboratory.	Yes	Materials and Methods
In the figure legends: define whether data describe **technical or biological replicates**.	Yes	Materials and Methods

Ethics

Ethics	Information included in the manuscript?	In which section is the information available? (Reagents and Tools Table, Materials and Methods, Figures, Data Availability Section)
Studies involving **human participants**: State details of **authority granting ethics approval** (IRB or equivalent committee(s), provide reference number for approval.	Not Applicable	
Studies involving **human participants**: Include a statement confirming that **informed consent** was obtained from all subjects and that the experiments conformed to the principles set out in the WMA Declaration of Helsinki and the Department of Health and Human Services Belmont Report.	Not Applicable	
Studies involving **human participants:** For publication of **patient photos**, include a statement confirming that consent to publish was obtained.	Not Applicable	
Studies involving experimental **animals**: State details of **authority granting ethics approval** (IRB or equivalent committee(s), provide reference number for approval. Include a statement of compliance with ethical regulations.	Yes	Materials and Methods
Studies involving **specimen and field samples:** State if relevant **permits** obtained, provide details of authority approving study; if none were required, explain why.	Yes	Materials and Methods

Dual Use Research of Concern (DURC)	Information included in the manuscript?	In which section is the information available? (Reagents and Tools Table, Materials and Methods, Figures, Data Availability Section)
Could your study fall under dual use research restrictions? Please check biosecurity documents and list of **select agents and toxins** (CDC): https://www.selectagents.gov/sat/list.htm	Not Applicable	
If you used a select agent, is the security level of the lab appropriate and reported in the manuscript?	Not Applicable	
If a study is subject to dual use research of concern regulations, is the name of the **authority granting approval and reference number** for the regulatory approval provided in the manuscript?	Not Applicable	

Reporting

The MDAR framework recommends adoption of discipline-specific guidelines, established and endorsed through community initiatives. Journals have their own policy about requiring specific guidelines and recommendations to complement MDAR.

Adherence to community standards	Information included in the manuscript?	In which section is the information available? (Reagents and Tools Table, Materials and Methods, Figures, Data Availability Section)
State if relevant guidelines or checklists (e.g., **ICMJE, MIBBI, ARRIVE, PRISMA**) have been followed or provided.	Not Applicable	
For **tumor marker prognostic studies,** we recommend that you follow the **REMARK** reporting guidelines (see link list at top right). See author guidelines, under 'Reporting Guidelines'. Please confirm you have followed these guidelines.	Not Applicable	
For **phase II and III randomized controlled trials**, please refer to the **CONSORT** flow diagram (see link list at top right) and submit the CONSORT checklist (see link list at top right) with your submission. See author guidelines, under 'Reporting Guidelines'. Please confirm you have submitted this list.	Not Applicable	

Data Availability

Data availability	Information included in the manuscript?	In which section is the information available? (Reagents and Tools Table, Materials and Methods, Figures, Data Availability Section)
Have **primary datasets** been deposited according to the journal's guidelines (see 'Data Deposition' section) and the respective accession numbers provided in the Data Availability Section?	Yes	Materials and Methods
Were **human clinical and genomic datasets** deposited in a public access-controlled repository in accordance to ethical obligations to the patients and to the applicable consent agreement?	Not Applicable	
Are **computational models** that are central and integral to a study available without restrictions in a machine-readable form? Were the relevant accession numbers or links provided?	Not Applicable	
If publicly available data were reused, provide the respective **data citations in the reference list**.	Yes	Materials and Methods
